# Asymmetric α-Fluoroalkyl-α-Amino Acids: Recent Advances in Their Synthesis and Applications

**DOI:** 10.3390/molecules29061408

**Published:** 2024-03-21

**Authors:** Nathan Picois, Yazid Boutahri, Pierre Milbeo, Chiara Zanato, Nathalie Lensen, Grégory Chaume, Thierry Brigaud

**Affiliations:** 1 CY Cergy Paris Université, CNRS, BioCIS UMR 8076, 95000 Cergy-Pontoise, France; nathan.picois@cyu.fr (N.P.); yazid.boutahri@cyu.fr (Y.B.); pierre.milbeo@cyu.fr (P.M.); chiara.zanato@cyu.fr (C.Z.); nathalie.lensen@cyu.fr (N.L.); gregory.chaume@cyu.fr (G.C.); 2 Université Paris-Saclay, CNRS, BioCIS UMR 8076, 91400 Orsay, France

**Keywords:** amino acids, asymmetric synthesis, fluorinated compounds, peptides, medicinal chemistry, chemical biology

## Abstract

Due to the specific properties provided by fluorine atoms to biomolecules, amino acids with fluorinated side chains are of great interest for medicinal chemistry and chemical biology. Among them, α-fluoroalkyl-α-amino acids constitute a unique class of compounds. In this review, we outline the strategies adopted for their syntheses in enantiopure or enantioenriched forms and their incorporation into peptides. We then describe the consequences of the introduction of fluorine atoms in these compounds for the modulation of their hydrophobicity and the control of their conformation. Emerging applications are presented in the areas of enzyme inhibition, medicinal chemistry, hydrolytic stability of peptides, antimicrobial peptides, PET, and ^19^F NMR probes.

## 1. Introduction

Although several reviews have been published about the synthesis of fluorinated amino acids as a whole [1,2,3,4,5], none of them are specifically focused on both the synthesis of α-fluoroalkyl-α-amino acids and their applications. However, these amino acids constitute a very special class of fluorinated amino acids as their reactivity and properties are strongly influenced by the proximity of the electron-withdrawing fluorine atoms to the amino acid backbone. The main effects are to decrease the nucleophilicity of the amino group, to increase the acidity of the carboxylic acid functional group, and to modulate steric hinderance and hydrophobicity. The aim of this review is firstly to present a condensed and didactic overview of fluoroalkyl-α-amino acid synthesis updated with recent advances. Although some strategies involve monofluorinated starting materials, most of the methods for the synthesis of chiral α-monofluoroalkyl-α-amino acids are using fluorination reactions of nonfluorinated precursors. Nucleophilic (DAST, Morpho-Dast, Deoxofluor, XtalFluor-E, CsF, etc.) and electrophilic (NFSI, Selectfluor, etc.) fluorinating reagents are extensively used for their preparation. α-Difluoroalkyl-α-amino acids can be synthesized by nucleophilic difluorination of carbonyl compounds, difluoroalkylation reactions, and transformation of difluoro starting materials. Asymmetric catalysis occupies a prominent place in the preparation of α-CF_3_-α-amino acids, and a great variety of catalysts has been recently designed for highly enantioselective reductions, alkylations, and Strecker-type reactions. Chiral auxiliary-based methods proved to be very robust for the scale-up production of enantiopure α-CF_3_-α-amino acids.

The second part of this review is devoted to the diverse applications of these unnatural amino acids, including the consequences induced by their incorporation into peptides. It should be noticed that the incorporation of these amino acids in peptides is often a challenge because of the strong deactivation of their amino group due to the strong electron-withdrawing effect of the fluorine atoms. Most of these applications are taking advantage of the very special properties of fluorinated analogues of biomolecules, such as (i) increased hydrophobicity, (ii) conformational constraints, and (iii) high ^19^F NMR sensitivity. The applications α-fluoroalkyl-α-amino acids presented in this review cover many diverse domains such as enzyme inhibition, medicinal chemistry, hydrolytic stability of peptides, antimicrobial peptides, PET, and ^19^F NMR probes.

## 2. Synthesis of α-Fluoroalkyl-α-Amino Acids

### 2.1. α-Monofluoroalkyl-α-Amino Acids

The synthesis of 3-monofluoroalkyl-α-amino acids has long been studied for the interesting properties that these non-proteinogenic amino acids can induce. Their synthesis in an enantiopure form is not so easy because of their tendency to epimerize. Although several reviews concerning the synthesis of these fluorinated amino acids have already been published [1,2,3], we report here a summarized presentation of the published synthesis of 3-monofluoroalkyl-α-amino acids and we also describe in more detail recent works on novel syntheses. For each type of synthesis, only the key steps will be indicated in all schemes, as well as the final yield and the optical purity. We will focus on stereoselective syntheses leading to enantiopure or at least diastereomerically pure α-monofluoroalkyl-α-amino acids. Five methods are mainly used for the synthesis of α-monofluoroalkyl-α-amino acids: starting from fluorinated commercially available substrates; electrophilic, nucleophilic, or radical fluorination; and finally a biochemical method.

#### 2.1.1. 3-Fluoroalanine Syntheses

**Scheme 1:**
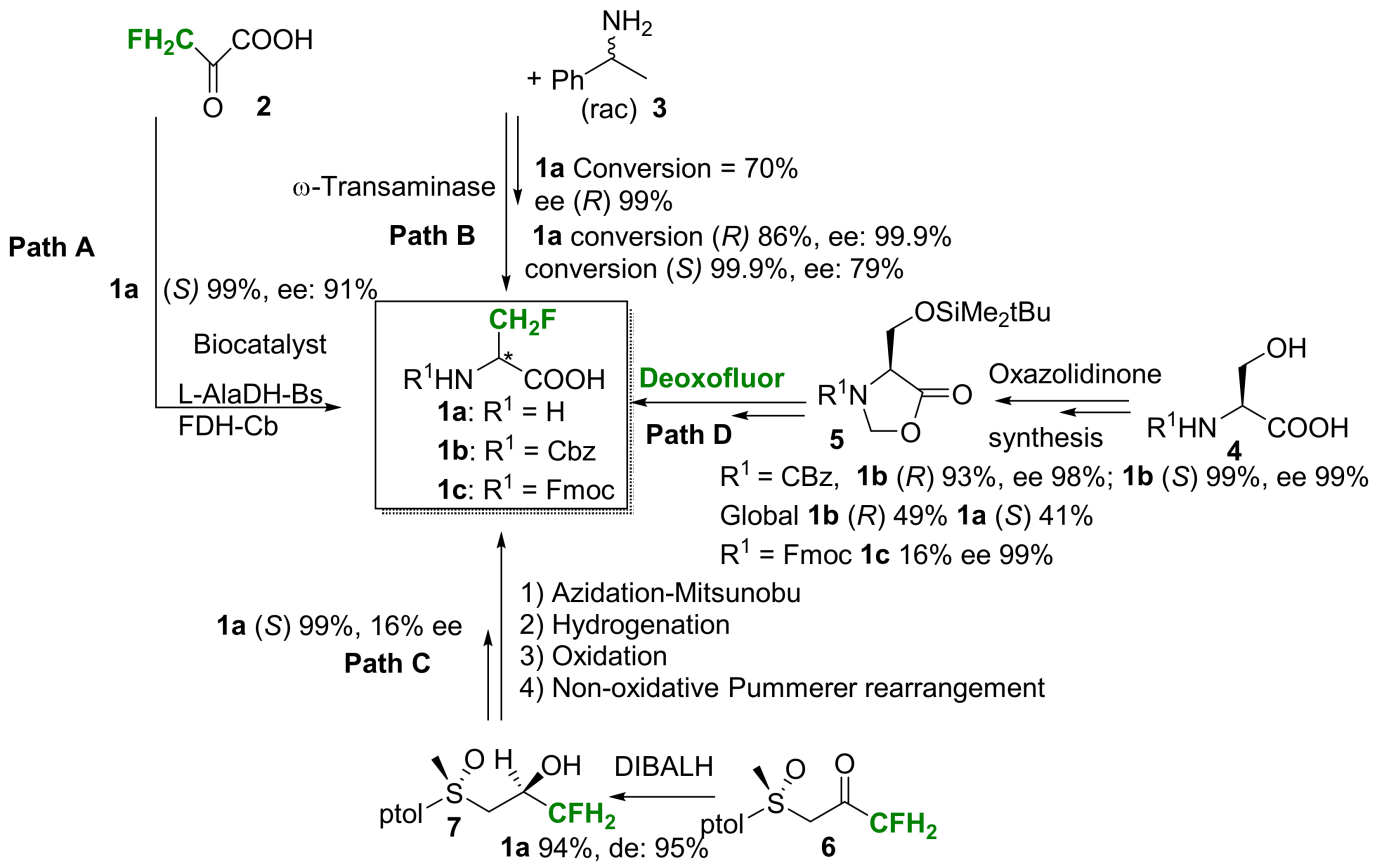
Syntheses of acyclic 3-monofluoroalkyl-α-amino acid **1** [6,7,8,9,10,11].

The synthesis of 3-fluoroalanine has been the subject of much research. The following scheme summarizes the methods used (Scheme 1). Starting from 3-fuoropyruvate **2**, the Lopez-Gallego group uses a heterogeneous biocatalyst containing two NADH-dependent alcohol dehydrogenases (l-ALaDH-Bs and FDH-Cb) for the synthesis of **1a** in a good yield and high enantiopurity. These two enzymes are co-immobilized and were integrated into a packed reactor for continuous synthesis of l-amino acids (Scheme 1 path A) [6]. The Han-Seop Bea group [7] also uses the 3-fluoropyruvate **2** as the starting substrate, which undergoes biotransformation. The ω-transaminase has high reactivity towards amino receptors such as 3-fluoropyruvate **2**. Various amino donor compounds, such as (rac) α-MBA **3**, have been used for the reductive amination of 2 using ω-transaminase. The (*R*)-3-fluoroalanine **1a** is obtained with good conversion and excellent enantiopurity via a kinetic resolution (Scheme 1 path B). Shin et al. [8] modified this method using isopropylamine as the amino donor catalyzed by the (*S*) selective Ochrobactrum anthropic OATA and its variant (*R*) selective ARmutTA; the conversion is improved to 86% for the (*R*)-3-fluoroalanine **1a** and 79% for the (*S*) enantiomer **1a** (Scheme 1 path B).

The enantiopure α-sulfinyl alcohol **7**, obtained from the 3-fluorinated-2-oxoproylsulfoxyde **6** via a diastereoselective reduction reaction using DIBALH, was used by Bravo et al. as a starting material [9]. The key step was the transformation of the alcohol function into an amino group via a Mitsunobu reaction to introduce an azide group for total enantioselectivity. Hydrogenation and oxidation followed by a non-oxidative Pummerer rearrangement led to (*S*)-**1a** (Scheme 1 path C).

Hoveyda’s group reported the synthesis of the two enantiomers of the 3-fluoroalanine **1b** and their respective *N*-methyl analogues starting from the oxazolidinone **5** obtained from L- or D-serine. Fluorodehydroxylation performed by Deoxofluor gave access to **1b** in good yields and excellent stereoselectivities (Scheme 1 path D) [10]. Voyer et al. described the first synthesis of the Fmoc-protected 3-fluoroalanine **1c**, which is compatible with SPPS, using almost the same protocol [11]. The fluorinated agent was XtalFluor-E and it was used in the presence of TEA-3 HF (Scheme 1 path D).

#### 2.1.2. Acyclic 3-Substituted 3-Fluoroalanine Syntheses

Syntheses of 3-alkyl and 3-aryl-3-monofluoro-α-amino acids have been the subject of many publications. These syntheses can be subdivided into two families: electrophilic (Selectfluor, NFSI or radical) and nucleophilic (DAST, Morpho-Dast, TBAT, Deoxofluor, XtalFluor-E, and HF) fluorination methods.

- Electrophilic fluorination

**Scheme 2:**
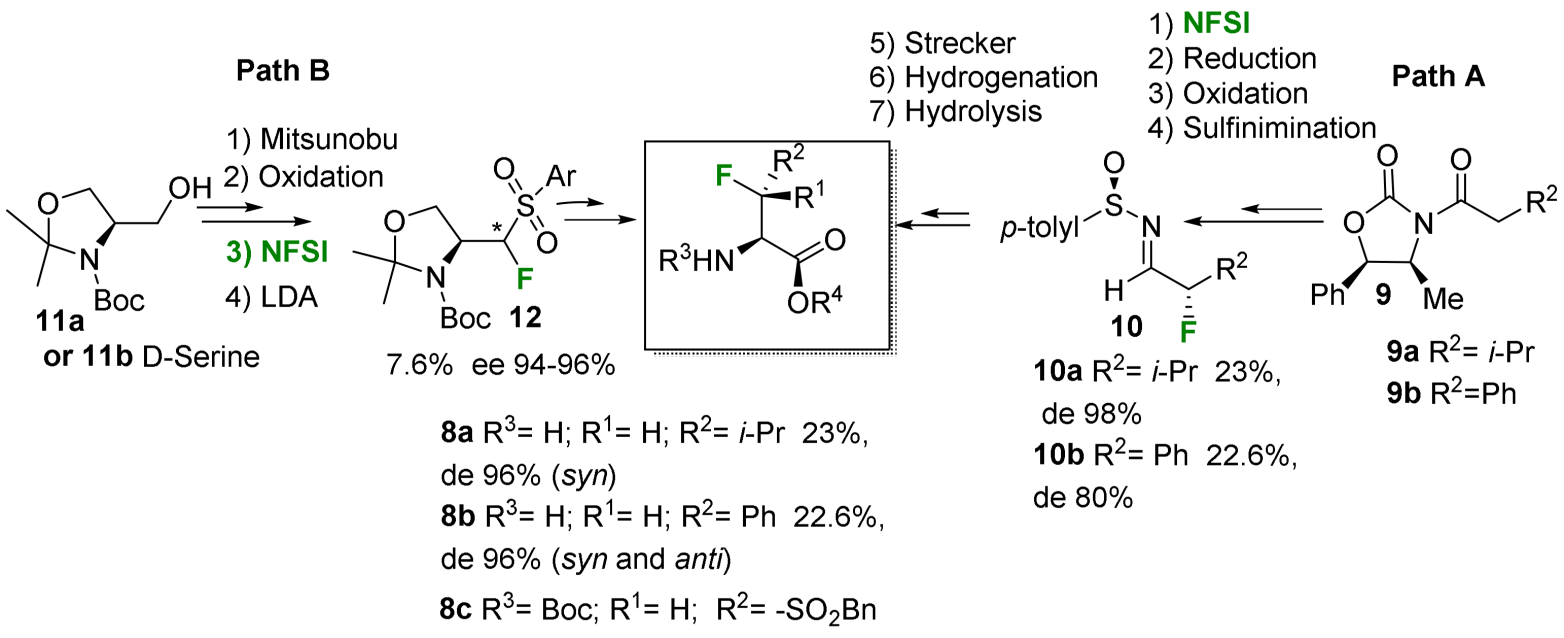
Syntheses of acyclic 3-substituted-3-fluoro-alanine using electrophilic fluorinated reagents [12,13].

Enantiopure oxazolidinones **9a-b** were used by Davis et al. for the synthesis of new intermediate α-fluoro sulfinimime building blocks **10a-b** via an electrophilic fluorination reaction [12]. An asymmetric Strecker reaction gave access to the *syn*- and *anti* 3-fluorophenylalanine **8b** (depending on the (*R*)-or (*S*)-sulfonamide used) and the *syn* 3-fluororoleucine **8a** with a good diastereomeric excess (96%) (Scheme 2 path A).

Zajc et al. described the diastereoselective fluorination of *N*-Boc arylsulfonyloxazolidines derivatives obtained from the reduced (*R*) alcohol **11a** or d-serine **11b** precursors [13]. This fluorinated *N*-Boc-arylsulfonyloxazolidine **12** was diastereoselectively epimerized at the fluorine-bearing carbon atom alpha to the sulfone to give access to the two stereoisomers of the 3-benzylsulfonyl-3-monofluoro α-amino acid **8c** (Scheme 2 path B).

**Scheme 3:**
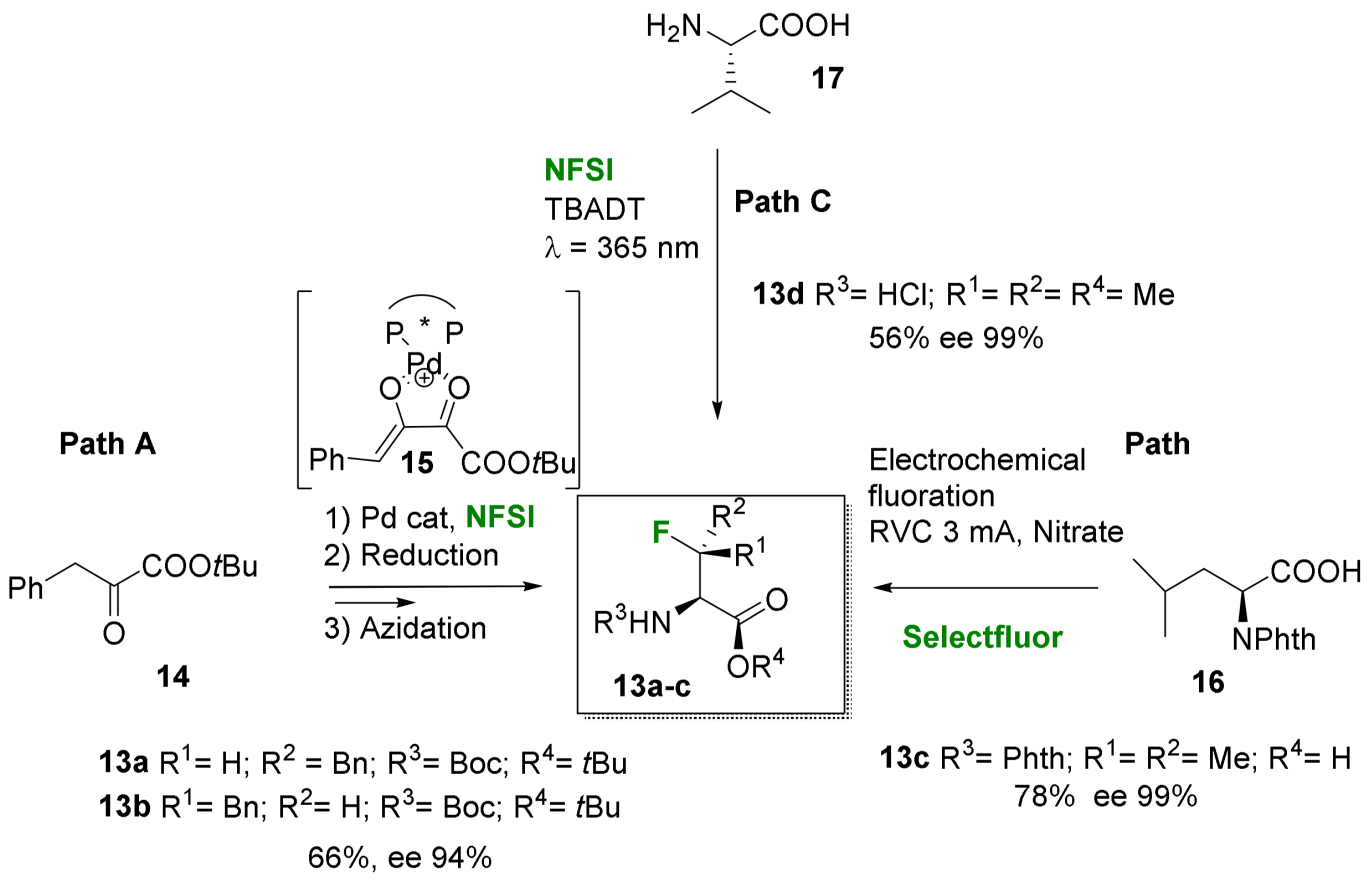
Syntheses of acyclic 3-substituted-3-monofluoro-α-amino acids [14,15,16].

Sodeoka et al. described the first example of a catalytic enantioselective monofluorination of α-keto-ester **14** via a chiral Pd-enolate intermediate **15**. The resulting fluorinated compounds were reduced into chiral β-fluoro-α-hydroxy esters and then converted into *anti* β-fluoro-α-aminoester **13a** and *syn* β-fluoro-α-aminoester **13b** via an azidation reaction (Scheme 3 path A) [14].

Electrochemical methods for alkyl C-H fluorination were described by Baran et al. Selectfluor was used as a mediator coupled to anodic oxidation in the presence of a nitrate additive. The best conditions were obtained using RVC electrodes at 3 mA. A multigram synthesis of 3-fluorovaline **13c** from L-protected valine **16** was performed in good yield (78%) and with total stereoselectivity (Scheme 3 path B) [15].

Photocatalytic fluorination of the unactivated C-H bond using the hydrogen abstracting ability of a decatungstate photocatalyst (TBADT, Na_4_W_10_O_32_) coupled with NFSI was described by Britton et al. [16]. By this way, *N*-Phth-3-fluorovaline **13d** was obtained in good yield (56%) and with total enantioselectivity in one step from valine **17** (Scheme 3 path C).

- Nucleophilic fluorination

**Scheme 4:**
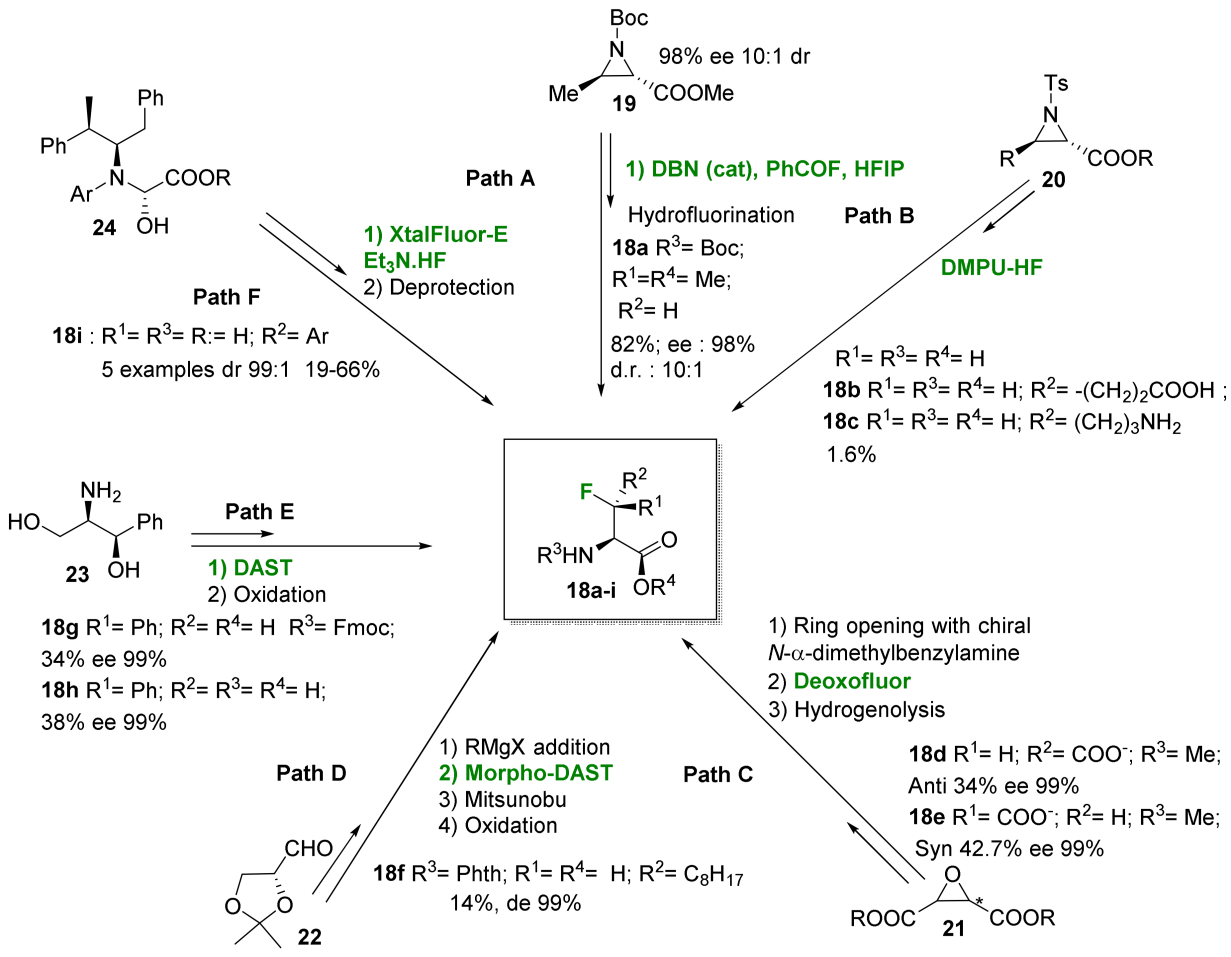
Syntheses of acyclic 3-substituted-3-monofluoro-α-amino acids using nucleophilic fluorinated reagents [17,18,19,20,21,22].

Doyle et al. described the hydrofluorination of the unsymmetrical enantiopure aziridine **19** using the in situ generation of amine-HF from benzoyl fluoride and HFIP in a Lewis base (DBN)-catalyzed reaction. This reaction is totally regioselective, diastereoselective, and with no ee erosion. Compound (*S*)-**18a** was obtained in a good yield of 82% (Scheme 4 Path A) [17].

Riss et al. used the non-racemic aziridine-2-carboxylate **20**, which was subjected to regio- and stereoselective ring opening by a nucleophilic fluoride [18]. The 2-amino-3-fluoroadipic acid **18b** and the 3-fluorolysine **18c** were obtained in low yields (Scheme 4 path B).

O’Hagan et al. started from (*S*,*S*)- or (*S*,*R*)-epoxy succinic esters **21** to afford a non-isolable intermediate aziridine via a nucleophilic cycle opening with *N*-benzyl methylamine and treatment with Deoxofluor [19]. As this fluorination occurs with complete stereoselectivity, they obtained a separable mixture of diastereomers, which gave enantiopure *syn*-**18d** and *anti*-**18e** 3-fluoro *N*-methylaspartates (Scheme 4 path C).

Fokina et al. used a commercially (*R*)-2,3-*O*-isopropylideneglyceraldehyde **22** for the introduction of chirality. Their key steps were the addition of *n*-octyl magnesium bromide to the aldehyde function of **22**, leading to the corresponding diastereomeric alcohols that were fluorinated using Morpho-DAST. The hydrolysis of the dioxolane group followed by the protection of the primary alcohol gave access to a chromatographically separable mixture of alcohols. The alcohol functions were then transformed into amino groups via a Mitsunobu reaction. In this way, the *N*-protected (2*S*,3*R*)-2-amino-3-fluoroundecanoic acid **18f** was obtained in a 14% overall yield and with pure stereoselectivity (Scheme 4 path D) [20].

Okuda et al. started from the commercially available (1*R*,2*R*)-2-amino-1-phenylpropane-1,3-diol **23**. The use of equimolar amounts of DAST and DIEA gave the expected fluorinated compound as a single diastereomer. Classical protection, deprotection, and oxidation reactions lead to 3-fluorophenylalanine analogues **18g** and **18h** (Scheme 4 path E) [21].

Davies et al. synthesized enantiopure aryl-substituted *anti*-3-fluorophenylalanine **18i** starting from α-hydroxy-β-amino ester **24** through a stereospecific rearrangement with XtalFluor-E (Scheme 4 path F) [22].

**Scheme 5:**
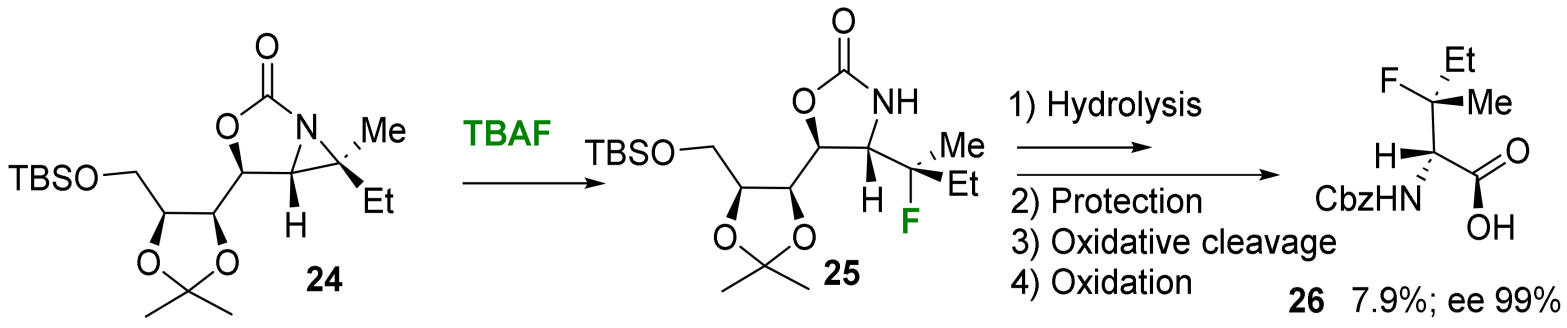
Synthesis of 3-fluoroisoleucine [23].

Recently, Chen et al. described the synthesis of optically pure α-monofluoroalkyl-α-amino acids, such as fluorinated isoleucine **26**, starting from arabinose. The formation of a diastereomerically pure 2-oxazolidinone-fused-aziridine intermediate **24** was followed by the regioselective ring opening of the aziridine with TBAF. Compound **25** gave enantiopure 3-fluorinated isoleucine **26** after few classical chemical transformations (Scheme 5) [23].

#### 2.1.3. Syntheses of Cyclic 3-Fluoro-α-Amino Acids

The synthesis of several cyclic 3-monofluoro-α-amino acids of different sizes (four-, five-, or six-membered ring) have been described.

**Scheme 6:**
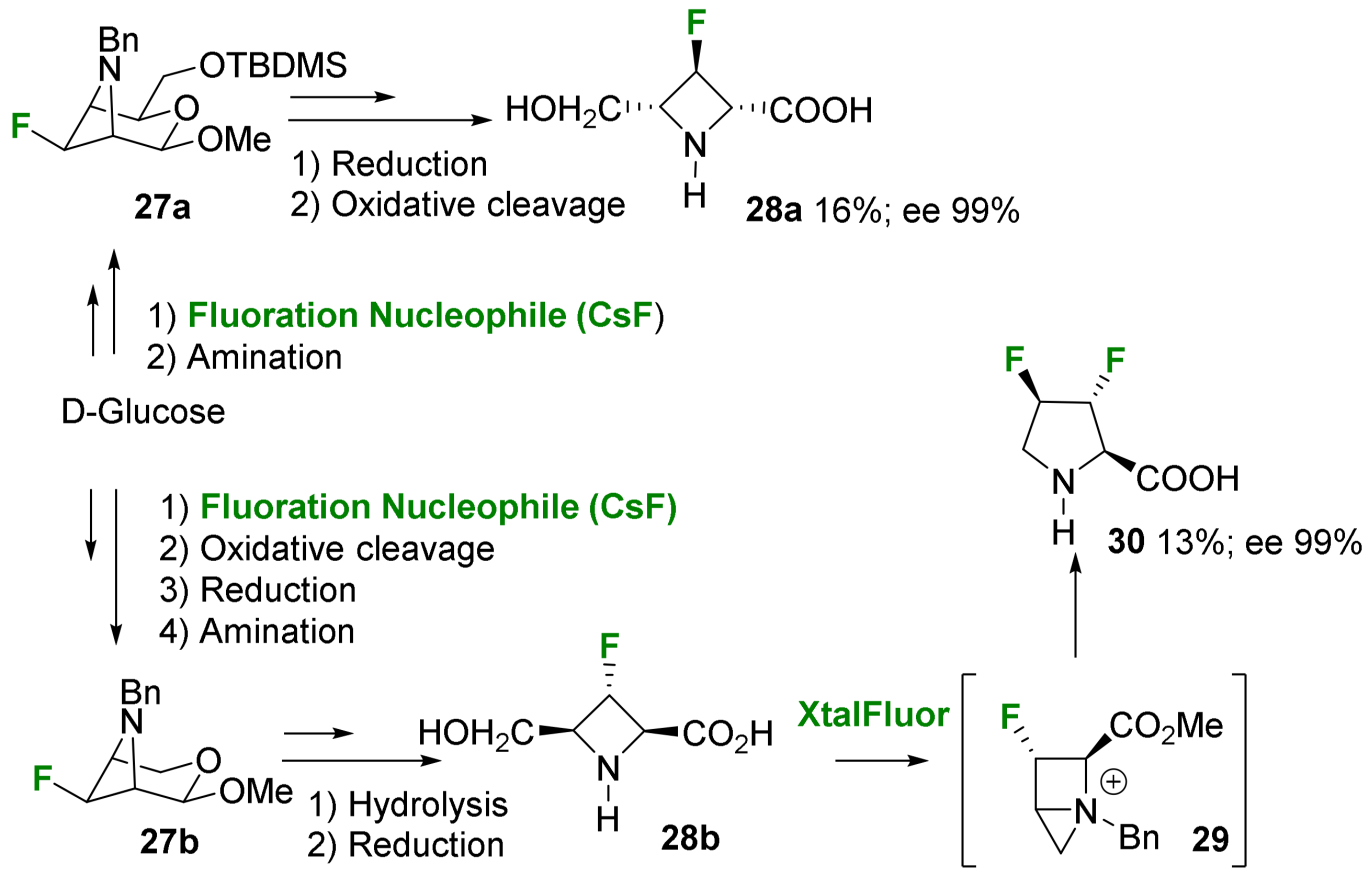
Synthesis of 3-fluoroazetidine carboxylic acid and 3, 4-difluoroproline [24].

Fleet et al. reported the synthesis of 3-fluoroazetidine carboxylic acids **28a** and **28b** and *trans*,*trans*-3,4-difluoroproline **30**. Starting from d-glucose and using a sequence of conventional carbohydrate chemical transformations and a nucleophilic fluorination with CsF, the bicylic azetidines **27a** and **27b** were formed. Each of these bicyclic intermediate structures evolved into the corresponding azetidine **28a** or **28b**. In the presence of XtalFluor, azetidine **28b** gave the *trans*,*trans*-the 3,4-difluoroproline **30** via the bicyclic aziridinium intermediate **29 [24]** (Scheme 6).

Ciulli et al. described the synthesis of the four stereoisomers of 3-fluoro-hydroxy proline **32a-d** using Selectfluor. The three stereoisomers (*R*,*S*,*R*)-**32a**, (*R*,*R*,*S*)-**32b**, (*R*,*R*,*R*)-**32c** were prepared in three steps. The fourth one, (*R*,*S*,*S*)-**32d**, was obtained via a Mitsunobu reaction [25].

**Scheme 7:**
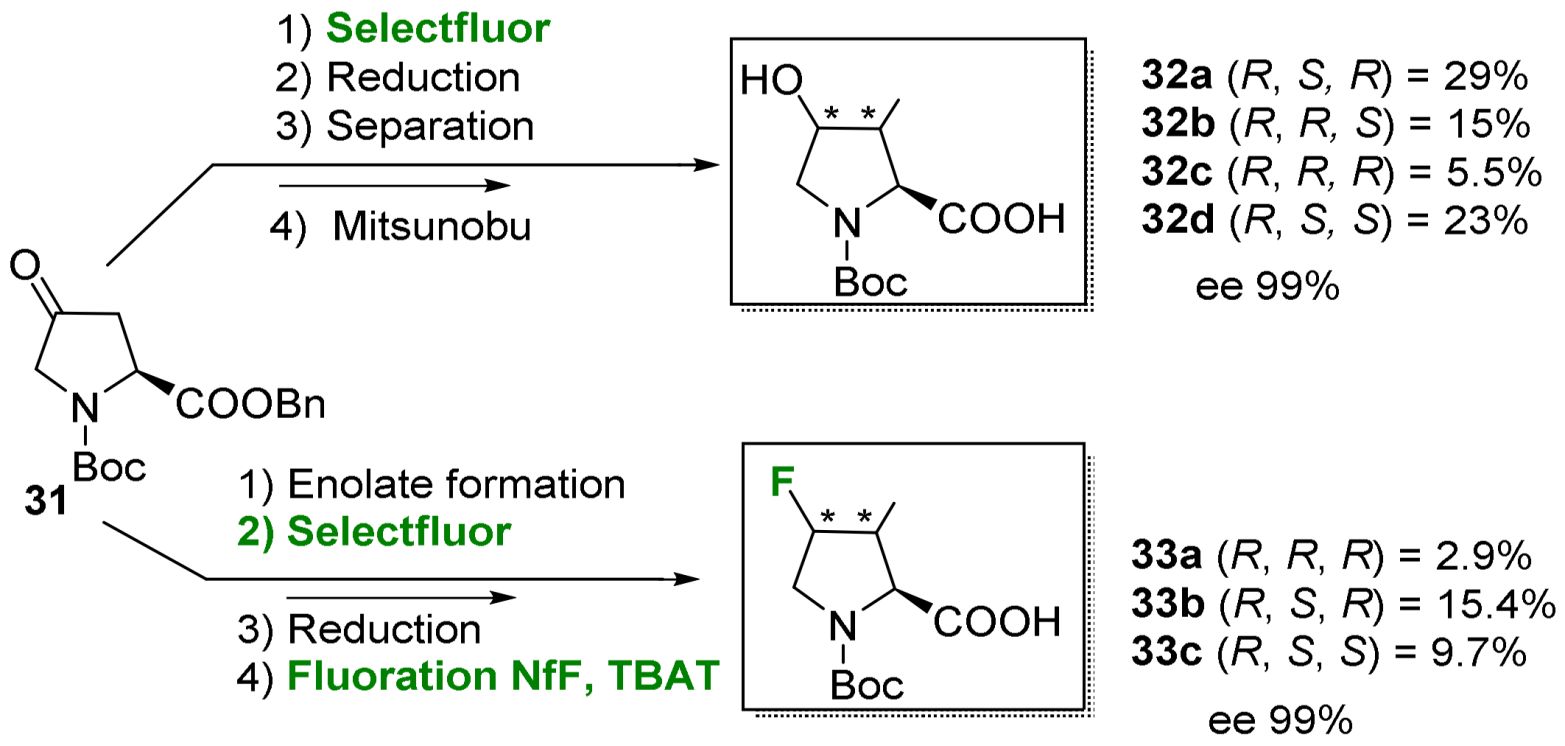
Synthesis of 3-fluoro-4-hydroxyproline and 3,4-difluoroproline [25,26,27].

Linclau’s group, in order to improve their synthesis of the *cis*-(3*R*,4*S)*-*N*-Boc-3,4-difluoroproline **33b [26]** and to have access to the *trans*-(3*S*,4*S*) and *trans*-(3*R*,4*R*) stereoisomers, used an alternative approach starting from the 4-oxoproline **31**. An electrophilic fluorination reaction using Selectfluor was tested via enolate formation. The reduction of the oxo group followed by the actions of NfF and TBAT gave the corresponding *trans* stereoisomers **33a** and **33c** (Scheme 7) [27].

Starting from 3-(*S*) or 3-(*R*)-hydroxyproline **34**, the Dugave group synthesized the two stereoisomers of-3-fluoroproline **36** via a direct fluorination reaction or configuration inversion by a Mitsunobu reaction, followed by a fluorination step [28].

Raines et al., inspired by the work of the Dugave group, started from the 3-(*S*)-hydroxyproline **34**. After a Mitsunobu reaction and a morph-Dast-mediated fluorination, the enantiopure 3-(*R*)-fluoroproline **37** was obtained [29].

**Scheme 8:**
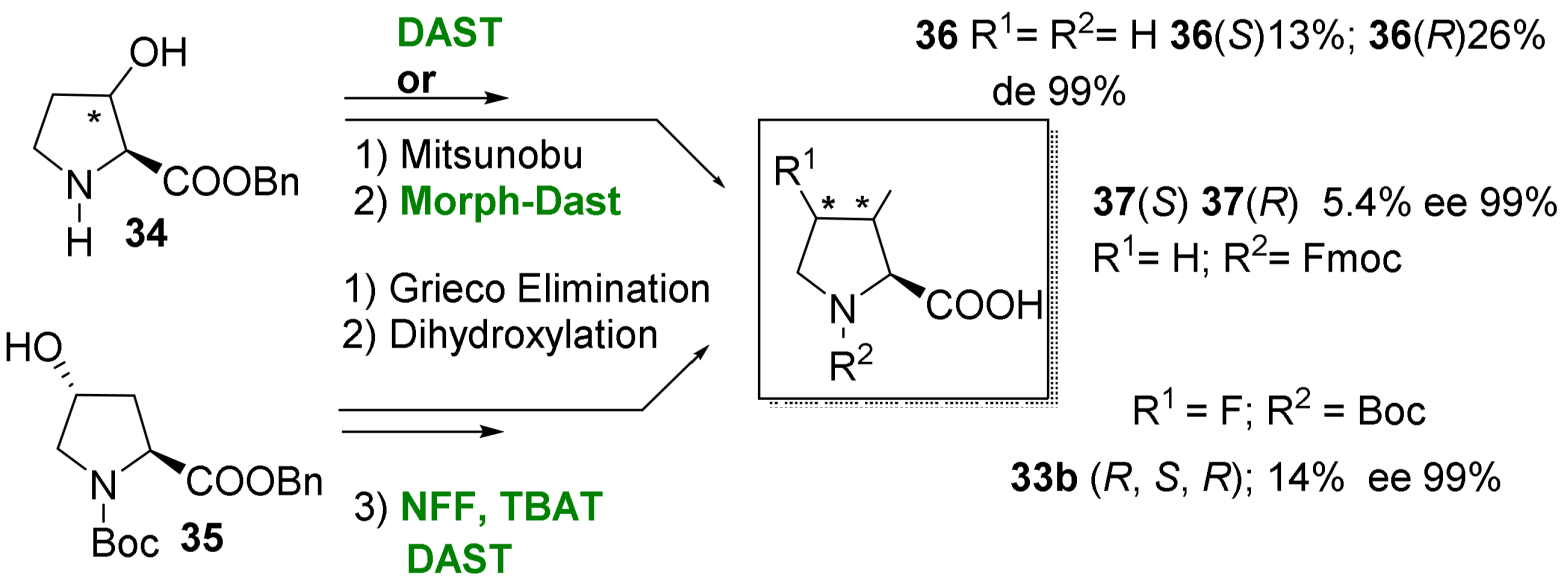
Synthesis of 3-fluoroproline and 3,4-difluoroproline [26,27,28,29].

Linclau et al. synthesized the vicinal *cis*-(3*R*,4*S)*-*N*-Boc-3, 4-difluoroproline **33b** from the 4-hydroxyproline **35**. A two-step elimination reaction followed by a stereoselective dihydroxylation and difluorination reaction using the NFF reagent gave the fluorinated proline **33b** [26]. Based on an optimized procedure using DAST reagent instead of NFF, TBAT was reported by the same group one year later This reaction was applied to the Fmoc- or Boc-protected hydroxyproline and gave the expected (3*S*,4*R*)-stereoisomer in better yield and regioselectivity and with lower racemization [27] (Scheme 8).

**Scheme 9:**
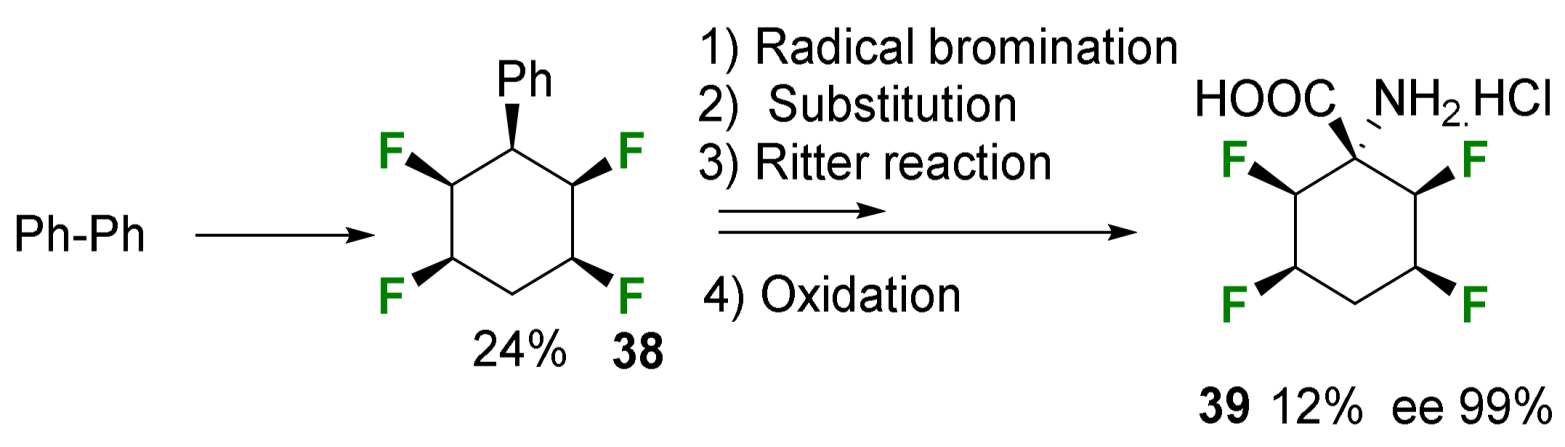
Synthesis of 1-amino-2,3,5,6-tetrafluorocyclohexylmethanoic acid [30,31].

O’Hagan et al. synthesized the all-*cis*-2,3,5,6-tetrafluorocyclohexane **38** from biphenyl [30]. A sequence radical bromination–substitution–Ritter reaction–oxidation gave **39** in enantiopure form [31] (Scheme 9).

### 2.2. α-Difluoroalkyl-α-Amino Acids

**Figure 1:**
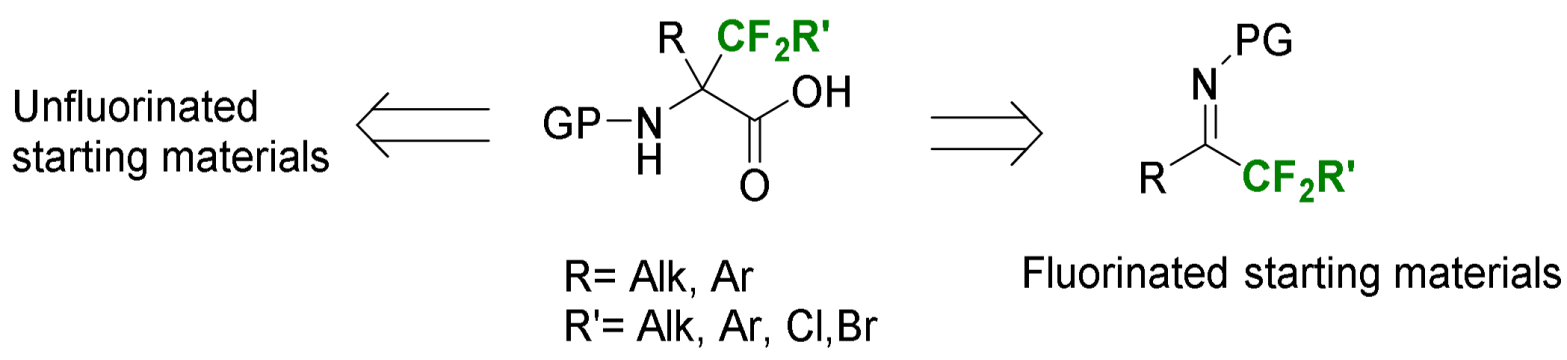
General retrosynthetic pathway for the synthesis of α-difluoroalkyl-α-amino acids.

In this section, methods utilizing non-fluorinated starting materials will be presented first. The key step generally involved nucleophilic fluorination. In the second part, the syntheses from fluorinated starting materials will be presented using catalytic or chiral auxiliary-based methods (Figure 1).

#### 2.2.1. Fluorination of a Chiral Precursor as the Key Step

**Scheme 10:**
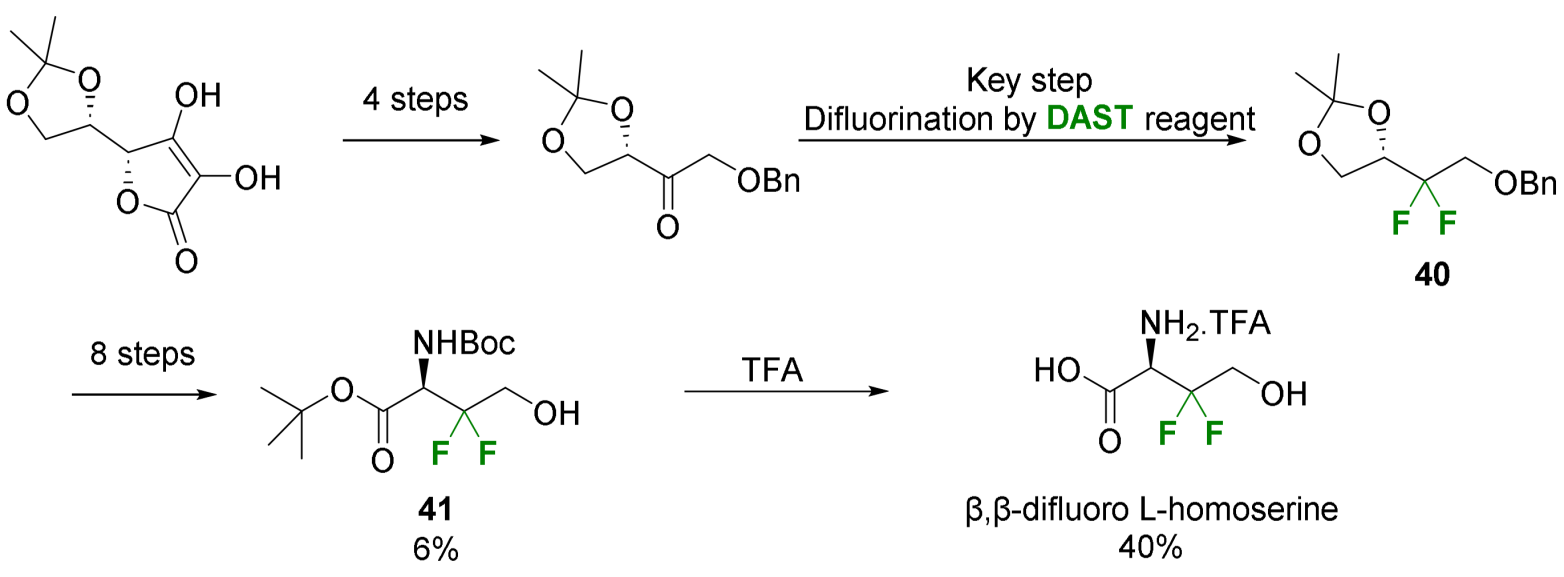
Synthesis of β,β-difluoro-l-homoserine from ascorbic acid [32].

In 1998, Liu et al. developed a strategy to synthesize β,β-difluoro-substituted l-homocysteine, l-homoserine, and l-methionine [32]. The strategy is based on the synthesis of (2*S*)-β,β-difluoro-2-hydroxy-butan-1,4-diol starting from l-isoascorbic acid. The synthesis began with oxidative cleavage followed by the reduction of the ester into an alcohol, which was then selectively benzylated. Swern oxidation of the secondary alcohol, followed by difluorination with DAST, yielded the difluorinated compound **40**. The protected β,β-difluoro-l-homoserine **41** was obtained in a 6% overall yield after 12 steps. A final step using TFA provided the fully deprotected β,β-difluoro-l-homoserine in a 40% yield (Scheme 10).

**Scheme 11:**
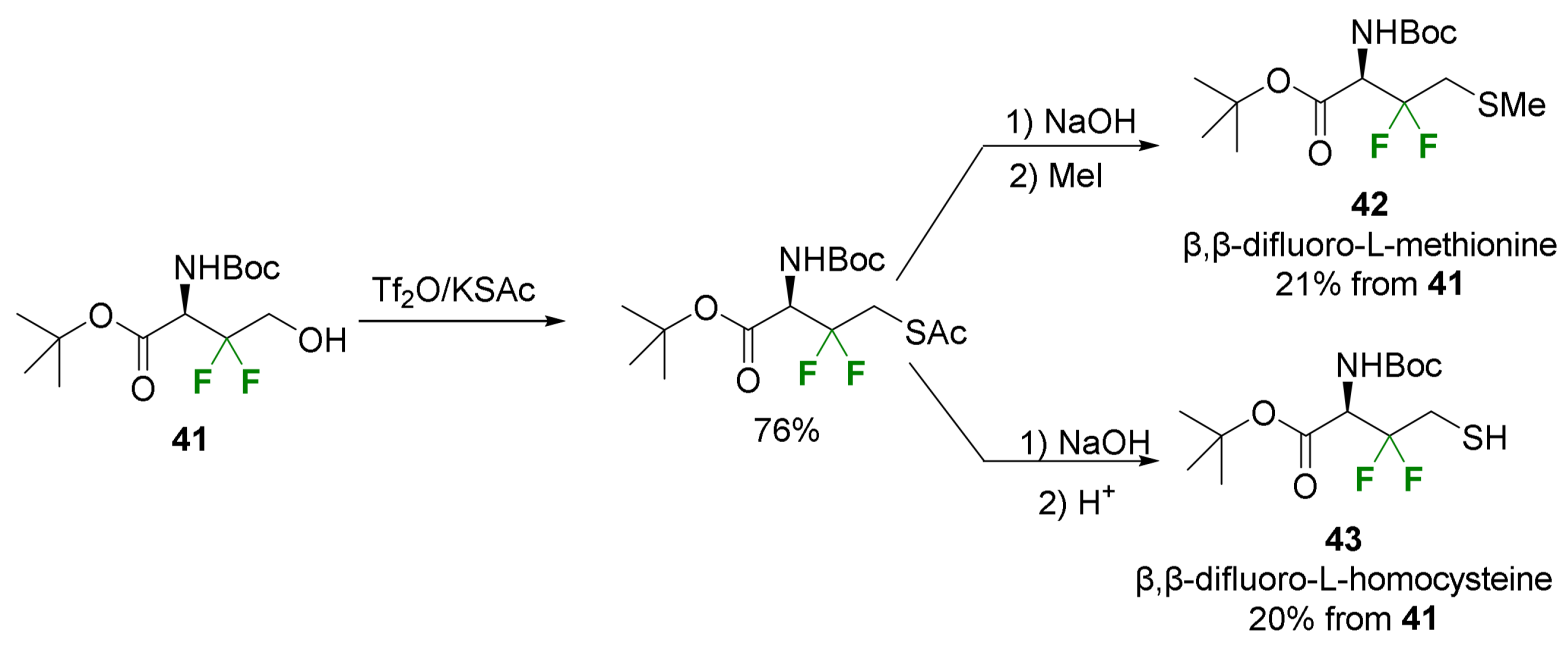
Synthesis of β,β-difluoro-l-methionine and β,β-difluoro-l-homocysteine from **41 [32]**.

In a similar manner, compound **41** was transformed into the β,β-difluoro-l-methionine **42** and β,β-difluoro-l-homocysteine **43** (Scheme 11).

**Scheme 12:**
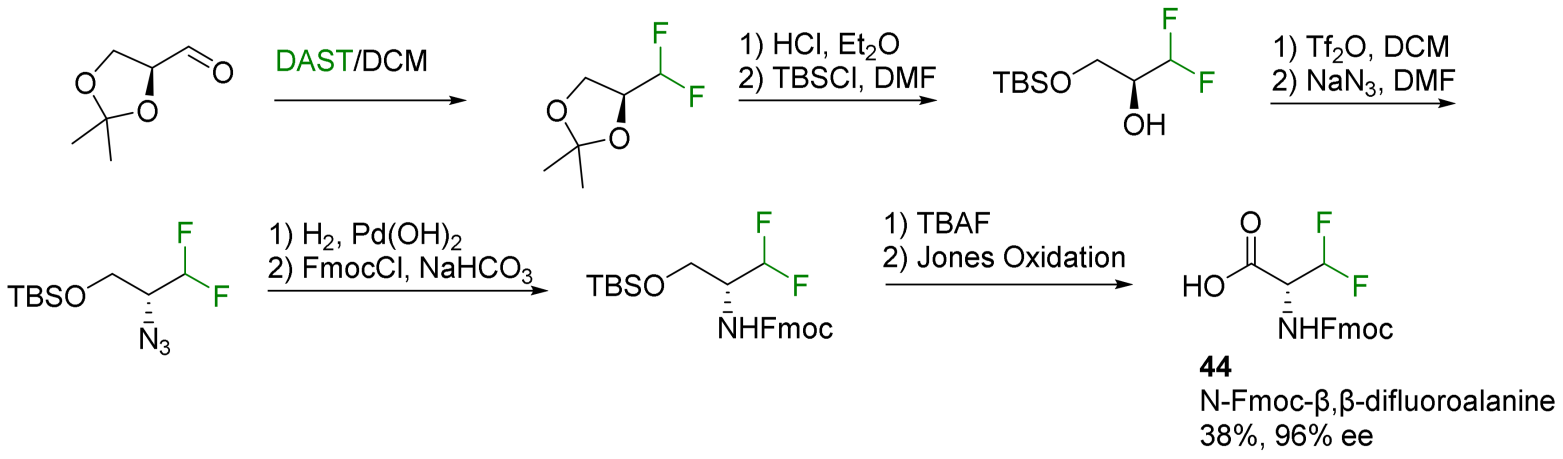
Synthesis of *N*-Fmoc-β,β-difluoroalanine from l-glyceraldehyde [33].

In 2007, van der Donk et al. proposed a nine-step synthesis of enantiopure Fmoc-difluoroalanine from ascorbic acid [33]. The key step consisted of the fluorination of *l*-glyceraldehyde acetonide using DAST. The Fmoc-β,β-difluoroalanine **44** was obtained after several steps, as depicted in Scheme 12.

**Scheme 13:**
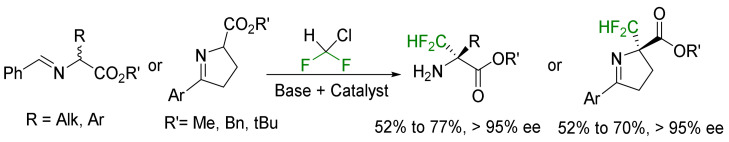
Asymmetric difluoromethylation from difluorocarbene [34].

In 2021, Guo et al. reported the synthesis of chiral α-difluorinated amino acids with excellent ee via copper-catalyzed asymmetric difluoromethylation using a difluorocarbene generated in situ from difluorochloromethane under basic conditions (Scheme 13) [34].

#### 2.2.2. Syntheses from Fluorinated Starting Materials

- Catalytic methods

**Scheme 14:**
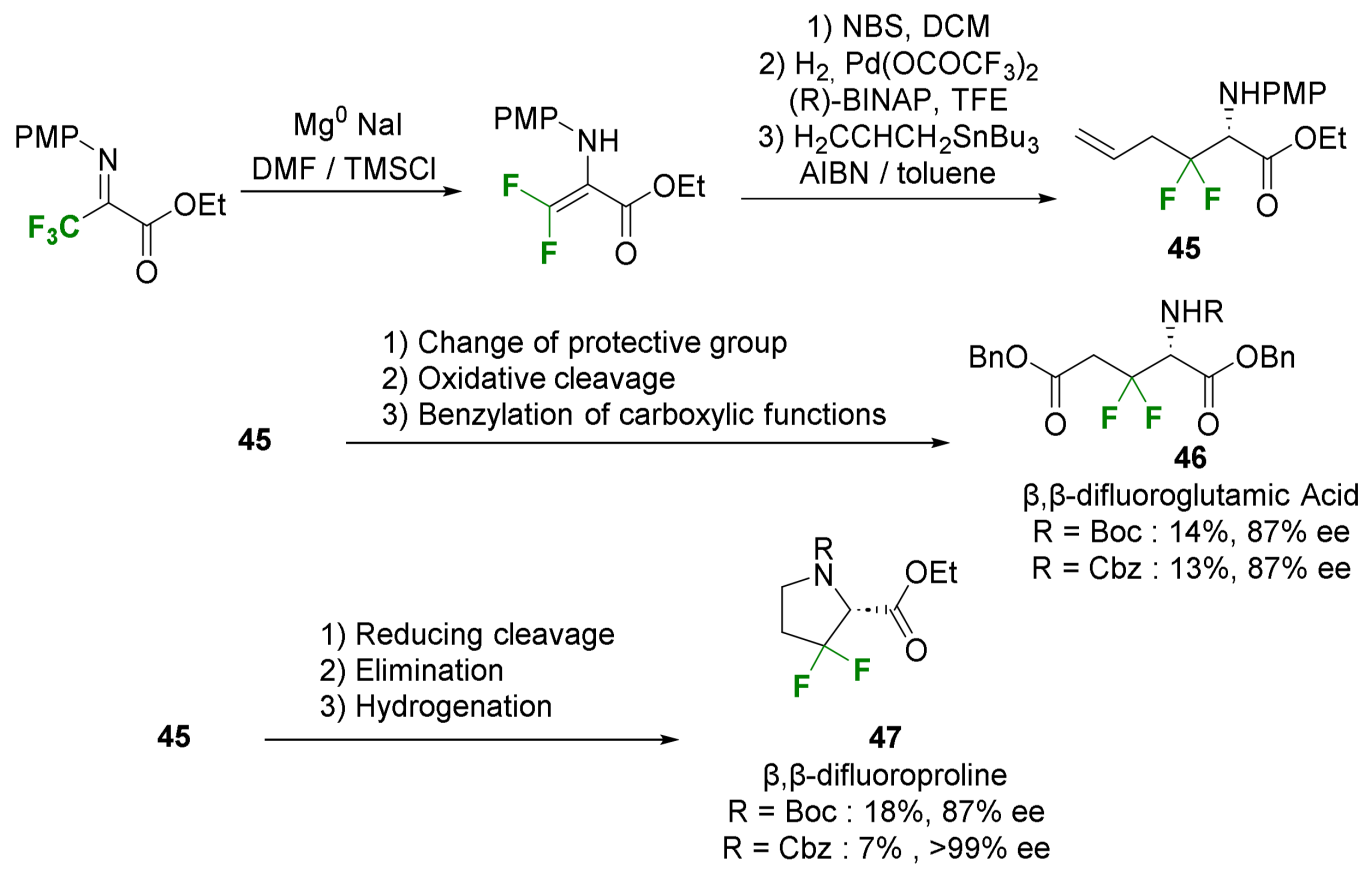
Synthesis of β,β-difluoroglutamic acid and β,β-difluoroproline derivatives [35].

In 2004, Uneyama et al. reported the synthesis of β,β-difluoroglutamic acid and β,β-difluoroproline [35]. The synthesis of β,β-difluoroglutamic acid began with a defluorination reaction of the starting reagent with magnesium and trimethylsilyl chloride to form the difluoro enaminoester (Scheme 14). This enaminoester has been synthesized on a large scale. The enaminoester was then reacted with NBS to form the bromodifluoromethyl iminoester in excellent yield. The special feature of this synthesis is the use of asymmetric reduction, enabling the iminoester to be reduced to the amine with excellent enantiomeric excess. The freshly reduced product underwent a radical allylation and the product **45** was formed in good yield with a high ee. The derivative **45** could be used to form either β,β-difluoroglutamic acid or β,β-difluoroproline derivatives. Changing the protecting group of **45** with a Cbz group allowed the enrichment of the ee up to 99% by recrystallization. The difluoroglutamic benzyl ester **46** was produced by the oxidative cleavage of **45** with RuO_2_/NaIO_4_. Reductive ozonolysis of **45** followed by cyclization afforded the β,β-difluoroproline **47**.

**Scheme 15:**
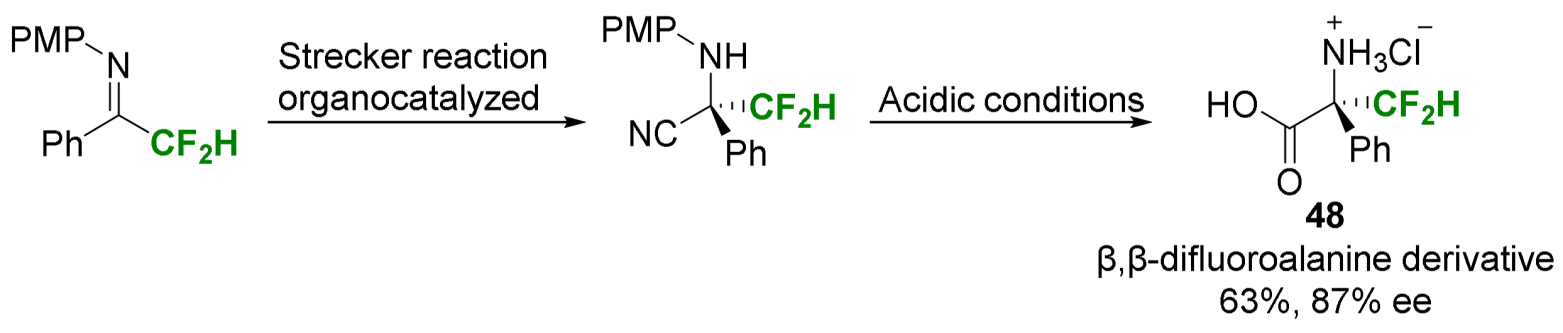
β,β-Difluoroalanine derivative produced by an enantioselective Strecker reaction [36].

In 2011, the team of Zhou et al. synthesized a β,β-difluoroalanine derivative by an enantioselective Strecker reaction [36]. In the CF_2_H series, the best conditions gave the target compound in a 94% yield and 92% ee. Once the product was synthesized, amine deprotection and nitrile hydrolysis yielded the fully deprotected amino acid **48** in a 63% yield (Scheme 15).

**Scheme 16:**
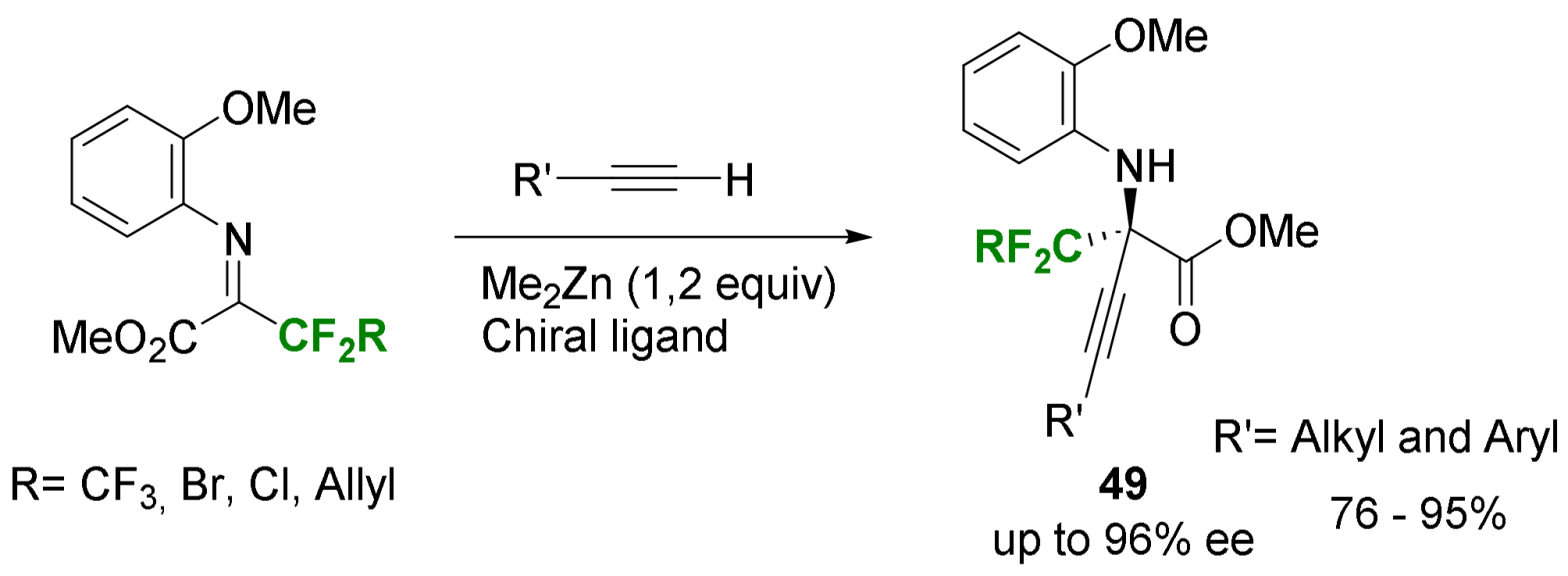
Enantioselective alkynylation of ketoimine [37].

In 2013, a paper by Zhang’s team reported a method for the enantioselective alkynylation of ketoimine catalyzed by a zinc/BINOL complex [37]. Several different substrates were used, each of which yielded the desired product **49** in very good yields (76–95%), with up to 96% ee (Scheme 16).

**Scheme 17:**
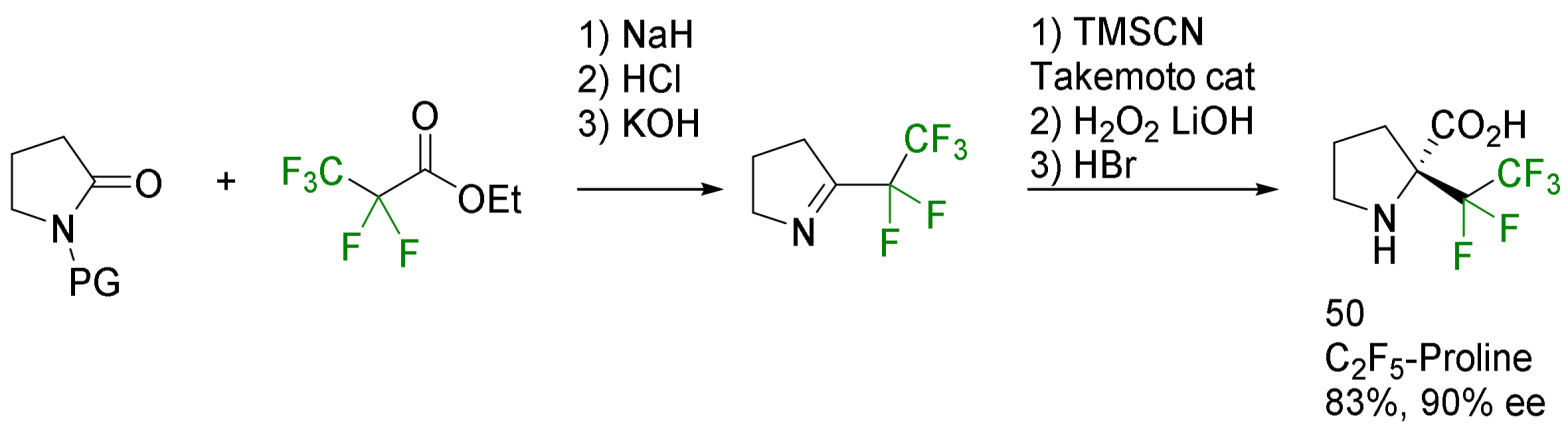
Synthesis of perfluoroalkyl α-prolines [38].

In 2018, Nenajdenko et al. proposed an enantioselective synthesis of cyclic amino acids. With this four-step method, α-perfluoroalkylated prolines as well as six- and seven-ring derivatives were synthesized [38]. The first step consisted of forming cyclic ketimines containing either a CF_3_ or a C_2_F_5_ group. Enantioselective addition of the nitrile group using Takemoto catalyst gave the corresponding amino nitrile. Further hydrolysis afforded the C_2_F_5_-proline **50** in an 83% overall yield and 90% ee (Scheme 17).

- Chiral auxiliary-based methods

**Scheme 18:**
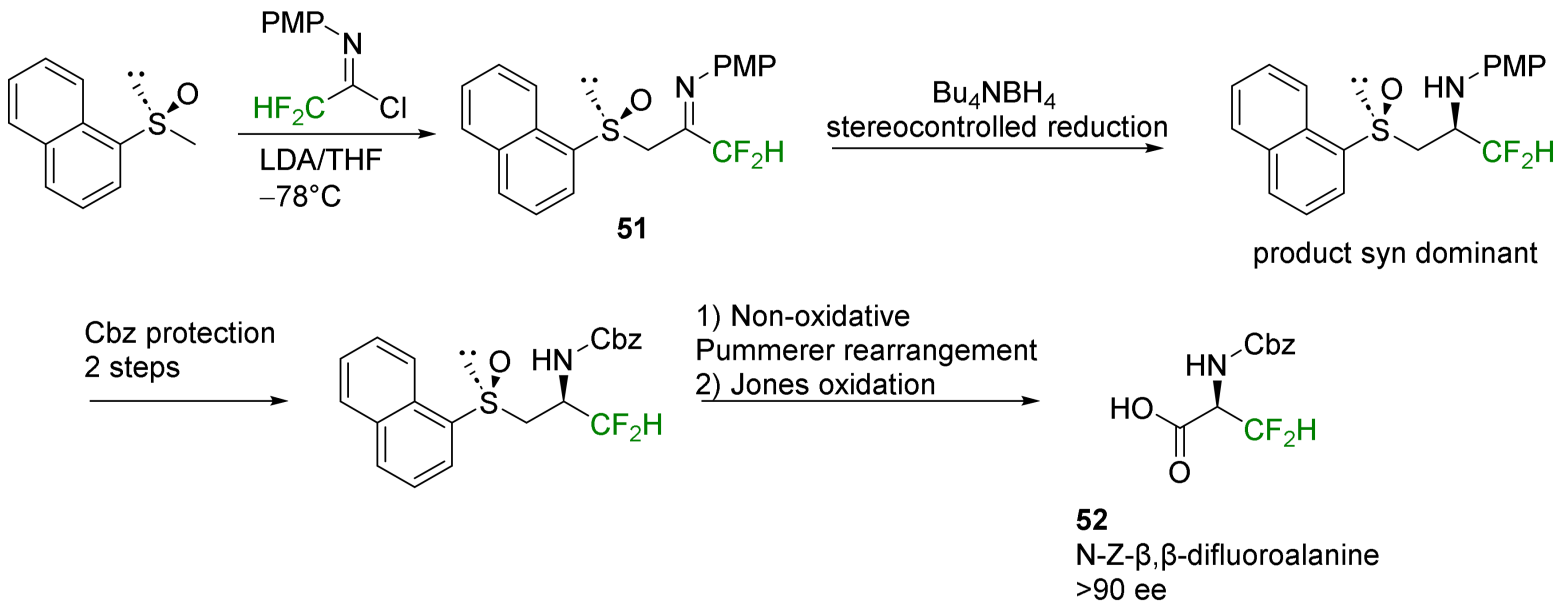
Synthesis of *N*-Z-β,β-difluoroalanine by stereocontrolled reduction as a key step [39].

In 2001, Fustero et al. developed a method for the enantioselective formation of β,β-difluoroalanine protected by a Cbz group on the nitrogen [39]. The strategy involved a stereocontrolled reduction through intramolecular π-stacking effects. The key step of this synthesis was the formation of a fluorinated β-sulfinylimine **51** from a commercial fluorinated imine and a sulfoxide. This imine was reduced by *tetra*-*n*-butylammonium borohydride. Once reduced to an amine, the PMP group was removed to add Cbz as a protecting group. The sulfoxide group was removed through a non-oxidative Pummerer rearrangement, and the resulting alcohol was then oxidized to give the protected β,β-difluoroalanine **52** in good yields with ee > 90% (Scheme 18).

**Scheme 19:**
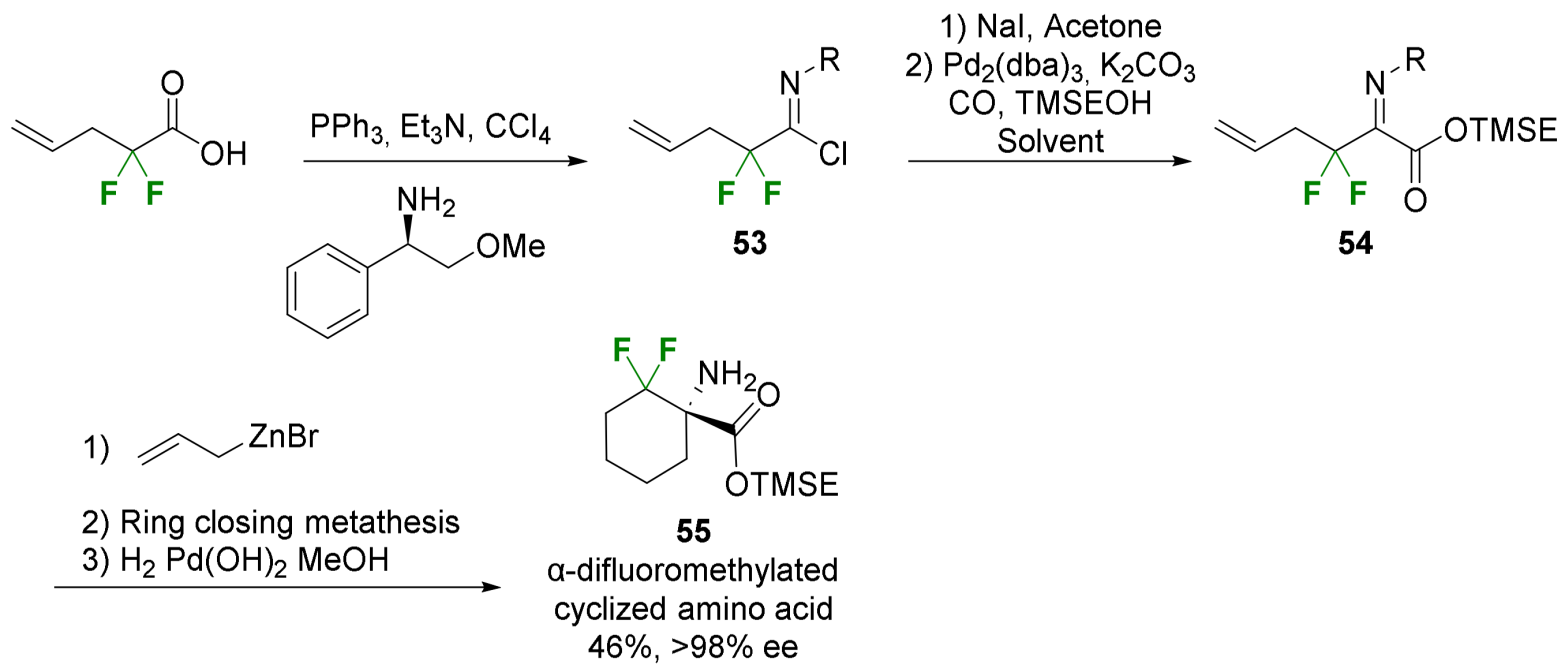
Synthesis of an α-difluoromethylated cyclized amino acid by a ring closing metathesis method [40].

In 2006, Fustero’s team proposed the synthesis of a new difluoromethylated cyclic amino acid derivative [40]. The synthesis began with the preparation of the iminoester **54** obtained from the imidoyl chloride **53**. Stereoselective addition of an organozinc to the iminoester followed by a ring closing metathesis reaction and hydrogenolysis formed the cyclized α-difluoromethylated amino acid **55** (Scheme 19).

**Scheme 20:**
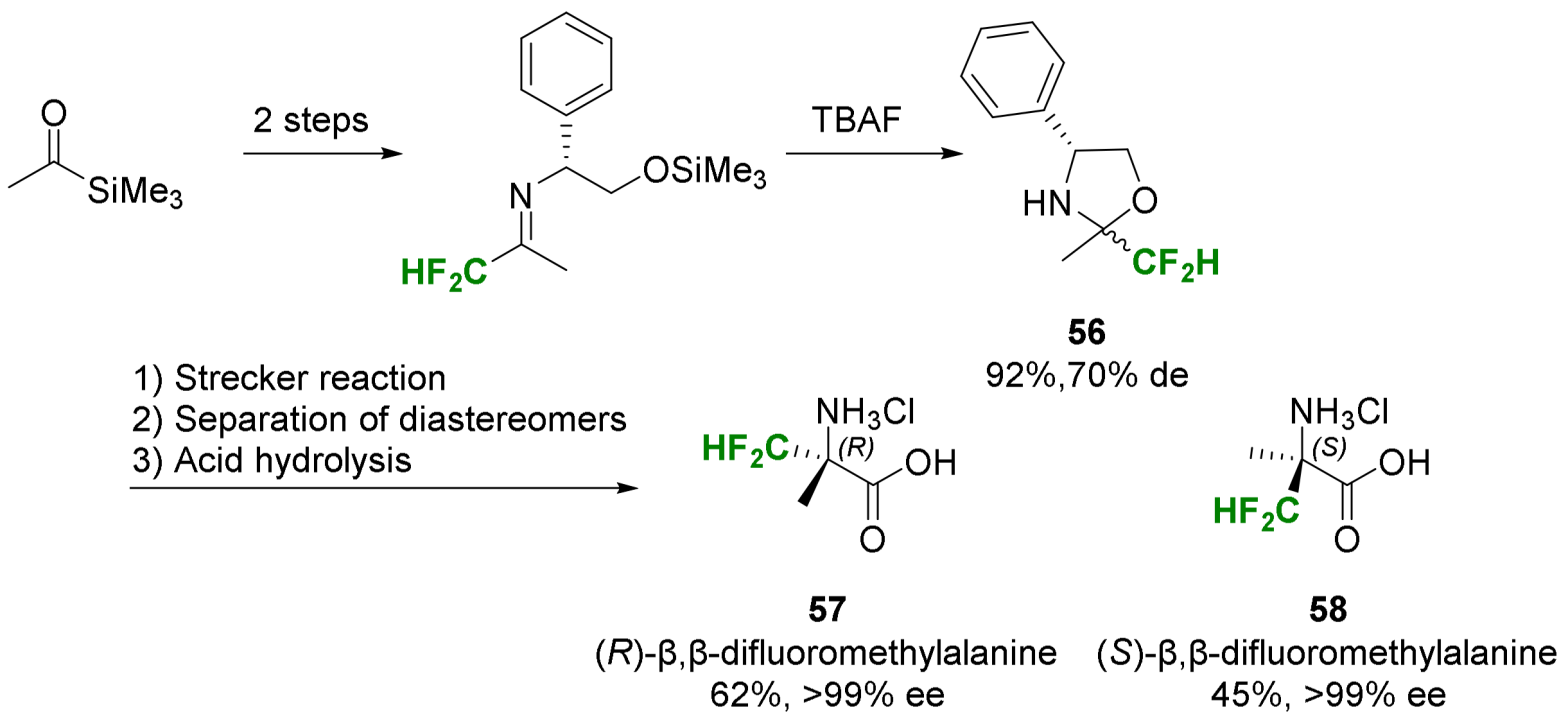
Synthesis of enantiopure (*R*)- and (*S*)-β,β-difluoromethylalanine [41].

In 2008, Portella et al. proposed the synthesis of both enantiomers of β,β-difluoromethylalanine through a Strecker reaction on the difluoromethylated oxazolidine **56** [41]. After separation of both nitrile diastereomers, an acidic hydrolysis reaction gave enantiopure (*R*)-β,β-difluoromethylalanine **57** and (*S*)-β,β-difluoromethylalanine **58** (Scheme 20).

**Scheme 21:**
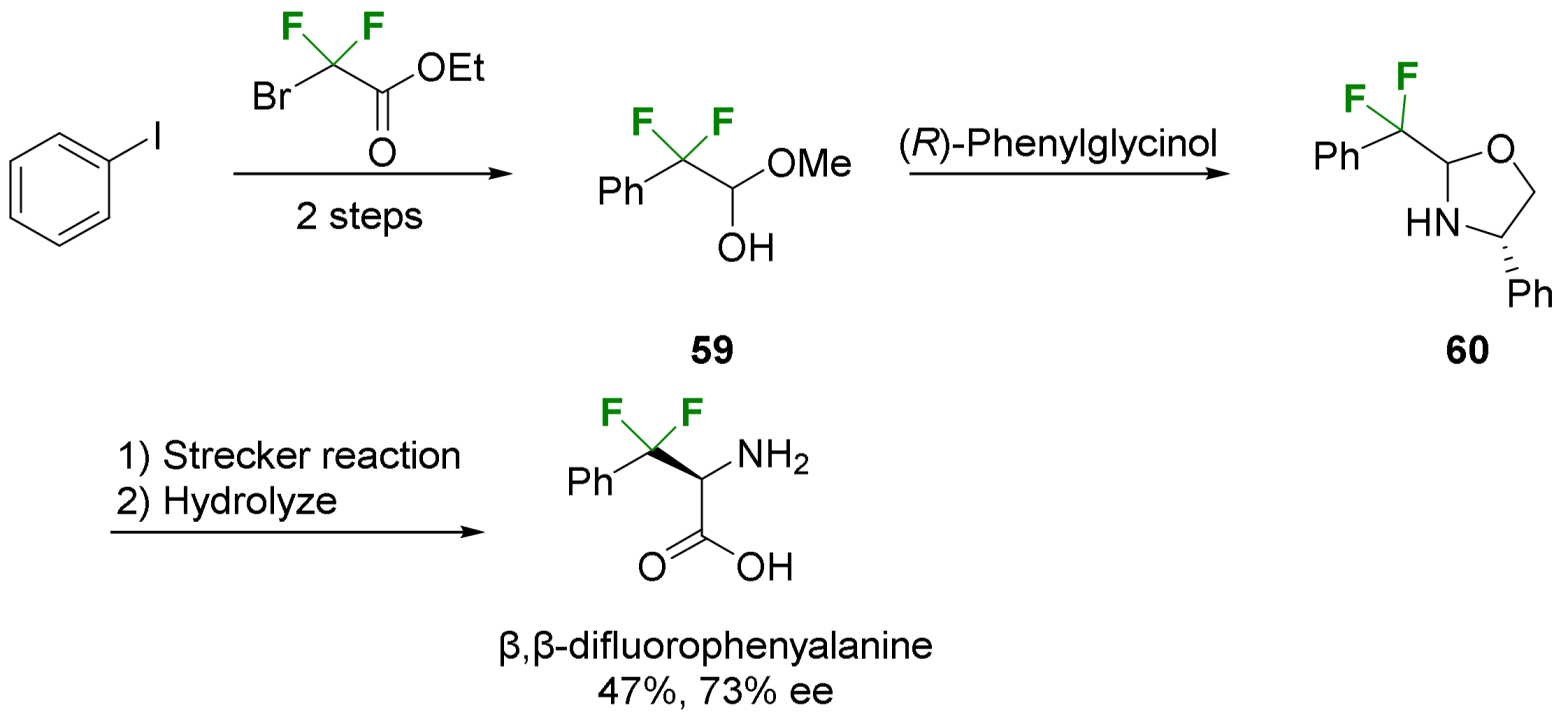
Synthesis of enantioenriched β,β-difluorophenylalanine [42].

Liu’s team has developed the synthesis of enantioenriched difluorophenylalanine using the hemiaminal **59**, obtained from a cross coupling between ethyl bromodifluoroacetate and phenyl iodide [42]. The hemiaminal was then reacted with (*R*)-phenylglycinol to give oxazolidine **60**. After a Strecker reaction, the nitrile group was hydrolyzed and the amino acid was obtained in a 47% yield and 73% ee (Scheme 21).

**Scheme 22:**
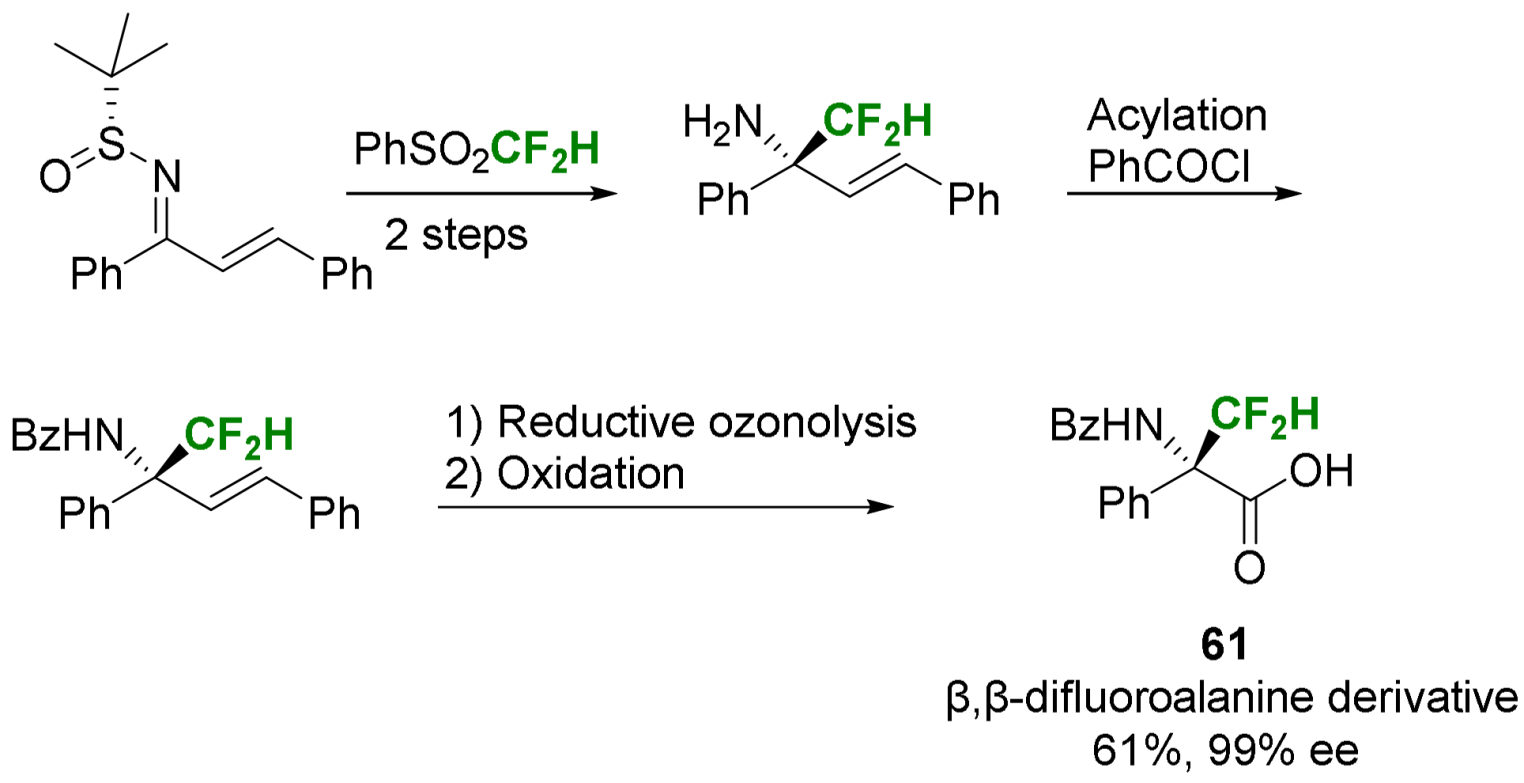
Synthesis of the β,β-difluoroalanine derivative [43].

In 2010, Hu’s team published a method for efficiently synthesizing α-difluoromethylamine from *N*-*tert*-butylsulfinyl ketimine and difluoromethyl phenylsulfone [43]. To get the amino acid, the protected allylic amine was subjected to reductive ozonolysis and subsequent oxidation to the carboxylic acid, and the *N*-CBz-amino acid **61** was formed in a 61% overall yield with an excellent 99% ee (Scheme 22).

**Scheme 23:**
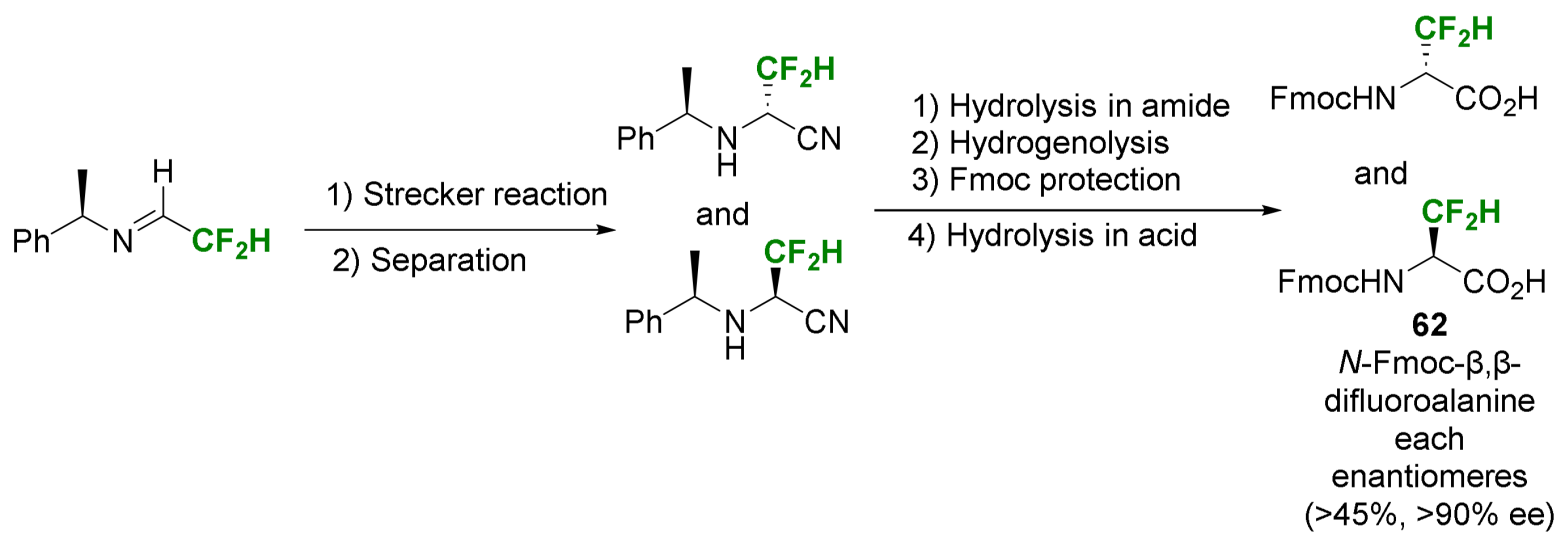
Synthesis of *N*-Fmoc-β,β-difluoroalanine by the Strecker reaction [44].

In 2023, Brigaud, Crousse et al. [44] reported the synthesis of enantioenriched (>90% ee) *N*-Fmoc-β,β-difluoroalanine from a chiral difluorinated aldimine employing Strecker’s strategy. The Strecker reaction provided two diastereomeric amino nitriles that were separated. Subsequent selective hydrolysis reactions yielded the final two difluoroalanine enantiomers in a good overall yield with an enantiomeric excess exceeding 90% (Scheme 23).

### 2.3. α-CF_3_-α-Amino Acids

**Figure 2:**
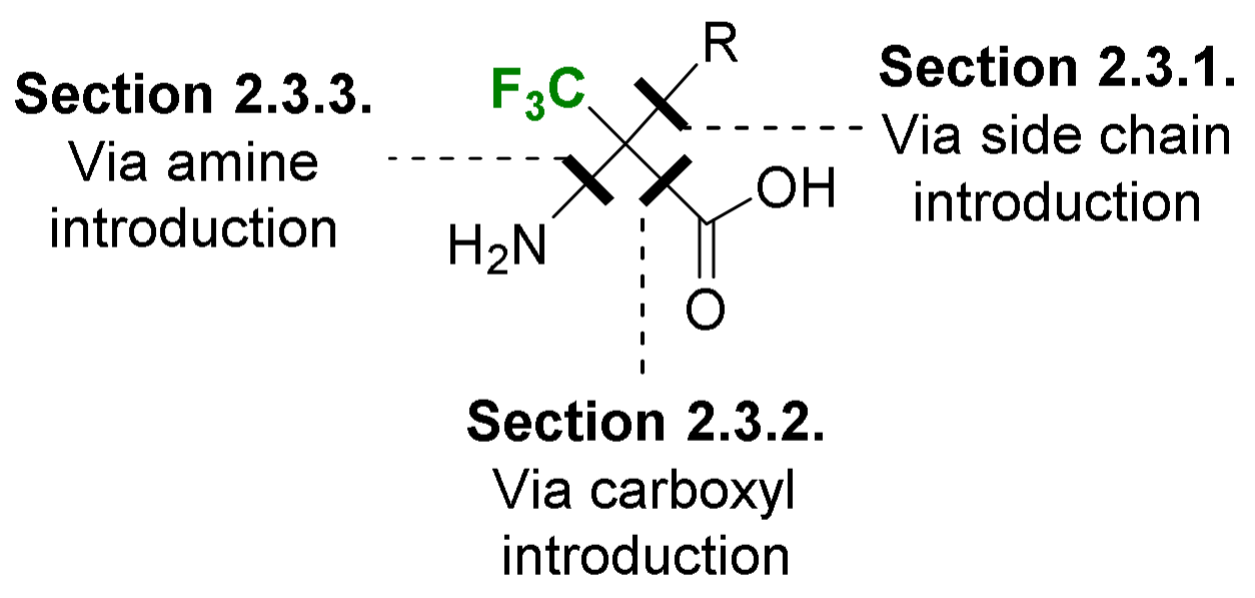
Retrosynthetic approach to α-trifluoromethyl α-amino acids.

This section will review the main reported strategies towards the asymmetric synthesis of tertiary and quaternary amino acids bearing a trifluoromethyl group at the alpha position. The literature published prior to 2011 on this topic has been exhaustively reviewed in the past and will not be mentioned in this section [4,45]. However, the past decade has seen many novel contributions to the field using modern synthetic methodologies to achieve a high degree of stereoselectivity. Only synthetic strategies leading to non-racemic α-CF_3_-α-amino acids able to be further used in peptide synthesis (i.e., either unprotected or protected with usual protecting groups) will be mentioned in the following section. The three retrosynthetic disconnections presented in Figure 2 will be reviewed in this section, focusing on the stereochemical aspect. The direct introduction of a trifluoromethyl group (fourth disconnection) is a process that proved very difficult to achieve. The work of Fustero and coworkers on this subject using the Ruppert–Prakash reagent (TMSCF_3_) remains the only reported example of such an approach [46]. Reported in 2008, it is therefore out of the scope of this section.

The simplest method for synthesizing α-CF_3_-α-amino acids involves utilizing highly functionalized imines derived from trifluoro pyruvates, resulting in the efficient production of various structurally diverse target compounds within a minimal number of steps. Nonetheless, strategies involving the introduction of the carboxyl moiety, mainly via Strecker-type reactions, or the amino group have also been the focus of important research efforts. Each route will be discussed in a separate sub-section on the following pages.

#### 2.3.1. Via the Introduction of the Side Chain

Considering that α-CF_3_-α-amino acids have as common features a carboxyl group, an amino group and a trifluoromethyl group linked to a single sp^3^ carbon, the most straightforward synthetic route to achieve molecular diversity is via late-stage introduction of the side chain. In the last decade, various novel stereoselective methodologies allowed the introduction of a large diversity of side chains onto trifluoromethyl imino or amino esters. This section proposes to review these synthetic achievements based on the types of side chains introduced.

- Alkynylation and allylation

Introduction of an alkyne or alkene group in the side chain of α-CF_3_-α-amino acids can be of very high interest, considering the large panel of further transformations offered by such a reactive moiety, as well as its straightforward incorporation into biomolecules.

The Osipov group has been dedicating much effort to the synthesis and applications of alkyne-containing molecular scaffolds. Although they are always working on racemic mixtures, they demonstrated the relevance of such functional groups on α-CF_3_-α-amino acids that thus motivated the development of stereoselective strategies [47,48,49,50,51,52,53,54].

**Scheme 24:**
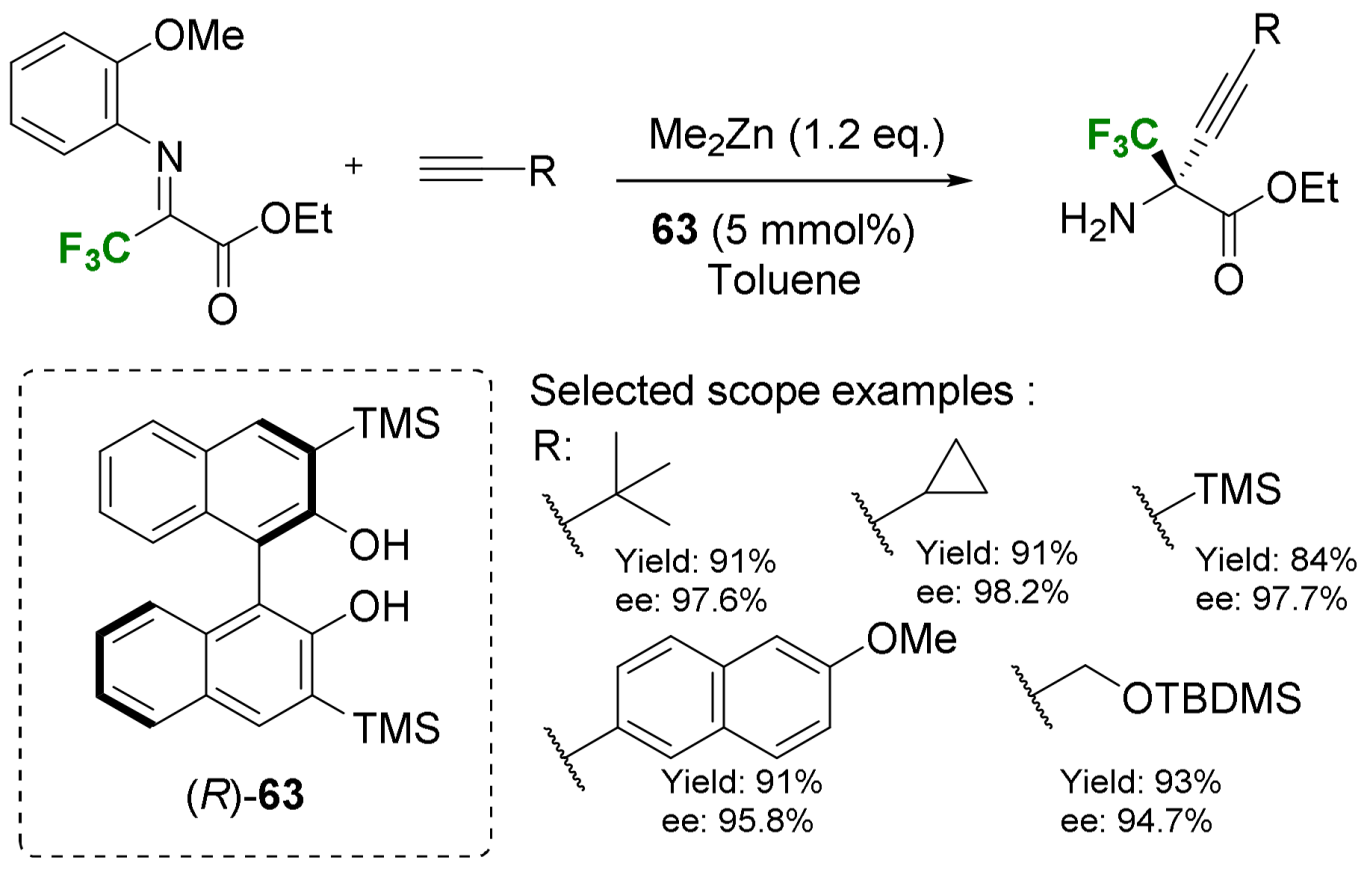
Zn/BINOL-catalyzed enantioselective alkynylation [55,56].

Zhang and colleagues disclosed the first catalytic enantioselective alkynylation involving α-CF_3_ ketoimine ester with zinc and BINOL **63 [55,56]**. This method allowed the utilization of various terminal alkynes, encompassing arynes, enynes, and alkynes, in asymmetric synthesis, yielding exceptional yields and high enantioselectivities (Scheme 24). Subsequent transformations of the carbon–carbon triple bond produced diverse α-CF_3_-α-amino acids, including α-CF_3_-proline, produced in a very efficient manner. Following this, Ma and colleagues reported a similar reaction using terminal 1,3-diynes [56], further expanding the diversity of side chains available via alkynylation.

**Scheme 25:**
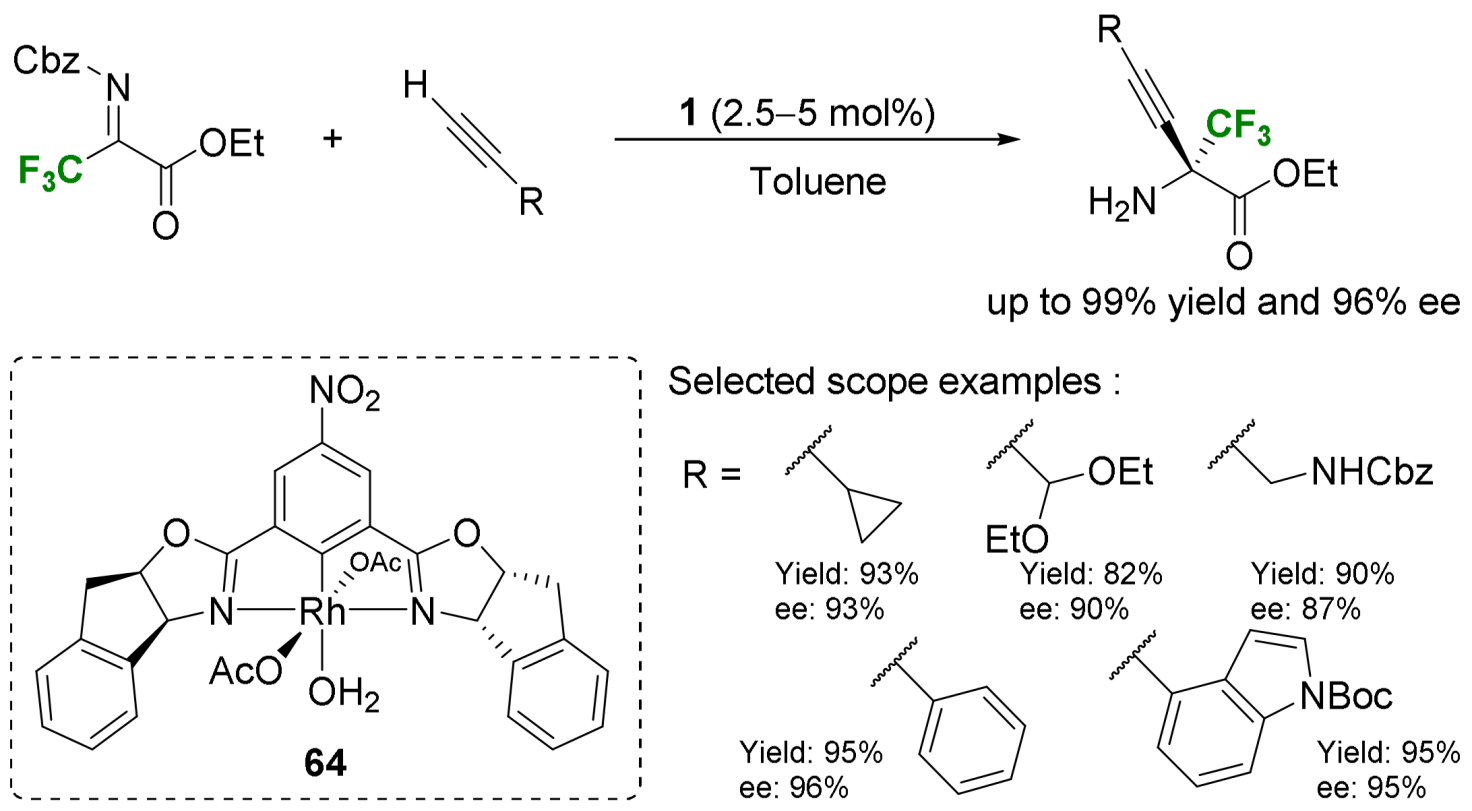
Rh-catalyzed enantioselective alkynylation of imino trifluoropyruvate [57,58].

In 2013, Ohshima and coworkers developed the Rh-catalyzed direct enantioselective alkynylation of *N*-protected imino trifluoropyruvate with terminal alkynes (Scheme 25) [57]. They achieved impressive yields and high enantioselectivities through careful adjustment of the *N*-protecting group and ligand on the Rh complexes. Both aryl- and alkyl-substituted terminal alkynes smoothly underwent this transformation at room temperature. However, lower enantioselectivities were observed when using alkyl-substituted terminal alkynes. Subsequently, they investigated the reaction mechanism and expanded the range of substrates applicable to this method [58].

**Scheme 26:**
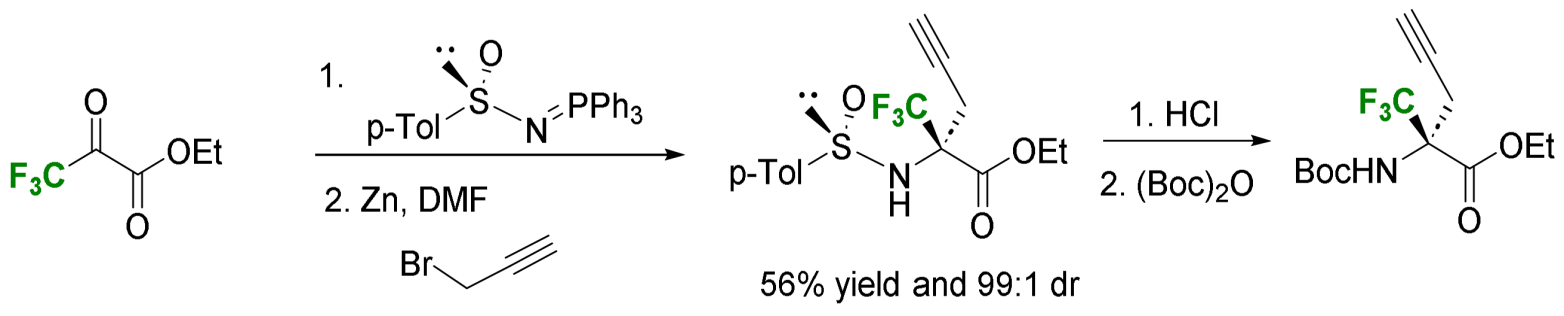
Zn-catalyzed diastereoselective propargylation [59,60].

Soon after, the Fustero group used a chiral arylsulfinyl imine to proceed to its highly diastereoselective zinc catalyzed propargylation in good yield without the need for a chiral ligand (Scheme 26). The chiral auxiliary was cleaved under acidic treatment and the resulting enantiopure amino acid was protected with a Boc group. They further studied the reactivity of this fluorinated propargyl amino ester upon gold catalyzed cycloadditions, resulting in the formation of original polycyclic fluorinated amino esters [59,60].

**Scheme 27:**
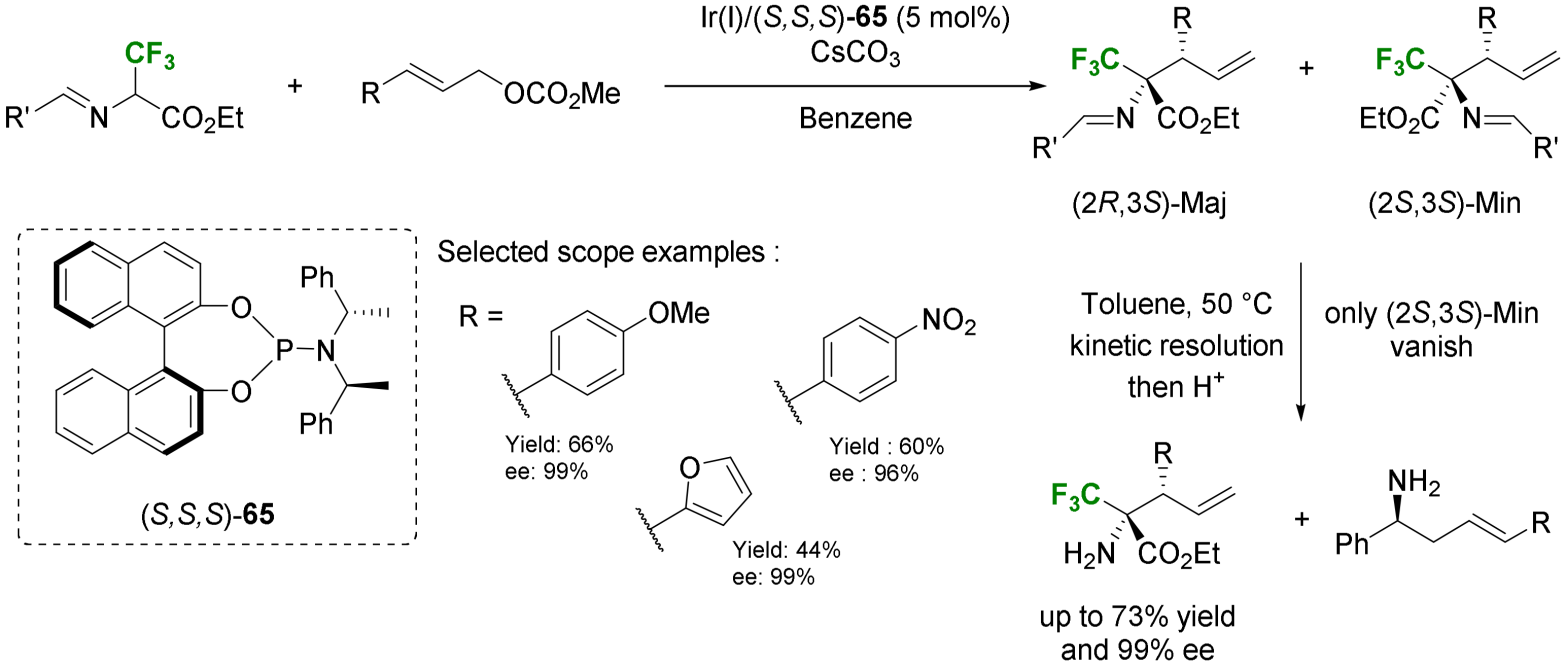
Ir-catalyzed electrophilic allylation of α-CF_3_-aldimine esters [61].

Recently, Wang, Chung, and their team introduced an innovative Ir-catalyzed allylation of α-trifluoromethyl aldimine esters followed by a kinetic resolution to produce optically active quaternary α-trifluoromethyl α-amino acids (Scheme 27) [61]. The use of chiral ligand **65** yielded two diastereomers of the allylated products with exceptional ee values. The team realized that a kinetic resolution occurred at 50 °C with the major diastereomer being left untouched while the minor one was completely subjected to a 2-aza-Cope rearrangement and converted to chiral homoallylic amines. This robust methodology offers a rather large scope of aromatic allylic substituents, preserving a high level of enantioselectivity.

**Scheme 28:**
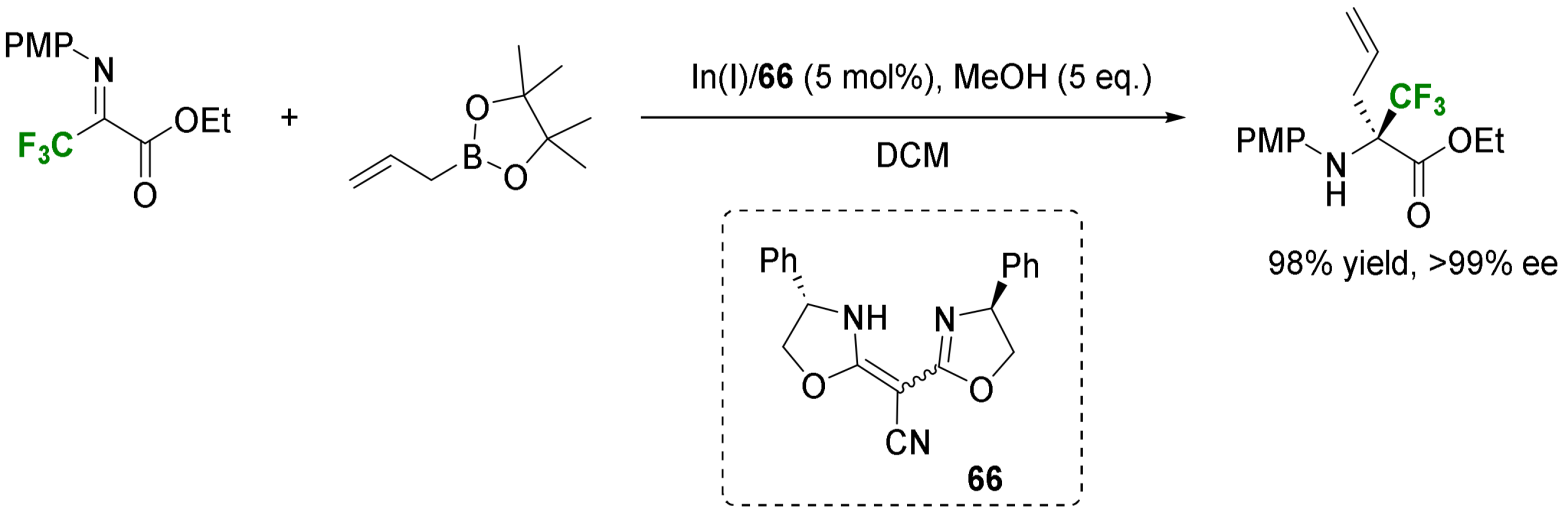
In-catalyzed asymmetric allylation [62].

Kürti and colleagues introduced a highly enantioselective method for the synthesis of optically active α-allyl-α-CF_3_ amino acid derivatives [62]. This involved the catalytic asymmetric allylation of α-trifluoromethyl acyclic α-imino esters with allylBpin (Scheme 28). The combination of indium iodide (InI) and Box-type ligand **66** with MeOH proved efficient, yielding the desired products in yields ranging from 91% to 98% and with enantiomeric excesses from 90% to 99%. Enantioenriched α-allyl-α-CF_3_-amino acid and the α-CF_3_ proline derivative were successfully prepared through a hydroboration–oxidation–cyclisation synthetic sequence.

**Scheme 29:**
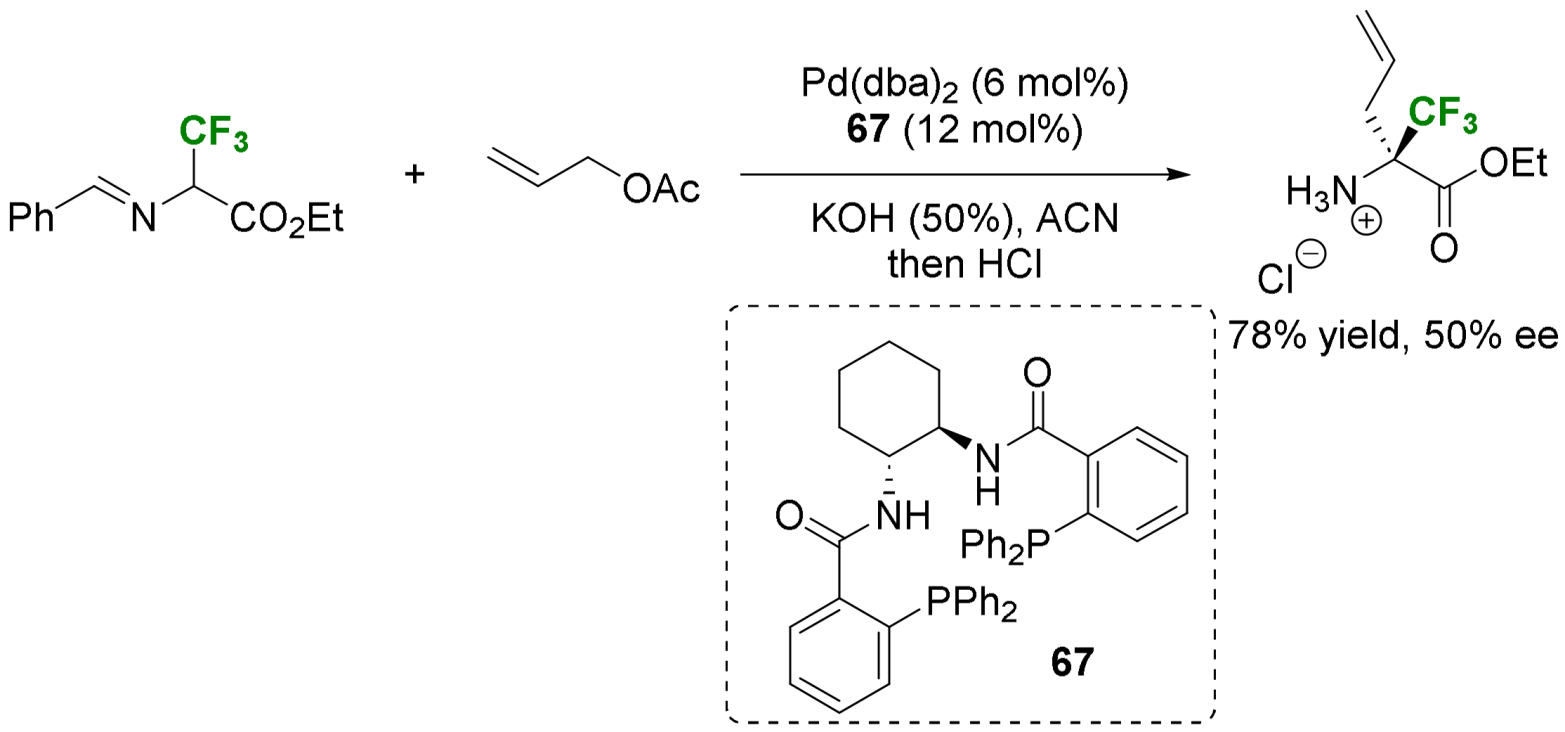
Enantioselective Pd-catalyzed allylation [63].

In 2019, Waser and his team proposed a straightforward synthesis of α-CF_3_-α-amino acid via a Pd-catalyzed α-allylation of α-trifluoromethyl aldimine esters with allylic acetate [63]. A broad scope of aromatic allylic substituents was tolerated, but only with the racemic version of the method. As proof of concept, they noted that employing 6 mol% of Pd(dba)_2_ ligated with Trost’s ligand allowed the production of the enantioenriched α-allyl-α-CF_3_-α-amino acid, achieving a 78% yield with 50% ee after acidic hydrolysis (Scheme 29).

- α-Arylation: the Friedel–Crafts reaction

**Scheme 30:**
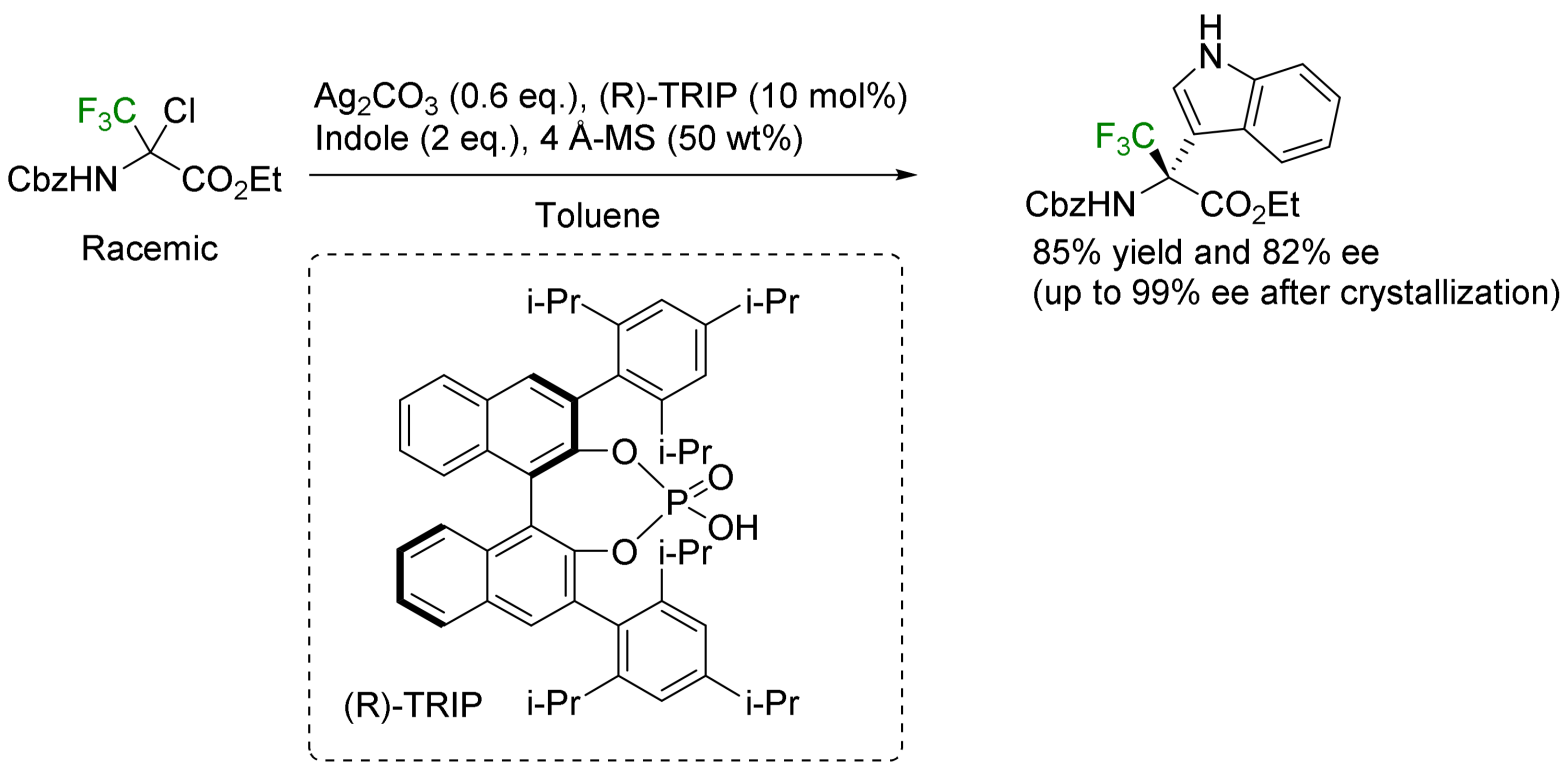
Chiral phosphoric acid-catalyzed Friedel–Crafts reaction of indole with racemic α-Cl-α-CF_3_-amino acid [64].

In 2020, Roche and colleagues reported an aza-Friedel–Crafts reaction involving electron-rich (hetero)arenes and in situ generated α-CF_3_ ketoimine from α-Cl-amino ester racemate [64]. This straightforward alkylation process offers mild conditions, high efficiency, and a wide range of aromatic nucleophiles, although only one example was applied to the enantioselective version of the methodology. This last one was achieved using the chiral phosphoric acid (*R*)-TRIP as an organocatalyst, allowing the isolation of the enantiopure amino acid after crystallization (Scheme 30).

**Scheme 31:**
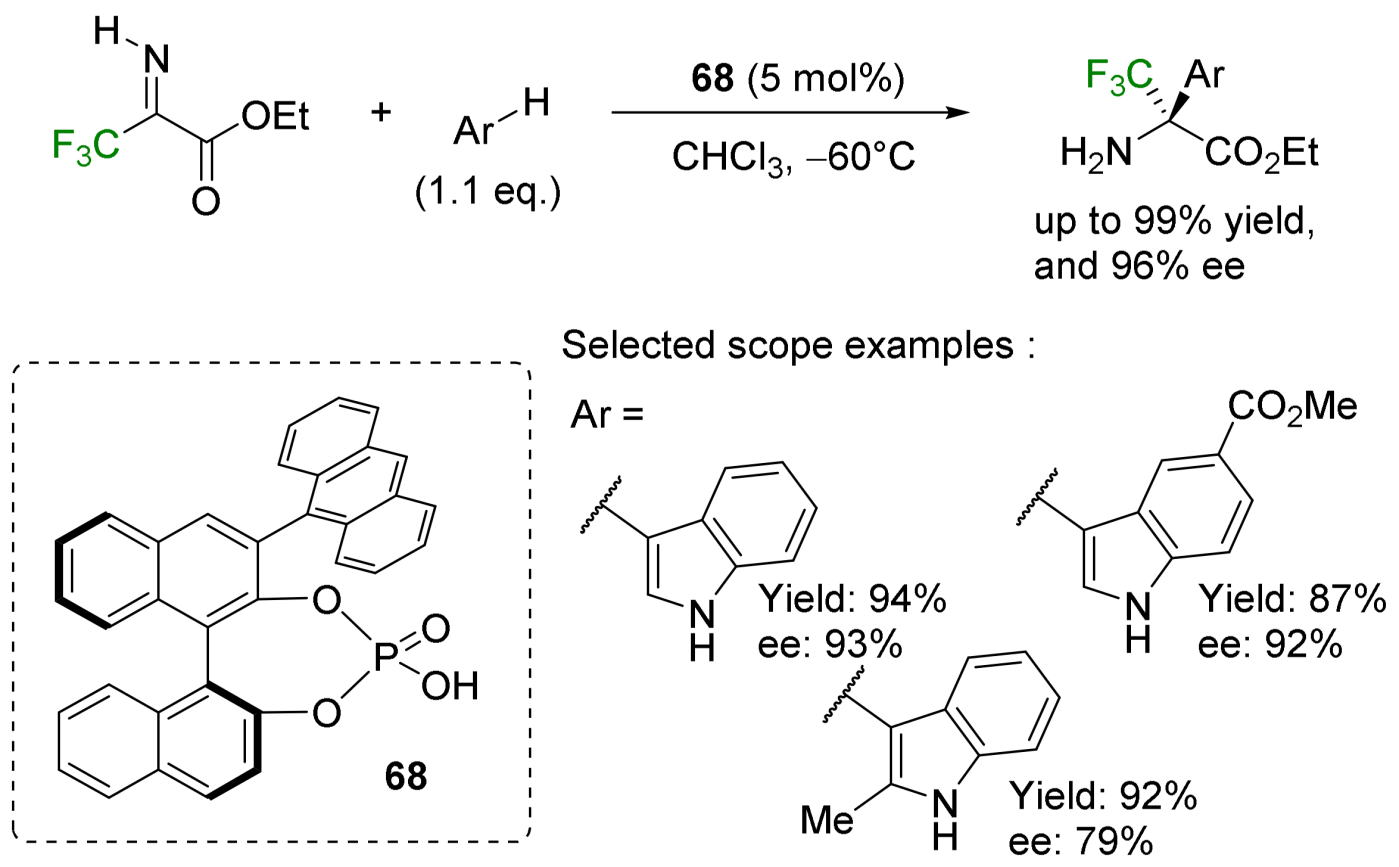
Monosubstituted chiral phosphoric acid as an organocatalyst in a Friedel–Crafts alkylation [65].

A similar reactivity was proposed by Ohshima and coworkers who developed a direct Friedel–Crafts alkylation reaction on unprotected trifluoromethylated imino esters [65]. Interestingly, optimization of the methodology revealed that the best results in terms of yield and stereoselectivity were obtained using the unsymmetrical chiral monosubstituted phosphoric acid **68** (Scheme 31). This novel methodology tolerated a rather broad scope of aromatic substrates, although they were limited to diversely substituted indoles and pyrroles. In each case, the quaternary trifluoromethylated amino esters were obtained with excellent enantioselectivities.

**Scheme 32:**
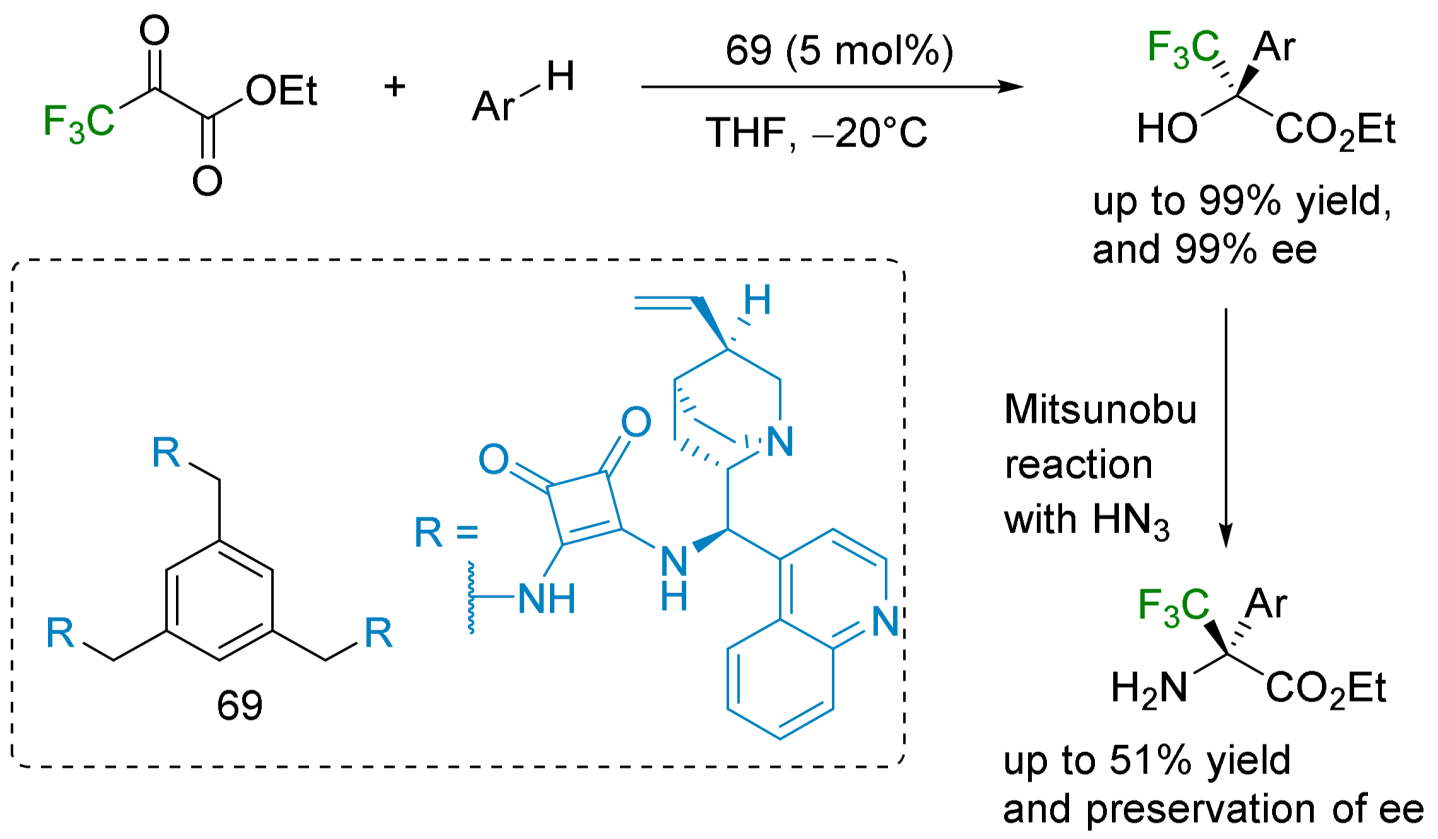
C_3_-symmetric chiral squaramide as an organocatalyst for Friedel–Crafts alkylation [66,67].

Zhou, Dong, and colleagues proposed an alternative strategy towards α-aryl-α-CF_3_-α-amino acids. They developed the direct enantioselective Friedel–Crafts alkylation of trifluoropyruvate, leading to the α-hydroxy ester in excellent yields and enantiomeric excesses [66]. The original C_3_-symmetric chiral squaramide **69** was found to be highly efficient, providing over 90% ee for a large range of substituted indoles (Scheme 32). In a wish to access to the amino ester derivatives, a Mitsunobu reaction was later developed by the same group, preserving the enantiomeric excesses for every example [67].

- Cycloadditions

The opening of 2-trifluoromethyl-2-alkoxycarbonyl aziridine with diverse nucleophiles has proved to be an efficient route towards the synthesis of quaternary α-CF_3_-α-amino acids. The achievements of Katagiri and colleagues on this topic demonstrated that straightforward opening can be achieved with full preservation of stereoselectivity [68,69].

**Scheme 33:**
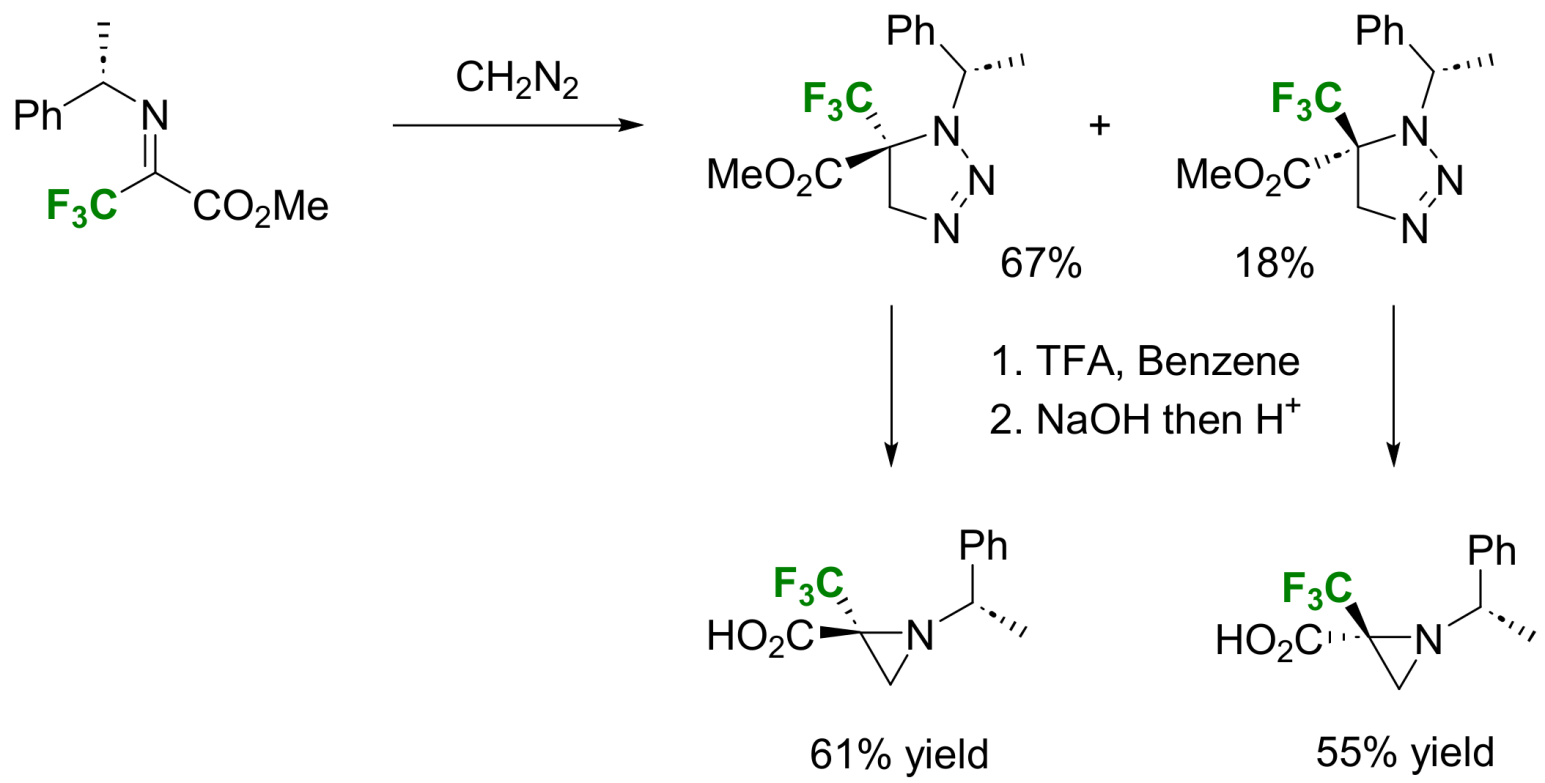
[3 + 2] Cycloaddition towards α-trifluoromethyl aziridine [70].

Consequently, the development of efficient stereoselective synthesis of these trifluoromethylated aziridine is of very high interest and cycloaddition appeared to be a rather straightforward strategy to this end. In 2017, Onys’ko and coworkers developed the diastereoselective [3 + 2] cycloaddition of chiral imino trifluoropyruvate with diazomethane, affording a separable diastereomeric mixture of triazoline (Scheme 33). These cycloadducts can be smoothly converted to the corresponding aziridines upon acidic treatment. Saponification of the methyl ester was also achieved in high yield for both diastereomers without observing any decarboxylation [70].

**Scheme 34:**
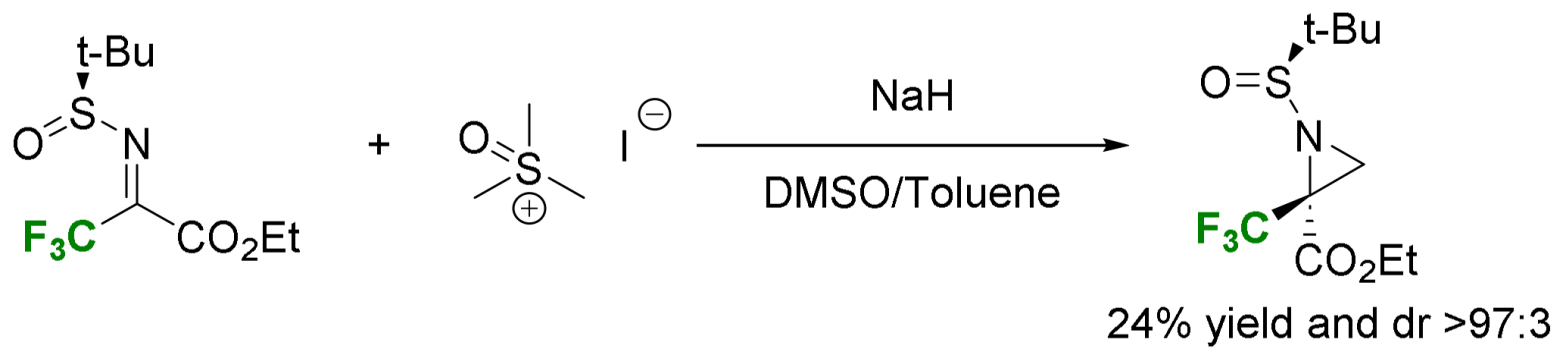
Diastereoslective Aza-Corey–Chaykovsky aziridination [71].

Marsini and coworkers also achieved stereoselective aziridine-2-carboxylate synthesis via the direct aza-Corey–Chaykovsky aziridination of chiral ketimino esters [71]. The use of a chiral sulfinyl auxiliary allowed very high diastereomeric ratios to be obtained for a large diversity of substrates. The member of scope bearing a trifluoromethyl group in the α-position was obtained with a rather low yield but a diastereomeric ratio above the NMR sensitivity threshold (Scheme 34).

**Scheme 35:**
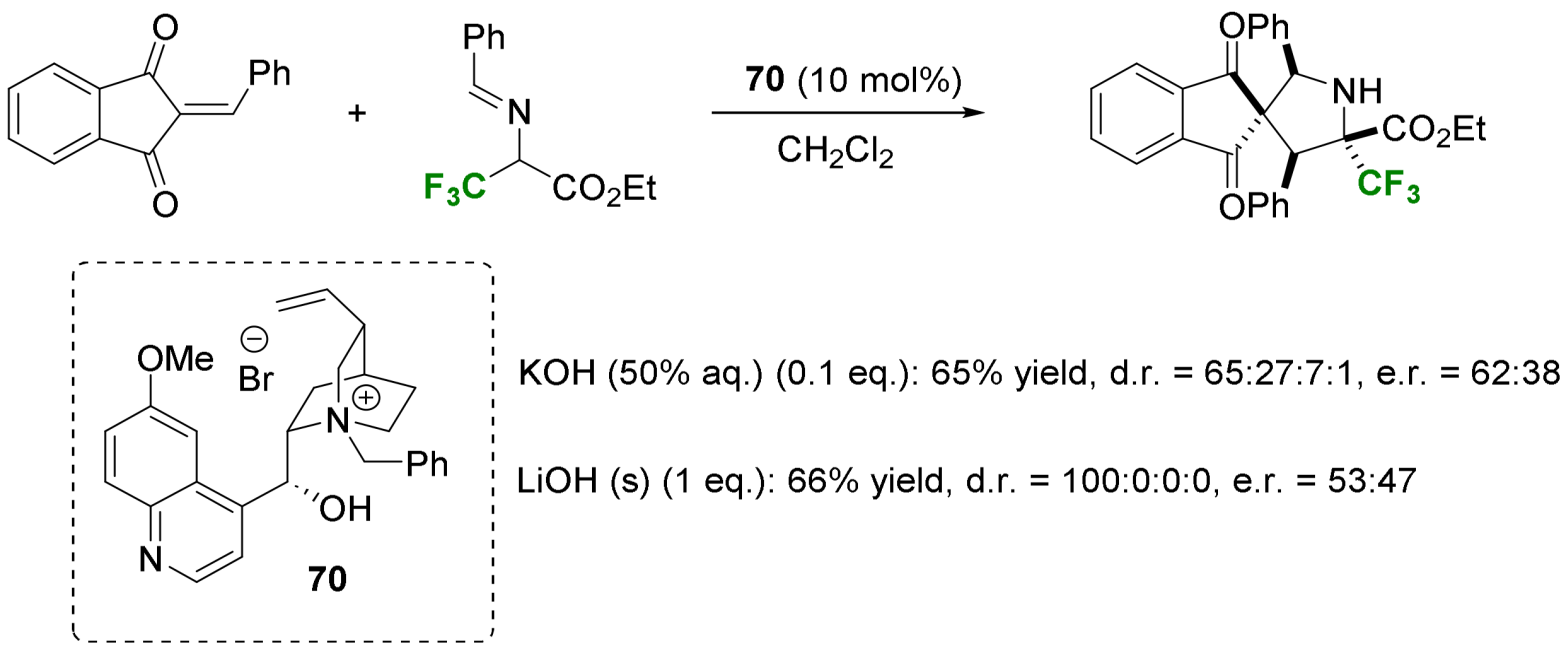
Formal [3 + 2] cyclization between trifluoropyruvate imines and the Michael acceptor [72].

In 2019, a formal [3 + 2] cycloaddition allowed Waser and coworkers to access an original spirocyclic α-CF_3_-α-amino acid [72]. Optimization of the reaction conditions under phase transfer catalysis allowed full diastereoselectivity to be achieved out of four possible isomers. Unfortunately, when they attempted to use the chiral phase transfer catalyst **70** in the reaction, they could not achieve higher than 24% ee (Scheme 35).

- Others

**Scheme 36:**
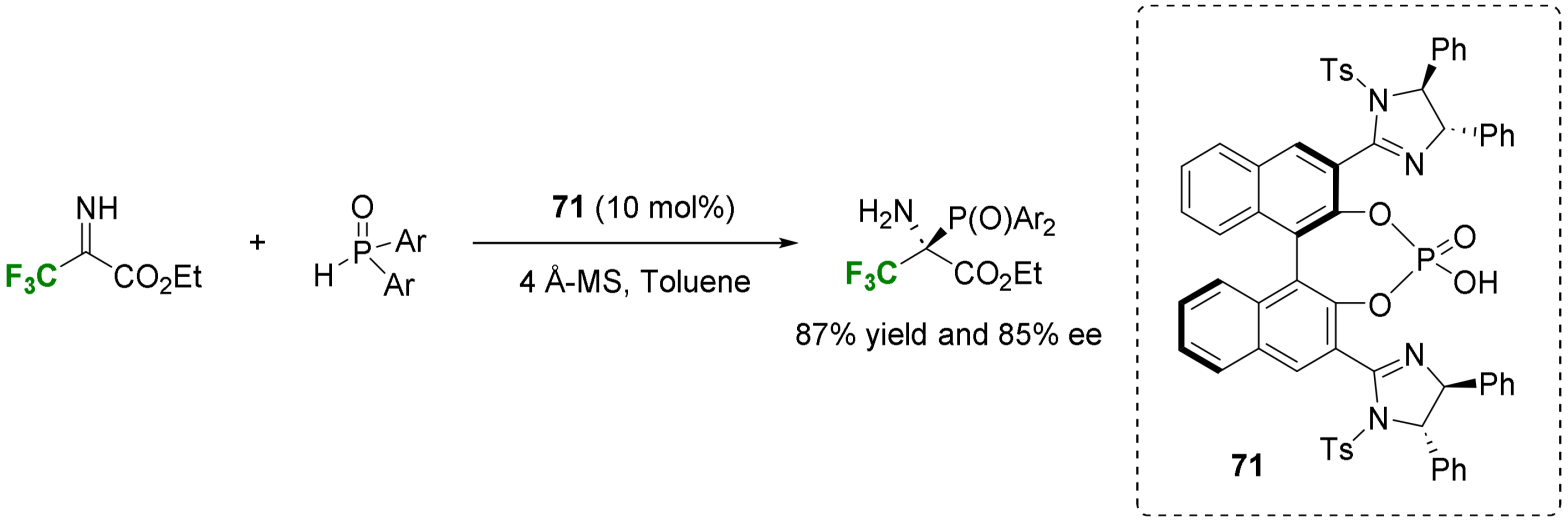
Enantioselective hydrophosphonylation of imino pyruvates [73].

*Hydrophosphonylation*:

Very recently, the Nakamura group expanded the scope of α-CF_3_-α-amino acids by developing the first enantioselective hydrophosphonylation of ketimines with phosphine oxides [73]. Diverse fluorinated unprotected ketimines were tolerated in this new synthetic methodology employing the chiral phosphoric acid **71** as an organocatalyst to provide excellent stereoselectivity. The use of imino trifluoropyruvate as the electrophile for this transformation confirmed that α-phosphonyl-α-trifluoromethyl amino acids can be produced with high yield and stereoselectivity using this strategy (Scheme 36).

**Scheme 37:**
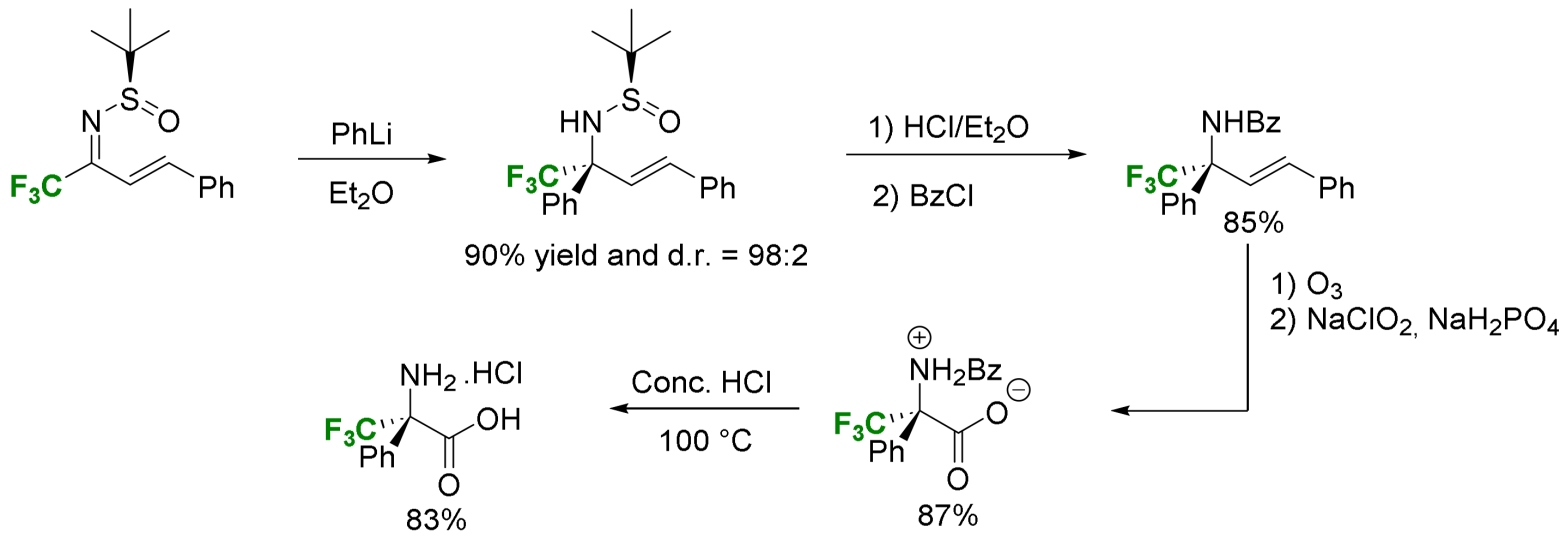
Diastereoselective organolithium addition to trifluoromethyl imines [74].

*Organometallic nucleophiles*:

The use of carbon nucleophiles via organometallic species such as Grignard, organolithium, or organozinc reagents to access α-CF_3_-α-amino acids has been extensively studied and reviewed in the past [4,45]. If it has been established as a very general approach to this end, recent developments in asymmetric examples have been very scarce, as it was found rather challenging to obtain excellent stereoselectivities using organometallic-based strategies. The recent work of Liu and Wu is noteworthy, however, as they managed to obtain very high diastereomeric ratios via the addition of organolithium to α,β-unsaturated trifluoromethyl imines bearing a chiral auxiliary [74]. Subsequent ozonolysis of the protected amine resulted in the corresponding unprotected amino acid in a very high yield (Scheme 37).

**Scheme 38:**
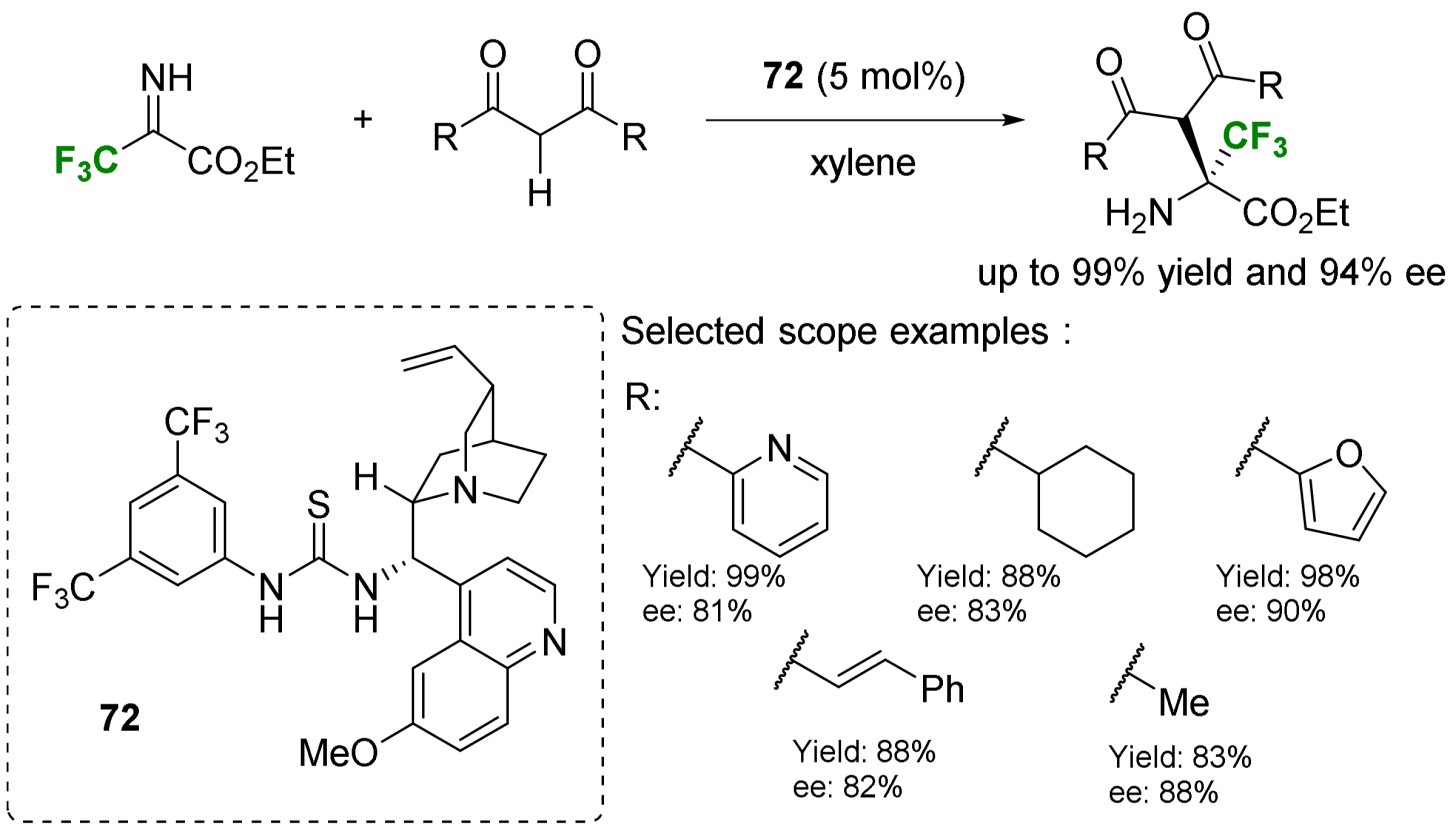
Organocatalyzed asymmetric Mannich-type addition [75].

*Enolizable Nucleophiles*:

Another ubiquitous source of carbon nucleophile is the deprotonation of enolizable compounds and analogs. The Morimoto and Ohshima group developed a methodology allowing access to unprotected α-CF_3_-amino esters via a Mannich-type reaction of diketones to imino esters [75]. The chiral thiourea organocatalyst **72** provided good enantioselectivity for a large range of nucleophiles, providing a robust asymmetric synthetic strategy towards novel trifluoromethylated amino acids (Scheme 38).

**Scheme 39:**
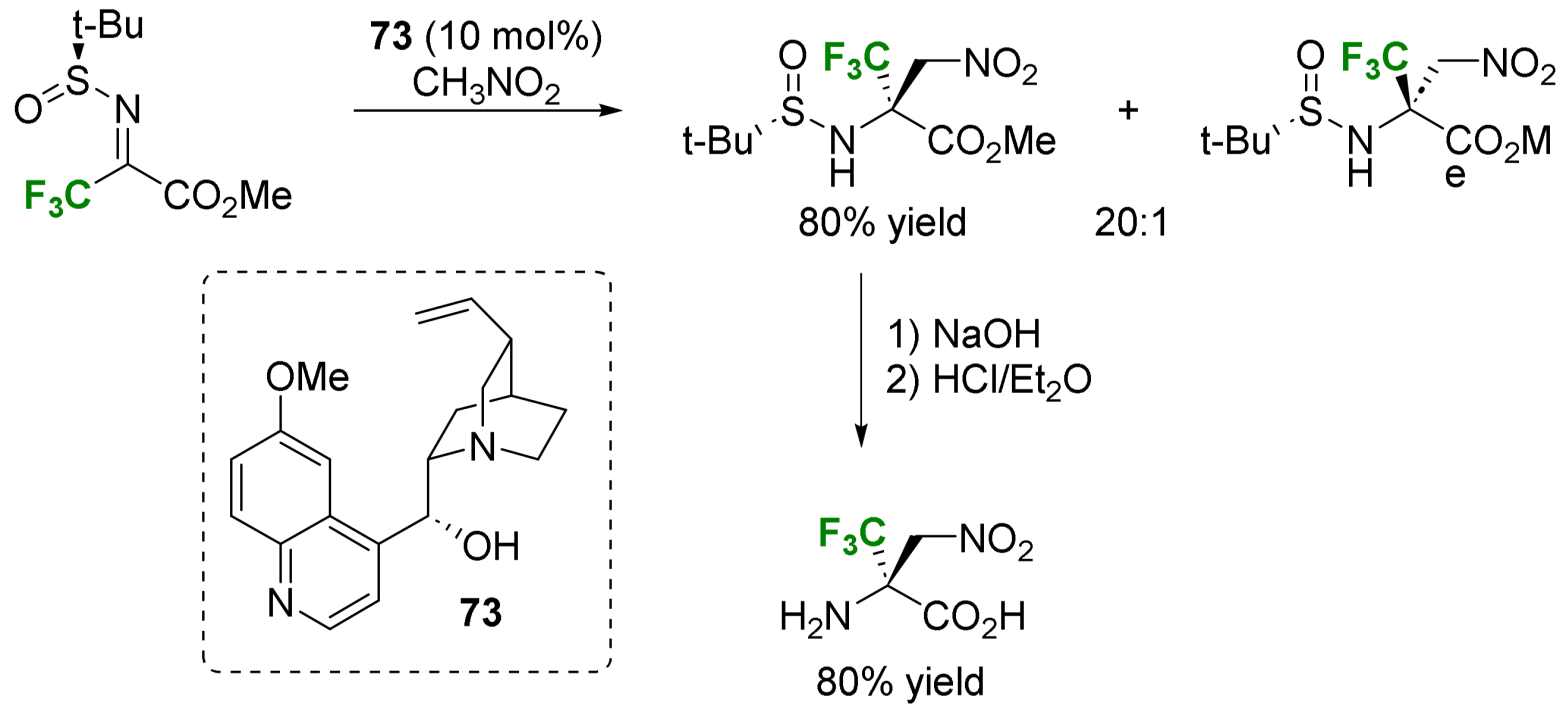
Stereoselective Henry reaction to β-nitro-amino acids [76].

More recently, the Raasukana and Onys’ko group combined the use of a trifluoropyruvate bearing a chiral auxiliary and the chiral organocatalyst **73** to achieve the diastereoselective Henry reaction [76]. α-Trifluoromethyl-β-nitro-amino acids were made accessible via this synthetic route after full deprotection of the major diastereomer in high yield (Scheme 39).

#### 2.3.2. Via Introduction of the Carboxyl Group

One of the major synthetic strategies to produce chiral non-racemic amino acids is the asymmetric Strecker reaction followed by hydrolysis of the so-formed nitrile group. This straightforward way to introduce a carboxyl group was extensively applied to the synthesis of α-CF_3_-α-amino acids using trifluoro ketimines as initial substrates. Most of these contributions have been reviewed in the past [4,45] and are out of the scope of this review considering the date of publication. Recent work on the Strecker reaction focused on access to cyclized α-CF_3_-α-amino acids.

**Scheme 40:**
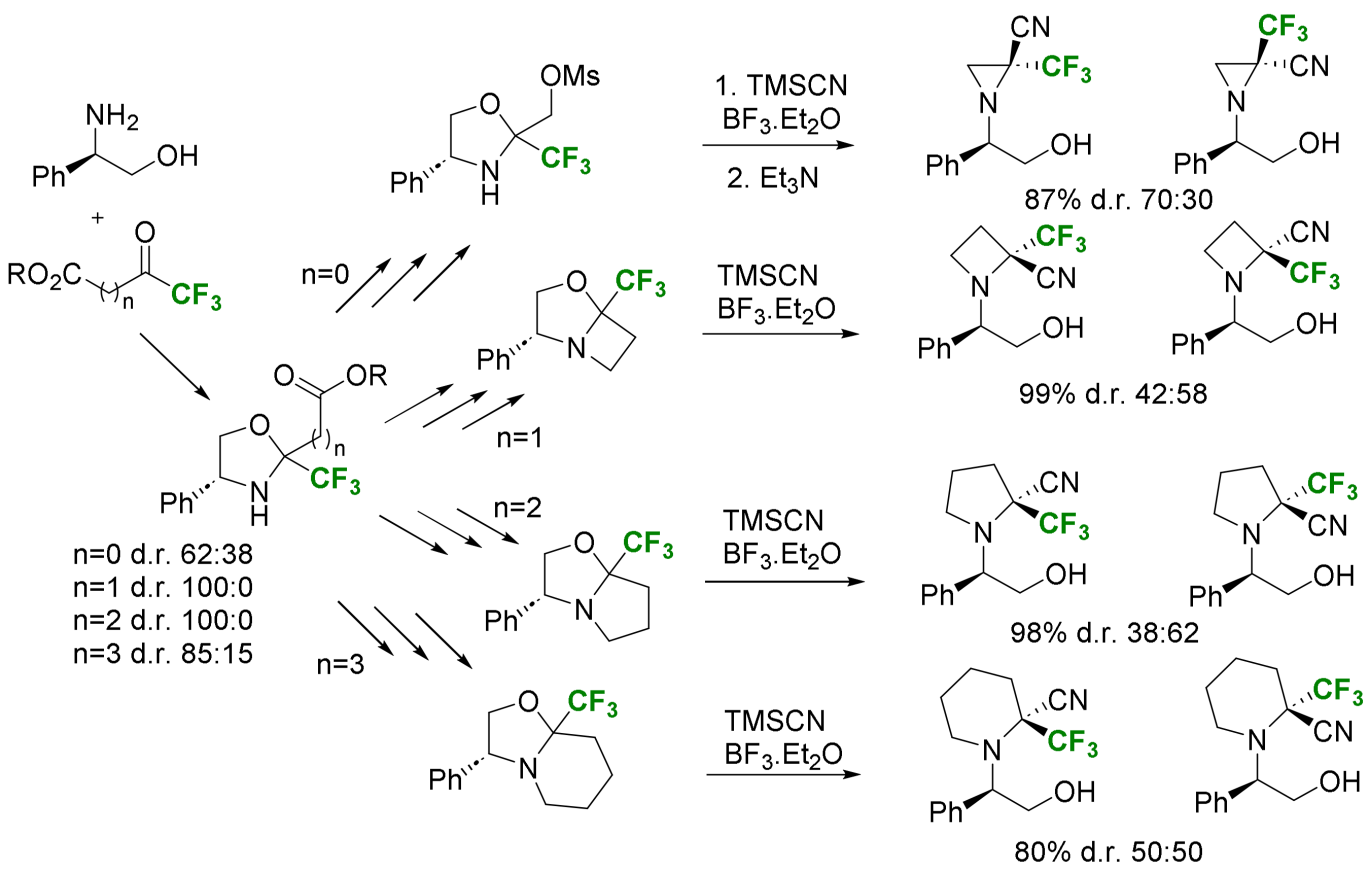
Strecker strategies towards cyclic trifluoromethylated amino acids [77,78,79].

Our group has dedicated much effort to the development of a general methodology to access cyclic residues of various ring sizes. The strategy relied on the synthesis of chiral oxazolidines by condensation of enantiopure phenylglycinol with diverse trifluoromethylated ketoesters. If the Strecker reactions on these chiral cyclic intermediates assisted by boron trifluoride Lewis acid proceeded with very high yields, only modest diastereoselectivities were obtained. Ultimately, diastereomeric mixture have been separated in order to obtain enantiomerically pure batches of every amino acid (Scheme 40).

**Scheme 41:**
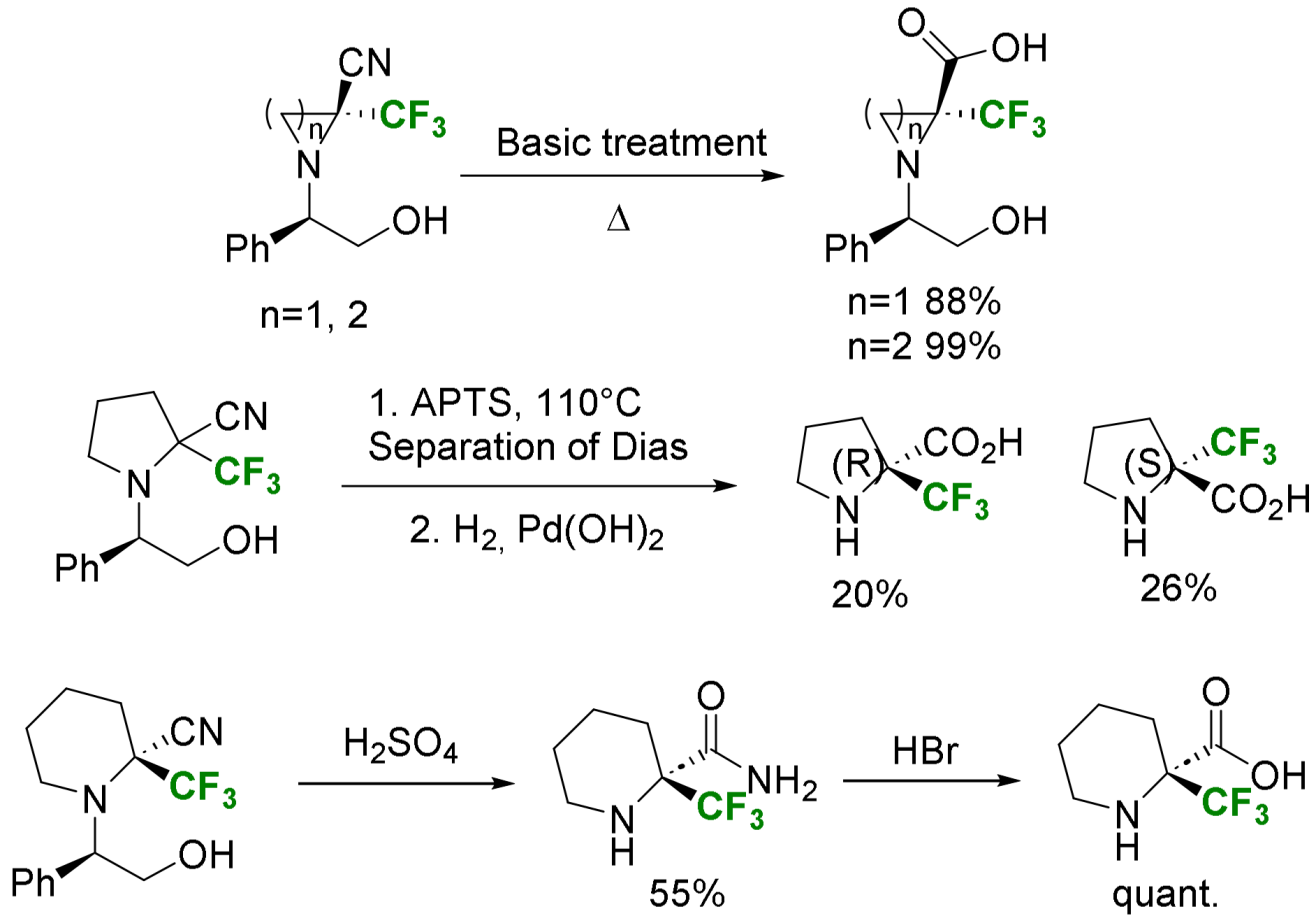
Towards three-, four-, five- or six-membered ring cyclic α-trifluoromethyl amino acids [77,78,79].

The so-formed amino nitriles were successfully hydrolyzed to the corresponding amino acids (Scheme 41). Aziridine [77] and azetidine [78] derivatives were hydrolyzed in very high yields using basic conditions. The fully deprotected enantiopure α-CF_3_-α-carboxyazetidine was successfully obtained after cleavage of the chiral auxiliary, while the aziridine was efficiently incorporated into a dipeptide prior to *N*-deprotection. It is noteworthy that azetidine amino nitrile could also be hydrolyzed under acidic conditions, revealing a novel route towards α-CF_3_-homoserine via the opening of the 4-membered ring. The pyrrolidine- and piperidine-derived amino nitriles needed an acidic treatment to produce the carboxyl group [79]. Ultimately, fully deprotected α-CF_3_-proline and α-CF_3_-pipecolic acid were obtained as enantiomerically pure samples.

**Scheme 42:**
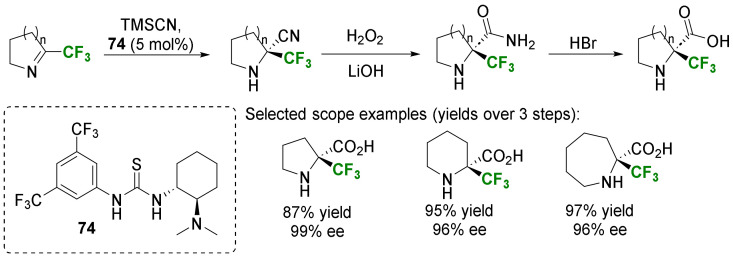
Organocatalyzed enantioselective Strecker reaction [38].

Concomitantly, the Nenajdenko group reported a catalytic route towards the synthesis of cyclic α-CF_3_-α-amino acids with five-, six- and seven-membered rings [38]. The synthetic strategy relied on the direct enantioselective Strecker reaction on cyclic trifluoromethyl ketimines using the Takemoto thiourea **74**. The stepwise hydrolysis of amino nitriles to amino acids via amino amide intermediates was necessary to avoid a retro-Strecker reaction due to the elimination of HCN. Ultimately, excellent overall yields and enantioselectivities were obtained for diverse cyclic α-CF_3_-α-amino acids (Scheme 42).

#### 2.3.3. Via Introduction of the Amino Group

**Scheme 43:**
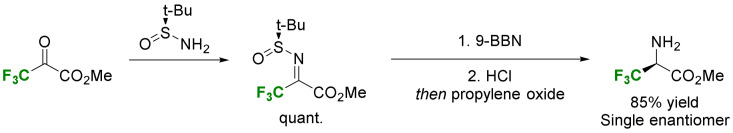
Reductive amination to trifluoroalanine [76].

The construction of the carbon–nitrogen bond via reductive amination of trifluoropyruvate has been one of the most straightforward strategies to access enantioenriched 3,3,3-trifluoroalanine [4,45]. Recently, Rassukana and Onys’ko reported an improvement of this method via the use of chiral sulfinylimine as an auxiliary to provide total enantioselectivity to the reduction by 9-BBN (Scheme 43). The subsequent cleavage of the chiral auxiliary under acidic conditions provided the methyl ester of 3,3,3-trifluoroalanine as a single enantiomer in very good yield over three steps [76].

**Scheme 44:**
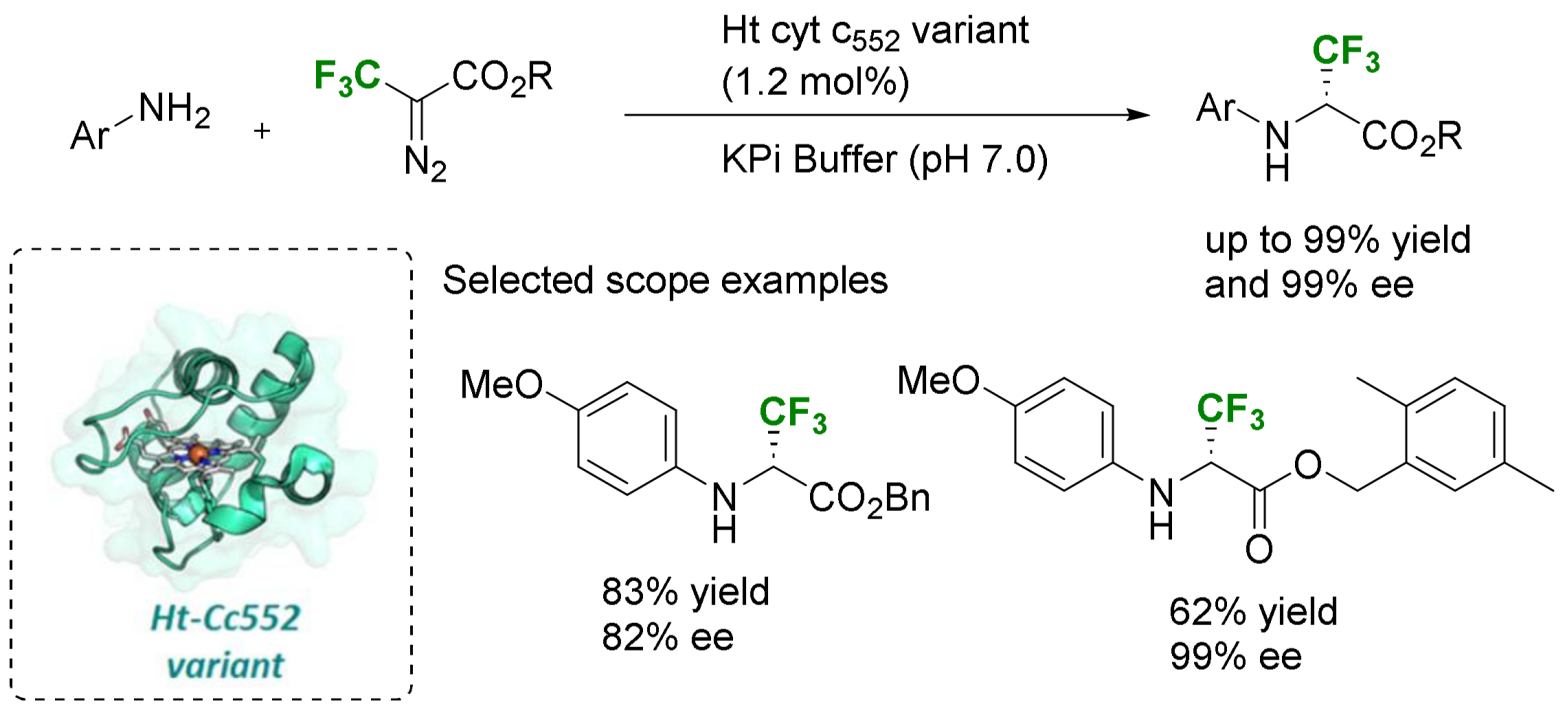
Biocatalyzed asymmetric carbene insertion [80].

An alternative approach to the formation of carbon–nitrogen bonds for the formation of α-CF_3_-α-amino acids is the asymmetric insertion of carbenoids into N-H bonds. A recent report from Fasan, Khare, and colleagues described the development of a biocatalytic strategy towards the synthesis of 3,3,3-trifluoroalanine via N-H carbene insertion [80]. The directed evolution of the metalloenzyme Ht-Cc552 via selective mutation of the active site allowed the identification of a variant of the biocatalyst, providing improved yields and enantioselectivity (Scheme 44). The authors offered an in-depth study providing mechanistic insights to rationalize the stereoselectivity observed.

## 3. Applications

### 3.1. Biological Applications of α-Fluoroalkyl-α-Amino Acids and Analogues

#### 3.1.1. Enzyme Inhibitors and Bioactive Small Molecules

**Scheme 45:**
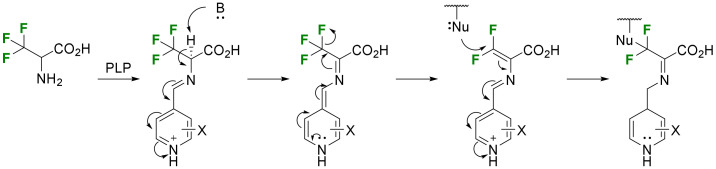
Trifluoroalanine as suicide substrate of PLP-dependent enzymes [87,88].

Biological applications for α-fluoro-containing molecules are rather large. α-Fluoroalkyl α-amino acids are, on the other hand, less represented apart from α-difluoromethylornithine. This largely studied case will not be reviewed here as it has been performed recently [81,82]. These α-fluoroalkyl α-amino acids motifs can be found in various domains, such as enzyme inhibitors or bioactive small molecules. The most known example is the use of fluorinated amino acids as potential irreversible inhibitors of pyridoxal phosphate (PLP)-dependent enzymes such as decarboxylases, transaminases, and racemases [83,84,85]. For instance, β,β,β-trifluoroalanine is a well-known suicide substrate of PLP-dependent enzymes [83,86,87,88] (Scheme 45). Inactivation of the enzyme is provided by the leaving group character of the fluorine atom, placed in the β-position of a natural mimic.

**Figure 3:**
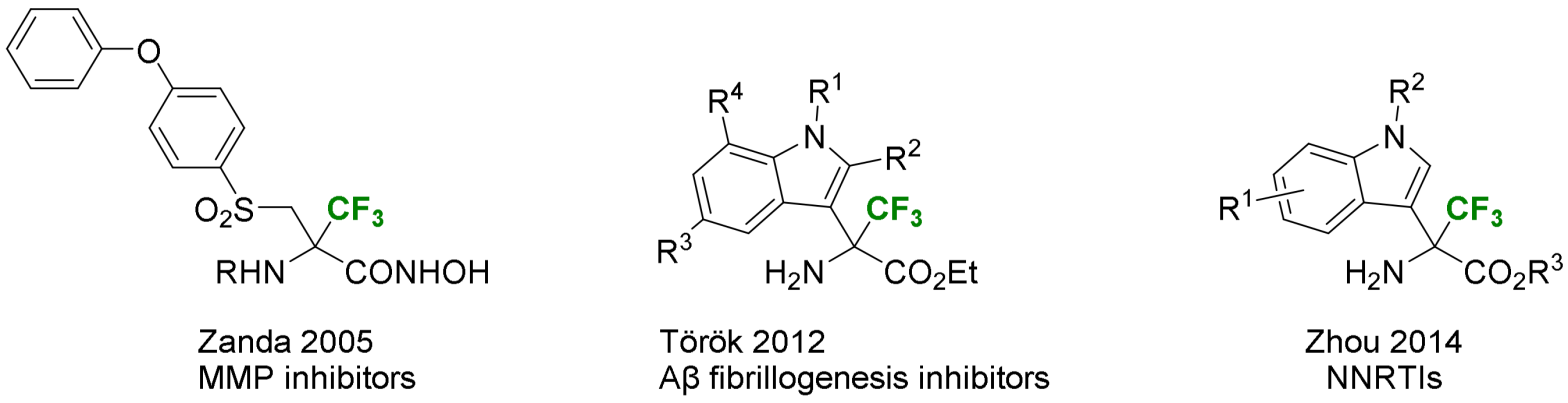
Some biologically active α-fluoroalkyl α-amino acid derivatives [89,90].

Some of these enzymes are of medicinal importance as targets for the treatment of various diseases [81]. α-Trifluoromethyl-α-amino acids have also been studied as protease inhibitors by Zanda et al. in 2005 [89]. The group investigated the effects of the trifluoromethyl group on the inhibition of matrix metalloproteinases (MMPs) by the synthesis of α-trifluoromethyl-α-amino-β-sulfone hydroxamates. Small organofluorine compounds were also evaluated as inhibitors of β-amyloid (Aβ) self-assembly in 2012 by Török et al. [90]. Although the α-trifluoromethyl-α-amino acid did not show the best results of all the 106 compounds tested, two CF_3_-containing compounds showed strong inhibition of Aβ self-assembly by thioflavin-T fluorescence spectroscopy assays and atomic force microscopy (AFM) techniques, with fibril inhibition ranging from 32 to 44% and oligomerization inhibition ranging from 40 to 20% for these promising fluorinated compounds (Figure 3).

Zhou’s team, on the other hand, investigated a series of non-nucleoside reverse transcriptase inhibitors (NNRTIs) derived from indole-based *α*-amino acids [67]. They evaluated their inhibitory activities by performing a TZM-bl cell assay on HIV virus type HIV-1_IIIB_. SAR studies then showed EC_50_ values ranging from 46.473–0.045 μM and highlighted the importance of chirality towards inhibition. This importance of chirality was also observed by Natile et al. in 2005 [91] with platinum-based antitumor drugs containing pure α-trifluoromethylalanine (α-TfmAla) as the ligand. The overall in vitro studies did not reveal impressive results, although the activity of the complexes depended rather strongly upon the configuration of the *α*-TfmAla ligand.

#### 3.1.2. Building Blocks for Heterocyclic Compounds

**Figure 4:**
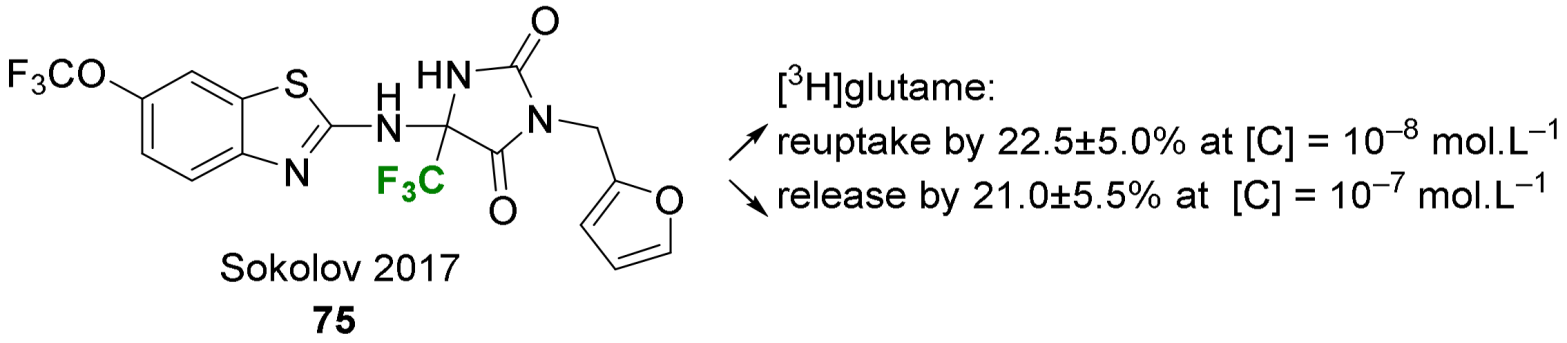
Trifluoromethyl-containing heterocycle derivative of the drug riluzole [92].

α-Trifluoromethyl motif is also to be found in others diverse biological applications, such as potential neurotransmitters like α−CF_3_-containing triazolyl amino acids. Such an example has been published in 2015 by Osipov’s team [51]. A more important example is presented by Sokolov, who developed new trifluoromethyl-containing heterocycle derivatives of the drug riluzole in 2017 [92]. This drug has been now used for some time to reliably slow the progression of amyotrophic lateral sclerosis. The group investigated the compounds’ influence on neuronal NMDA receptors, as well as the release and reuptake of the neurotransmitter glutamate. The study demonstrated an interesting result when compound **75** (Figure 4) was found to have comparable glutamate transport systems as riluzole, with an augmented reuptake of 22.5 ± 5.0% at a 10^−8^ M concentration and a diminished release by 21.0 ± 5.5% at 10^−7^ M.

**Figure 5:**
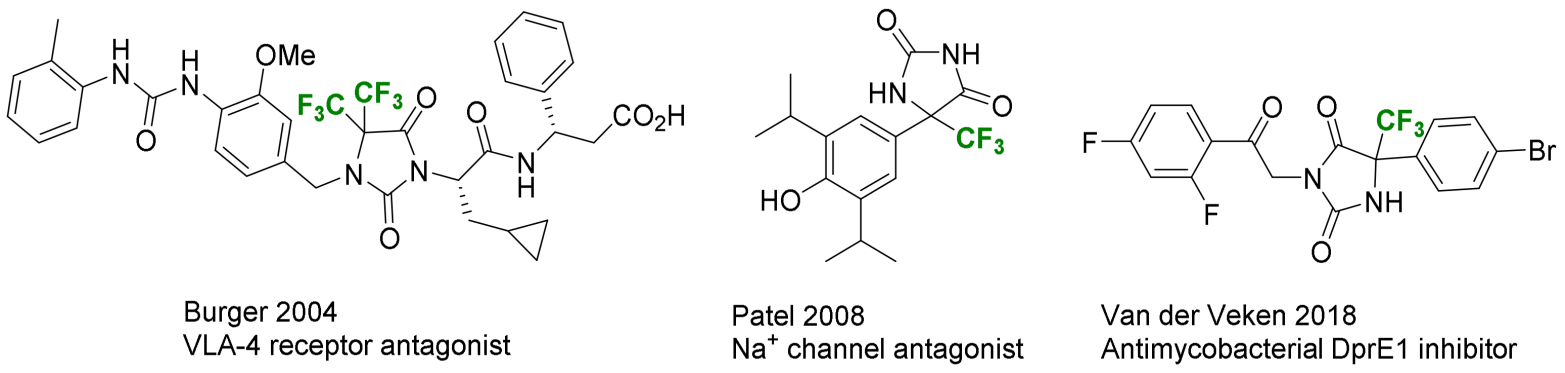
Biologically active heterocyclic analogues [93,94,95].

Other examples of α-fluoro α-amino acid motifs are also reported in heterocycle-containing pseudopeptides. Some methodological developments for the incorporation of fluoro-modified amino acids via heterocyclic building blocks such as hydantoins to improve their therapeutic profile can be found in the literature (Figure 5).

Burger et al., for example, presented in 2004 [93] the synthesis of a promising VLA-4 receptor antagonist incorporating a trifluoromethyl motif to enhance its therapeutic profile. The IC_50_ in a cell attachment assay using VLA-4-expressing U937 cells and its natural ligand VCAM-1 was determined to 5.55 nM. This trifluoromethyl hydantoin motif was also used in 2008 by Patel’s team as potential sodium channel antagonist [94]. More recently, Van der Veken in 2018 [95] integrated this motif in one potential antimycobacterial DprE1 inhibitor without improving the potency of the scaffold by incorporating the α-fluoro α-amino acid.

### 3.2. Incorporation in Peptides and Modulation of the Biophysical and Biological Properties of Peptides

#### 3.2.1. Incorporation in Peptides

- Solution Phase

**Scheme 46:**
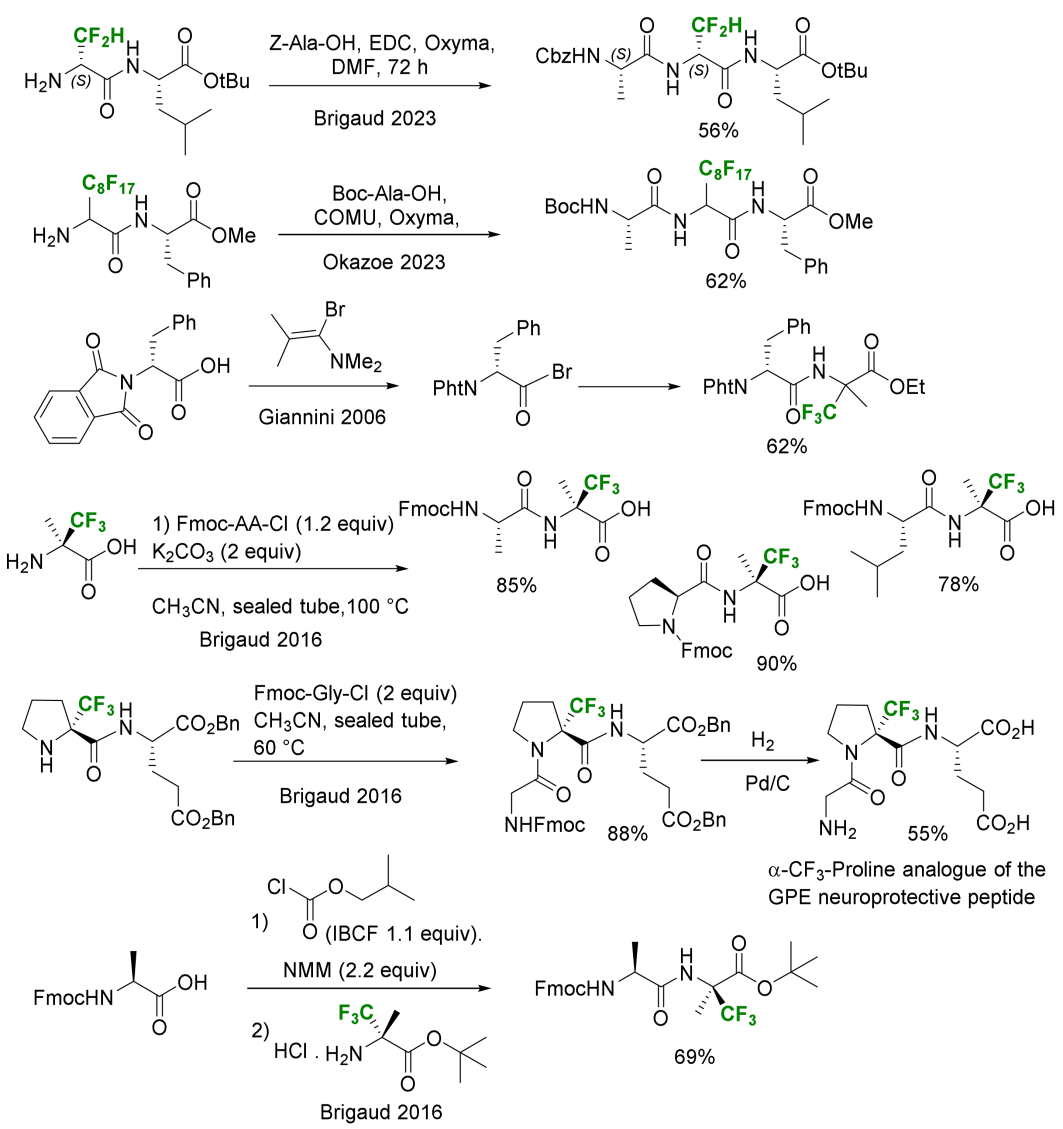
Selected examples of *N*-terminal position peptide coupling reactions of α-Tfm-amino acids [44,98,99,100,101].

The incorporation of α-amino acids with fluorinated side chains in peptides does not present any particular difficulties when the fluorine atoms are located at the γ- or ω-positions and standard peptide coupling reaction conditions can be used for solution phase or SPPS (for recent examples see [96] and references cited). The peptide coupling reactions at the *C*-terminal position of α-fluoroalkyl-α-amino acids can also be achieved in good yields by classical procedures [77,97,98]. However, when fluorine atoms are present at the β-position of the α-amino acids, their strong electron-withdrawing effect dramatically decreases the nucleophilicity of the nitrogen atom of the α-amino acids, and specific activation methods are generally required for their *N*-coupling reactions. The coupling of β,β-difluoroalanine can be achieved at its *N*-terminal position using the EDC/oxyma activation [44]. Glycine with a perfluoroalkylated side chain was recently incorporated in a peptide chain using the COMU/Oxyma system [99]. However, the peptide coupling reactions at the *N*-terminal position of α-Tfm-amino acids, especially the hindered quaternarized ones, such as α-Tfm-alanine, required the activation of the amino acid as an acid halide or a mixed anhydride. The amino acid bromides are prepared by a reaction of the amino acid with Ghosez’s reagent [100]. The amino acid chlorides were conveniently prepared by treatment of the amino acid with an excess of thionyl chloride and evaporation [97,98]. The coupling reaction of Fmoc-amino acid chlorides were conveniently achieved at the *N*-terminal position of α-Tfm-alanine and also α-Tfm-proline [101]. The mixed anhydride was prepared by a reaction of the amino acid with isobutyl chloroformate (IBCF) in the presence of *N*-methylmorpholine (NMM) [98]. Several examples of *N*-terminal position peptide coupling conditions are presented in Scheme 46.

- Solid Phase Peptide Synthesis (SPPS)

**Scheme 47:**
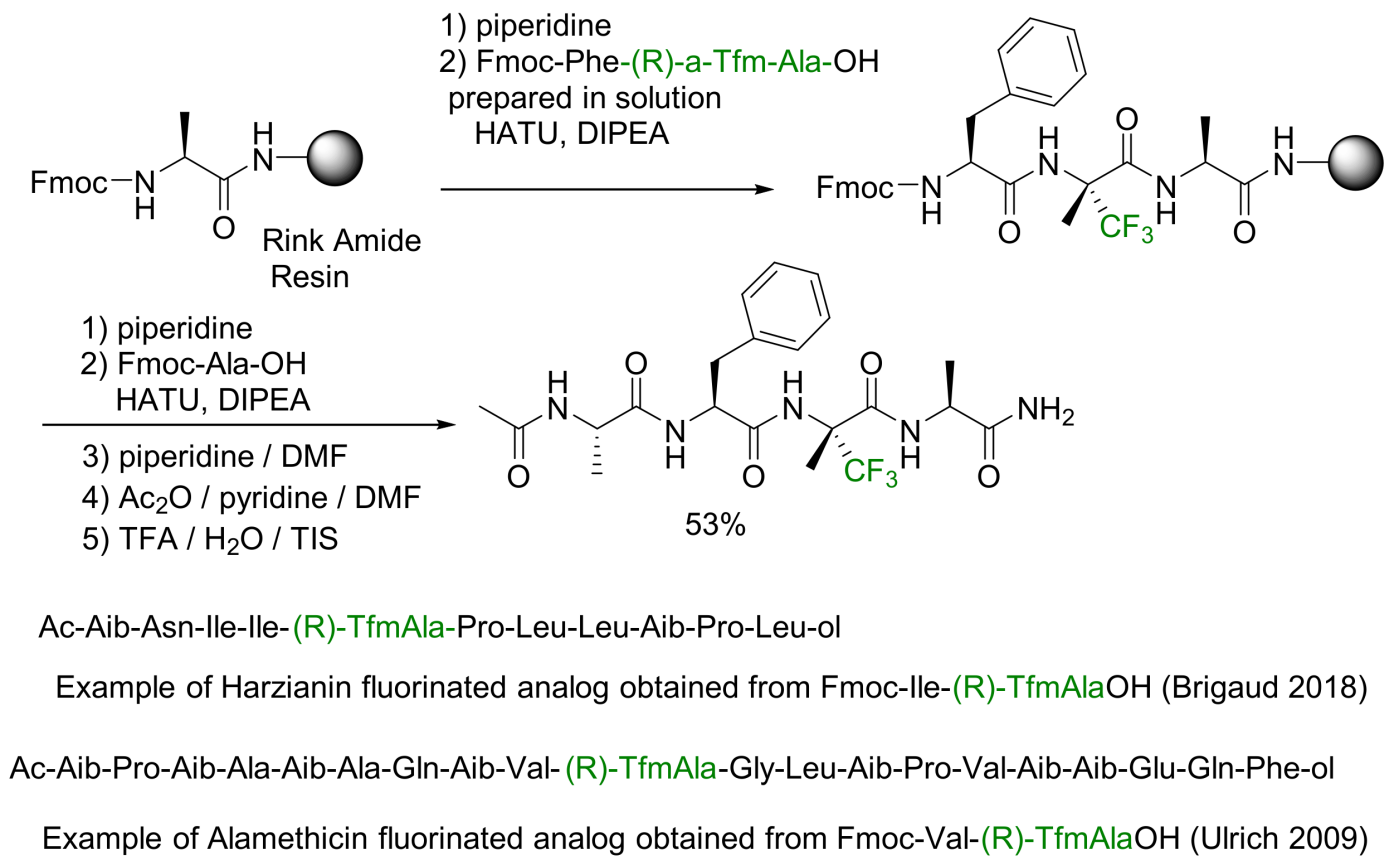
Selected examples of peptides incorporating α-Tfm-alanine synthesized by SPPS [103,104].

The direct incorporation of α-Tfm-AAs in the middle of a peptide chain is still a challenge, and all attempts to perform on resin the coupling of an amino acid at the *N*-terminal position of the deactivated fluorinated amino acid have failed so far. The alternative strategy implemented for the incorporation of such amino acids in long peptides by SPPS is the incorporation of Fmoc-protected dipeptide building blocks resulting from the solution phase coupling (Scheme 47) [102]. According to this strategy, numerous long peptides of the peptaibol series, such as harzianin [103] and alamethicin [104], have been prepared notably for ^19^F NMR studies of interactions with membranes.

#### 3.2.2. α-Fluoroalkyl Amino Acid-Containing Peptides for Biological Applications

- Hydrophobicity modulation and biological applications

The modulation of peptide hydrophobicity by the incorporation of fluorinated amino acids is mostly reported and well documented in the γ- or ω-fluoro-substituted series [96,105,106]. Generally, monofluorination lowers the hydrophobicity compared to the non-fluorinated analogues while di- and trifluorination increase it. Because of their difficult synthesis, the hydrophobicity modulation assessment of peptides incorporating α-fluoroalkyl chains has been only recently investigated. Different approaches have been considered for this purpose.

**Figure 6:**
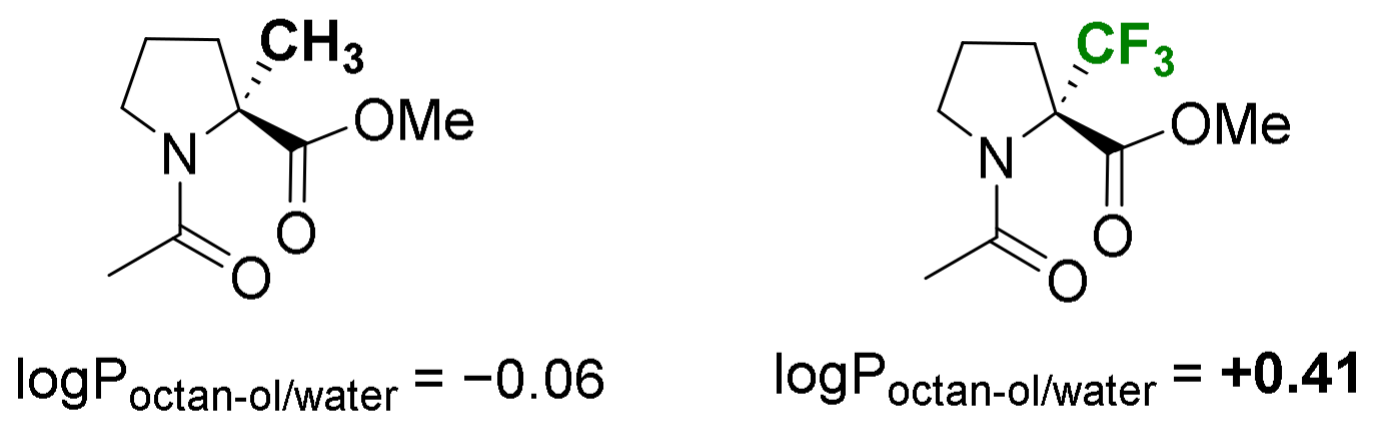
The logP_octan-ol/water_ values for methyl esters of *N*-acetyl prolines [107].

The first method to compare the hydrophobicity of fluorinated amino acids and their hydrogenated analogues is to measure their experimental octan-1-ol/water partitioning (logP) values by NMR. For instance, the logP values of methyl esters of the *N*-acetyl α-trifluoromethylproline and α-methylproline were measured by Kubyshkin and Budisa [107]. The result was a significant hydrophobicity increase for the trifluoromethylated analogue (Figure 6).

**Figure 7:**
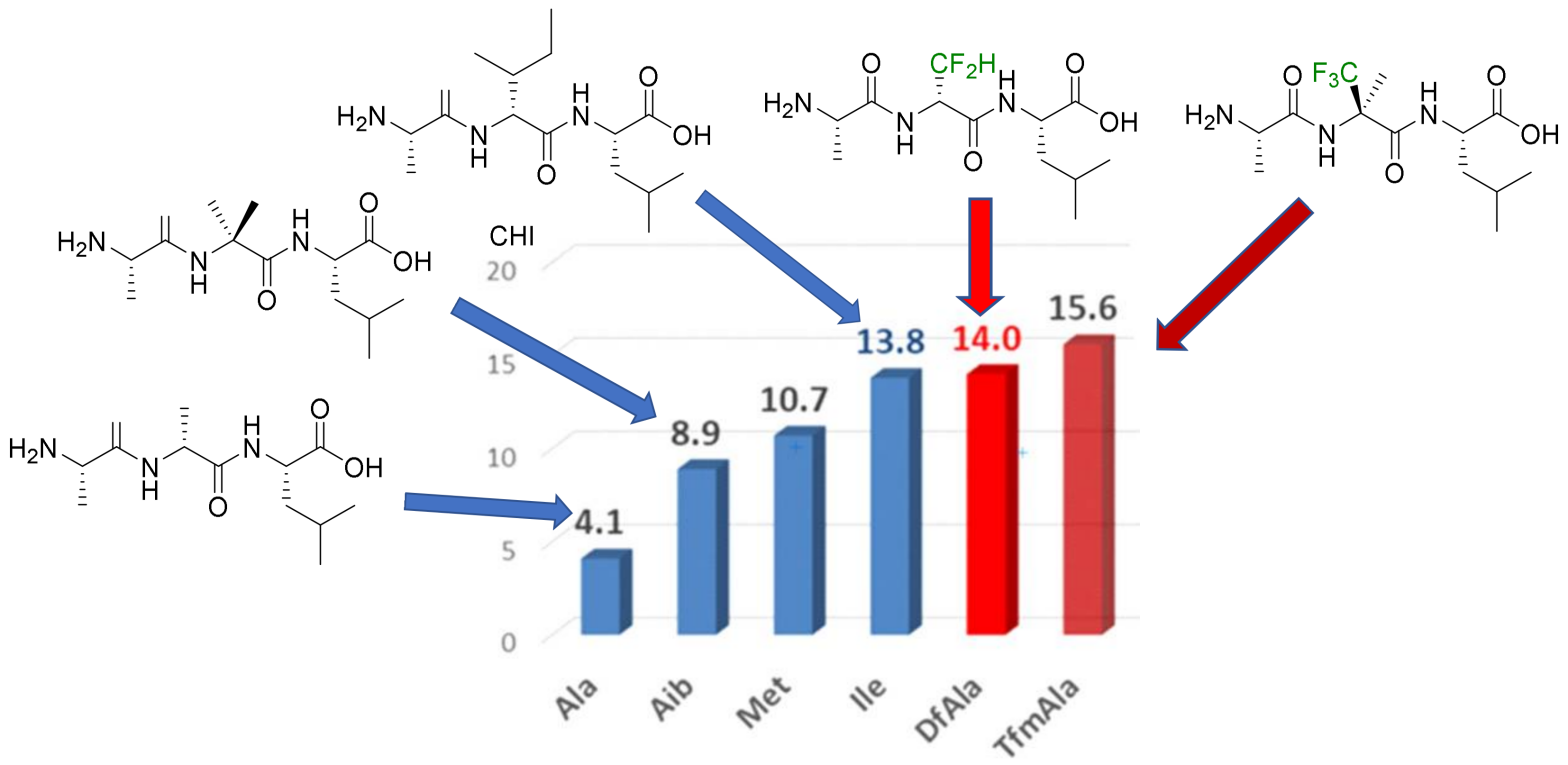
Hydrophobic contributions of α-fluoroalkyl amino acids compared to their non-fluorinated analogues using the chromatographic hydrophobic index (CHI) method [108,109].

A second approach consists of determining the HPLC hydrophobic index (CHI) of peptides [108]. According to this method, we measured the CHI of peptides incorporating a α-fluoroalkyl amino acid by comparison with their non-fluorinated analogues [109]. This method allows an accurate assessment of the local hydrophobic contribution of the fluorinated side chain compared to an aliphatic side chain (Figure 7). Di- and trifluorinated amino acids, such as difluoroalanine (DfAla) and trifluoromethylalanine (TfmAla), provided an increased local hydrophobicity compared to alanine and aminoisobutyric acid (Aib). The hydrophobicity index shift dCHI (CHI_DfAla_ − CHI_Ala_ = 9.9) constitutes a quantitative assessment of the increased hydrophobic contribution of the CF_2_H substituent compared to the methyl group of alanine [44]. It is very interesting to note that despite its smaller van der Waals volume, the CF_2_H group provides nearly the same hydrophobic contribution to the side chain of isoleucine (Ile), which is known to be the most hydrophobic aliphatic amino acid (CHI_DfAla_ − CH_IIle_ = 0.2). Similarly, trifluoromethylalanine provides an increased hydrophobicity compared to Aib (CHI_TfmAla_–CH_Aib_ = 6.7) (Figure 7) [109].

This trend has been applied by us for the design of small hydrophobic peptides for β-amyloid aggregation inhibition [110]. As hydrophobic amino acids and/or α-aminoisobutyric acid (Aib) are known to act as β-sheet breakers, the H-Ala-Aib-Leu-OH and H-Ala-(*R*)-Tfm-Ala-Leu-OH peptides were investigated as inhibitors of Aβ42 aggregation. These peptides were evaluated by the thioflavinT (ThT) binding assay and both showed an inhibition of the kinetics of fibril formation at high concentrations. The Tfm group-containing peptide gave significantly better inhibition results because of its increased hydrophobicity and then showed a good affinity with the hydrophobic region of Aβ.

**Figure 8:**
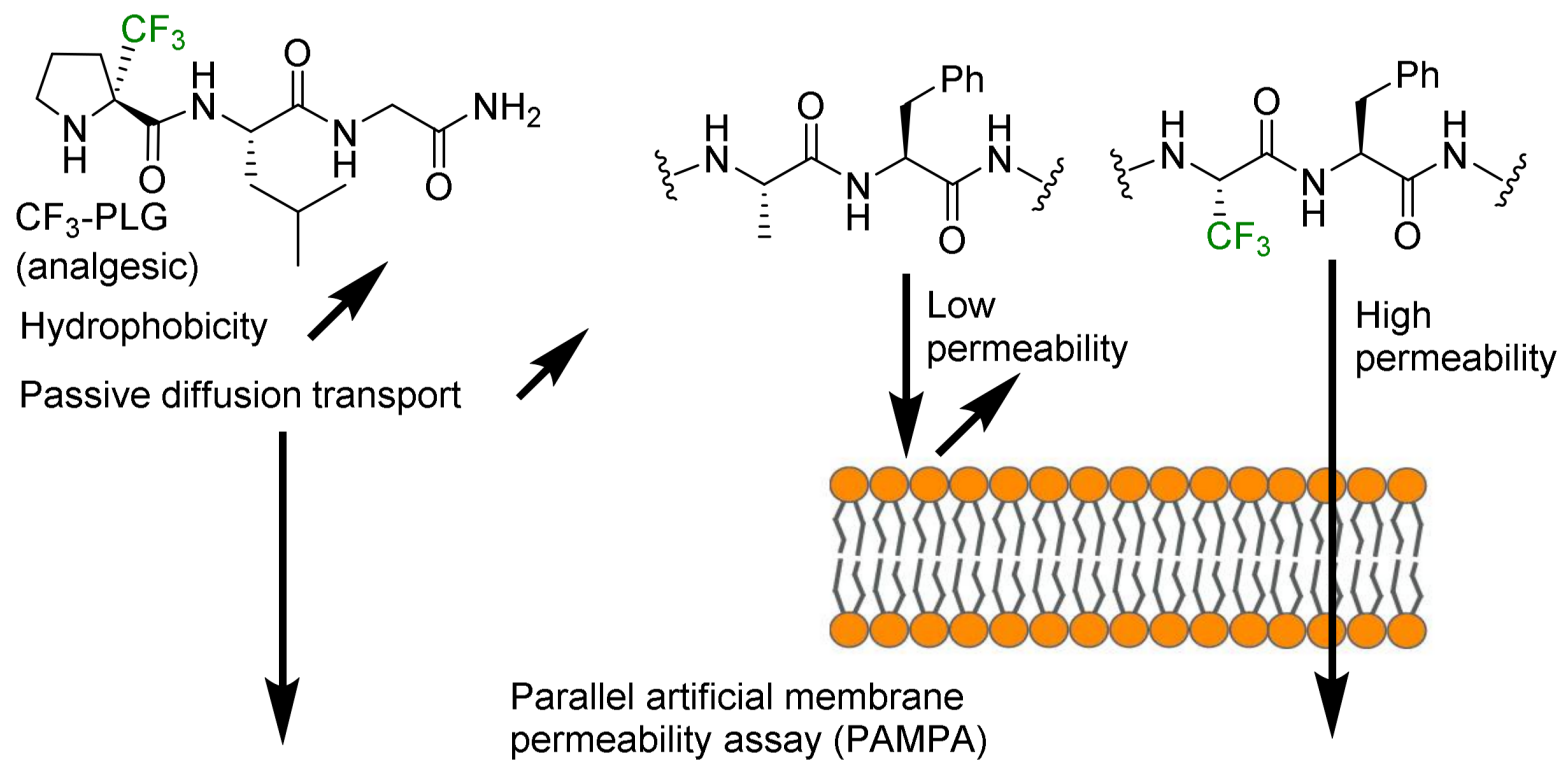
PAMPA method for measuring the increased passive diffusion of α-Tfm-AAs containing short peptides through membranes [114].

Another method reported in the literature for the evaluating the hydrophobicity of fluorinated peptides is the parallel artificial membrane permeability assay (PAMPA) [111]. This method has been implemented to investigate the ability of fluorinated analogues of the PLG peptide (MIF-1) to cross the blood–brain barrier (BBB). The fluorinated analogue of PLG with an α-trifluoromethylproline in place of the proline showed better analgesic activity [112]. This improved biological activity was related to increased hydrophobicity and the ability to cross the BBB through passive diffusion, as showed by PAMPA (Figure 8) [113]. In a similar manner, the PAMPA method has been recently used to demonstrate that a CH_3_ to CF_3_ substitution is a useful strategy for increasing the membrane permeability of di- and tri-peptides (Figure 8) [114].

Very recently, the high ability of perfluoroalkylated chain-containing tripeptides to transport a hydrophilic dye (Alexa Fluor 647) into cells has been reported [99]. This could constitute an interesting strategy for the design of fluorinated drug delivery carriers. Moreover, this study underlined the importance of controlling the absolute configuration of the R_F_-containing amino acid of the cell-penetrating peptide.

- Increased resistance towards proteases and protease inhibitor design

**Figure 9:**
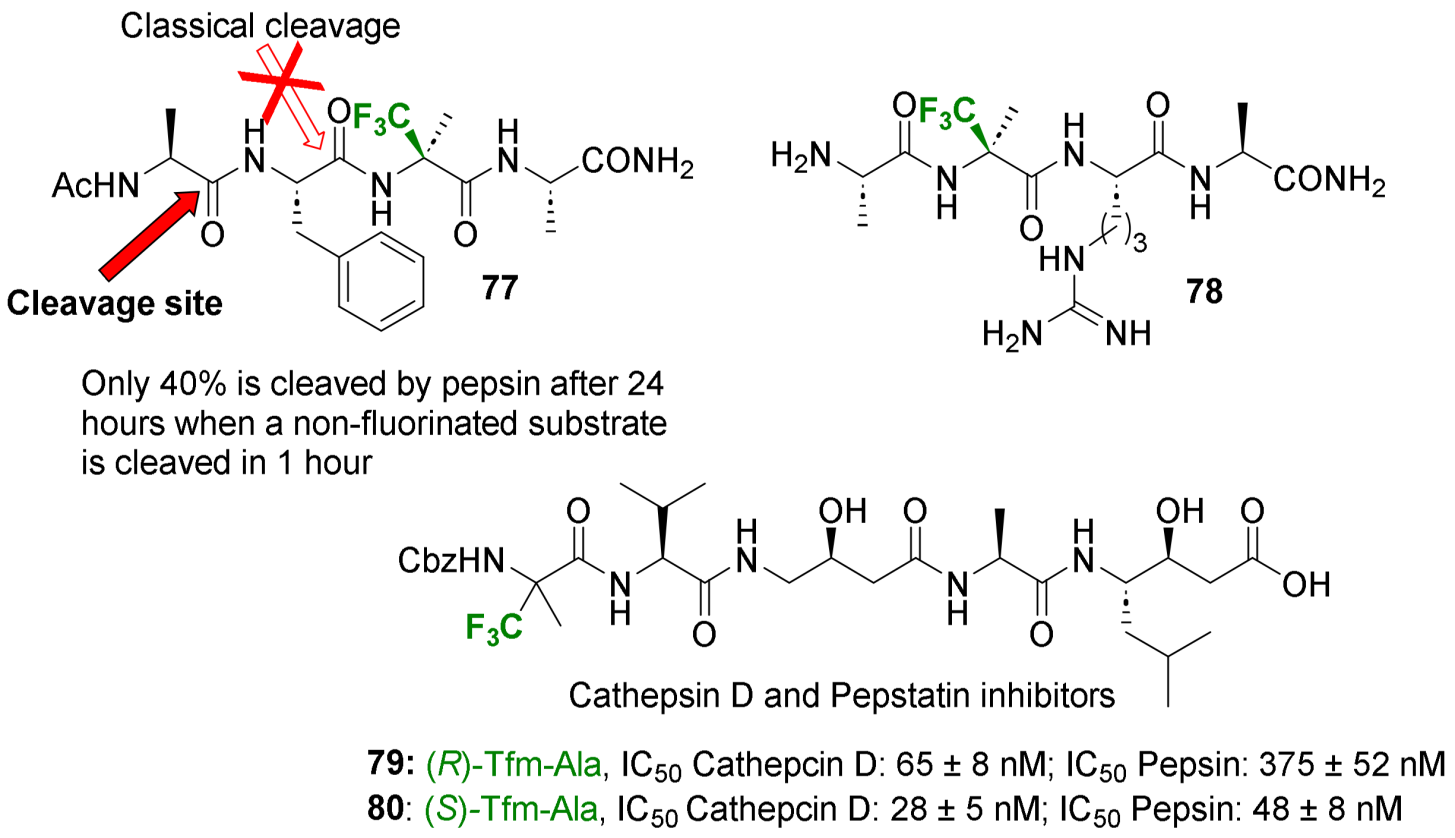
TfmAla-containing peptides for increased resistance towards proteases and proteases inhibitors design [119].

The modulation of the resistance of peptides towards proteolysis has been mainly investigated with amino acids carrying fluorine atoms at the γ- or ω-position of the side chain [115,116]. Once again, because of their difficult synthesis and incorporation in peptides, α-fluoroalkyl amino acids have rarely been studied. After the pioneer works of Burger and Koksch [117,118], we recently revisited the high potential of these amino acids to circumvent peptide proteolysis. Pepsin generally cleaves peptides at the *C*-terminus of a phenylalanine residue (Phe). By incorporating an α-TfmAla adjacent to the Phe carboxylic group of peptide **77** (Figure 9), hydrolysis was almost inhibited and slow cleavage occurred at another position [102]. A similar peptide incorporating an arginine (Arg) (peptide **78** in Figure 9) has been used as a fluorinated tag in a 3-FABS ^19^F NMR-based activity assay to probe the IC_50_ of a trypsin inhibitor (see Section 3.5.2) [102]. Very recently, Pytkowicz et al. incorporated (*R*)- and (*S*)-α-TfmAla at the *N*-terminal position of pepstatin A based inhibitors of cathepsin D and pepsin (peptides **79** and **80** in Figure 9) [119]. The inhibitory potency of these pepstatin analogues was measured towards isolated cathepsin D and pepsin using a classical FRET-based assay. Both peptides presented nanomolar IC_50_ values. However, they were slightly less active and selective than pepstatin.

### 3.3. Conformationnal Aspects of α-Fluoroalkyl-α-Amino Acids and Their Peptides

#### 3.3.1. α-Fluoroalkyl Amino Acid Conformations

- Acyclic amino acids

The introduction of fluorine atoms or fluorinated groups into amino acids proves very useful either for controlling the conformation of the side chain of the amino acid itself and/or for adjusting the conformation of peptides and peptidomimetics or proteins. As a result, the use of fluorinated amino acids for controlling conformation already serves a wide range of valuable applications.

**Figure 10:**
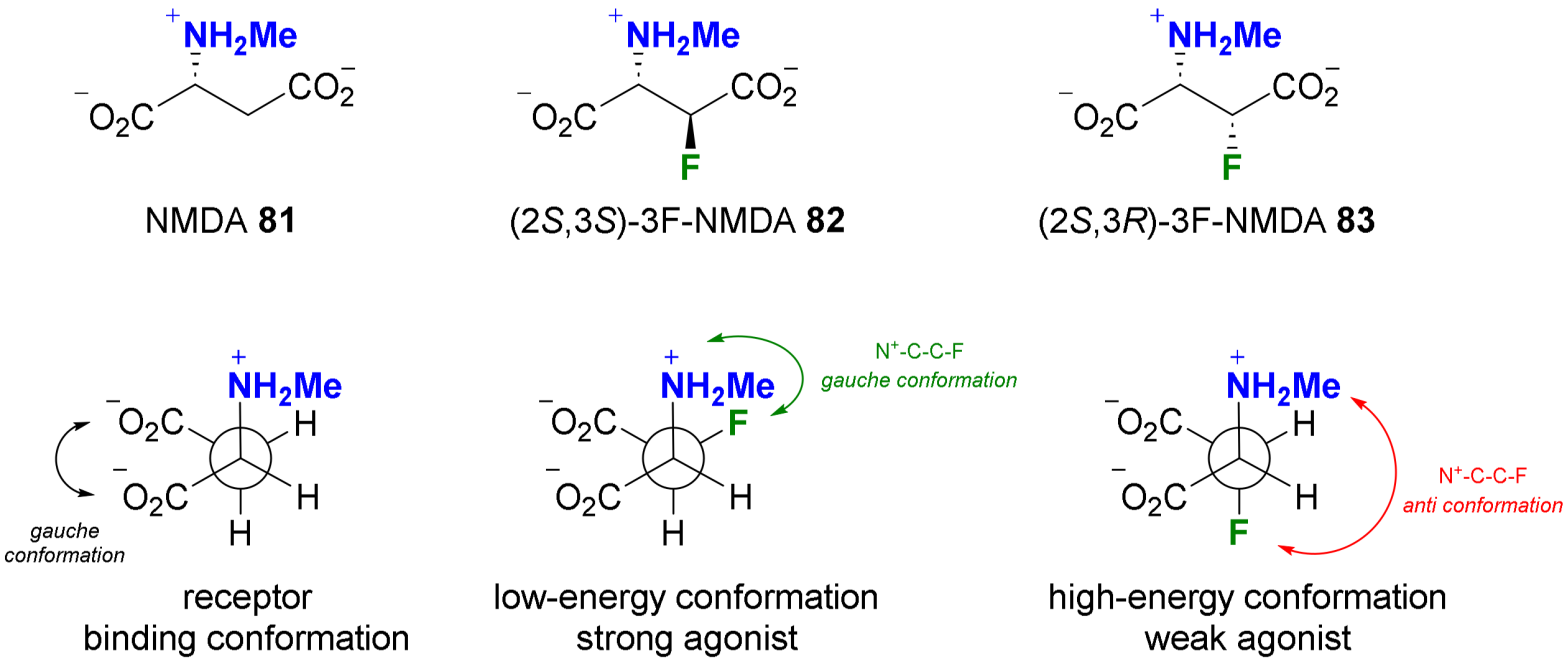
Fluorination of NMDA to control the binding conformation to the glutamate receptors [19].

In the case of fluorinated amino acids containing one or several monofluorinated methylene groups, the relative orientation of the polar C-F bond adapts predictably with those of the neighboring functional groups. Thus, due to an electrostatic attraction, molecules containing the N^+^-C-C-F moiety preferentially adopt a conformation in which the F^δ−^ and the N^+^ atoms are *gauche*. Such an electrostatic effect has been successfully used to control the conformation of the glutamate receptor agonist (*S*)-*N*-methyl-D-aspartate (NMDA) side chain and influence its binding efficiency to the glutamate receptor (Figure 10) [19]. The stereogenic incorporation of a C-F bond at the C-3 position of NMDA to generate (2*S*,3*S*)-3F-NMDA **82** and (2*S*,3*R*)-3F-NMDA **83** gave rise to very different levels of agonist potency on glutamate receptors. Compound (2*S*,3*S*)-**82** displayed similar agonist activity to native NMDA, while (2*S*,3*R*)-**82** was almost inactive. The reason was that (2*S*,3*S*)-**82** preferentially adopted a binding conformation similar to that of NMDA **81**, in which the C-F and the C-N^+^ substituents are *gauche*. On the other hand, (2*S*,3*R*)-**83** would have to orient the C-F and the C-N^+^ substituents *anti* in a high-energy conformation to adopt the NMDA binding conformation.

In the case of molecules containing a F-C-C-H moiety, the favored conformation is obtained by positioning the C-F and the C-H bonds in an *anti*-periplanar orientation, due to σ_CH_ → σ*_CF_ hyperconjugation interactions. This conformational effect has been successfully applied to proline residues to stabilize the conformation of the pyrrolidine ring [26,27,29,120,121].

- Cyclic amino acids

**Figure 11:**
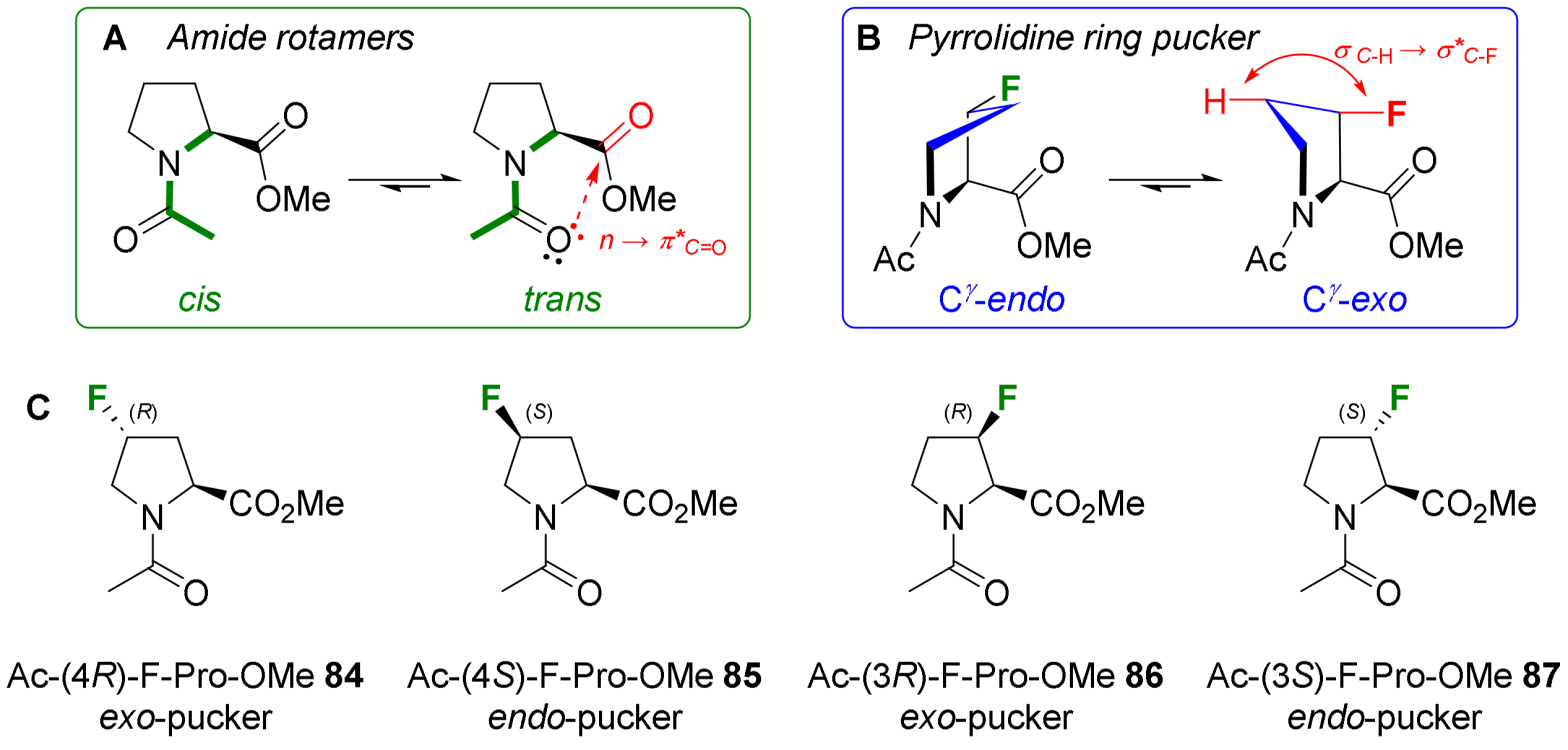
(**A**) *Cis*/*trans* conformer isomerization within the peptidyl–proline amide bond. (**B**) Interconversion between *endo* and *exo* pyrrolidine ring puckers of **85**. (**C**) Chemical structures of 3- and 4-monofluorinated l-proline models **84–87** with their conformational biases [124].

Monofluorination at the C3- or C4-position of the pyrrolidine ring makes it possible to stabilize either the C*^γ^*-*exo* or the C*^γ^*-*endo* conformation, depending on the stereochemistry of the fluorine atom. The preference for a specific ring pucker conformation can be explained by the adoption of a *gauche* conformation between the amide nitrogen and the vicinal fluorine atom in an N−C−C−F arrangement [122]. Studies on model Ac-F-Pro-OMe peptides revealed that the *exo* ring pucker stabilizes the *trans* amide bond [123] through an enhanced n → π* interaction between the amide oxygen and the ester carbonyl carbon (Figure 11) [124]. X-ray diffraction studies demonstrated that the 3- and 4-fluoroprolines series displayed the same C*^γ^* ring pucker preferences, e.g., both Ac-(3*R*)-F-Pro-OMe **86** and Ac-(4*R*)-F-Pro-OMe **84** favor the C*^γ^*-*exo* conformation, with a high K*_trans_*_/*cis*_ equilibrium constant [120,121]. However, it is noteworthy that Ac-(3*R*)-F-Pro-Ome **86** and Ac-(4*R*)-F-Pro-Ome **84** displayed opposite orientations of the fluoro substituent with respect to the fixed l-configuration of C*^α^*, *syn*, and *anti* orientations, respectively. Such a structural feature has been applied in peptides to demonstrate that the energetics of elastin assembly can be altered through a combination of stereoelectronic and steric effects [120]. Another elegant example was the replacement of the proline residue (Pro) by either (4*S*)-F-Pro or (3*S*)-F-Pro to explain the origins of the extraordinary thermostability of collagen, which forms triple helices from Pro-Hyp-Gly repeats [29].

The conformational preferences of Ac-(3*S*)-F-Pro-OMe **87** and Ac-(3*R*)-F-Pro-OMe **86** obtained by computational analyses were identical to those observed in the crystal structures [29]. However, the thermodynamic preference of Ac-(3*S*)-F-Pro-OMe **87** for the C*^γ^*-*endo* conformation (~97% population) was significantly greater than that of Ac-(3*R*)-F-Pro-OMe **86** for C*^γ^*-*exo* (~69% population). The steric congestion surrounding the C^β^ atom observed in the crystal structure of **86** may account for the weaker conformational preference.

The inductive effects of the fluorine decreased the ability of the nitrogen lone pair to contribute to the double bond character of the amide bond. Thus, 3-fluoroprolines exhibited a lower rotational energy barrier and, consequently, accelerated isomerization kinetics compared to the proline residue. Interestingly, the entropic barrier of Ac-(3*R*)-F-Pro-OMe **85** was two-fold greater than Ac-(3*S*)-F-Pro-OMe **87**, suggesting that a fluorine in the *syn* configuration within the pyrrolidine ring may sterically hinder the rotation around the C−N bond [121].

**Figure 12:**
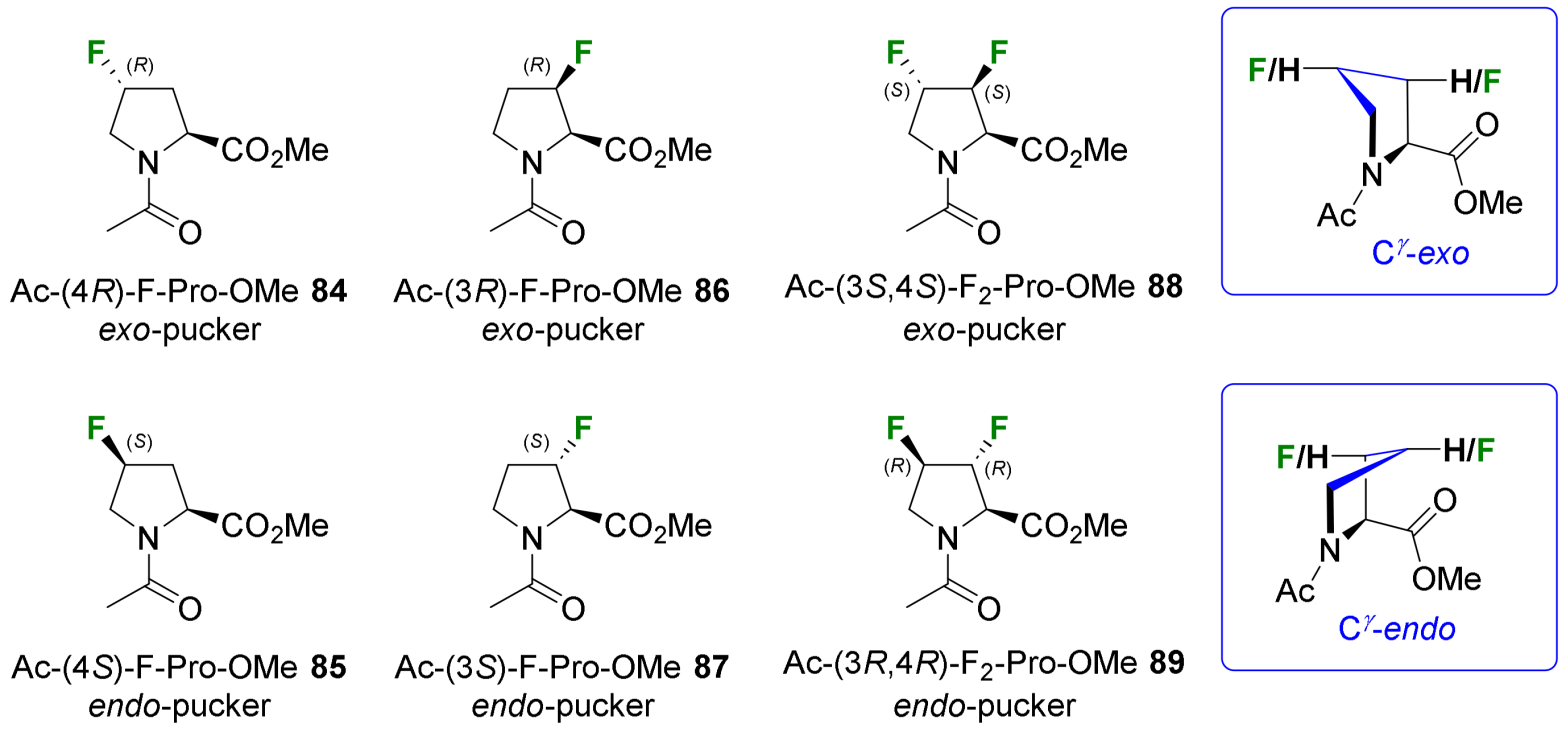
Chemical structures of fluorinated l-proline models **84**–**89** with their conformational biases [27].

The introduction of a second fluorine atom at a vicinal position in the proline ring was of particular interest to introduce compensating conformational bias enabling the design of non-invasive ^19^F NMR probes that do not interfere with protein folding kinetics. However, vicinal fluorination can undergo matching or mismatching effects, depending on the relative stereochemistry. Due to the opposing stereoelectronic effects instilled by the individual fluorine atoms, the (3*S*,4*R*)-difluorinated proline analogue containing a vicinal *cis*-difluoro motif displayed a minimal conformational bias to proline [26]. In contrast, with proline derivatives bearing a vicinal *trans*-difluoro motif, the fluorine atoms exhibited similar preorganizing effects. As a result, the (3*R*,4*R*)-difluorinated proline (3*R*,4*R*)-3,4-F_2_-Pro **89** and (3*S*,4*S*)-difluorinated proline derivatives and (3*S*,4*S*)-3,4-F_2_-Pro **87** show similar conformational preferences to those of (4*S*)-F-Pro **85** and (4*R*)-F-Pro **84**, respectively (Figure 12) [27].

As expected, the introduction of an additional fluorine atom at the vicinal position increased the *cis/trans* isomerization rate. Both the (3*R*,4*R*)-3,4-F_2_-Pro **89** and (3*S*,4*S*)-3,4-F_2_-Pro **87** derivatives showed higher isomerization kinetics than their monofluorinated progenitors [27]. It is noteworthy that (3*R*)-F-Pro **86** showed a significantly higher isomerization rate than all other monofluorinated prolines, as well as (3*R*,4*R*)-3,4-F_2_-Pro **89**. The (3*S*,4*S*)-3,4-F_2_-Pro **88** analogue, for which the stereochemistry at the 3-position was retained with respect to the (3*R*)-F-Pro **86**, exhibited even higher isomerization rates than those observed for the (3*S*,4*R*)-3,4-F_2_-Pro and the (4,4)-difluorinated variants [26].

**Figure 13:**
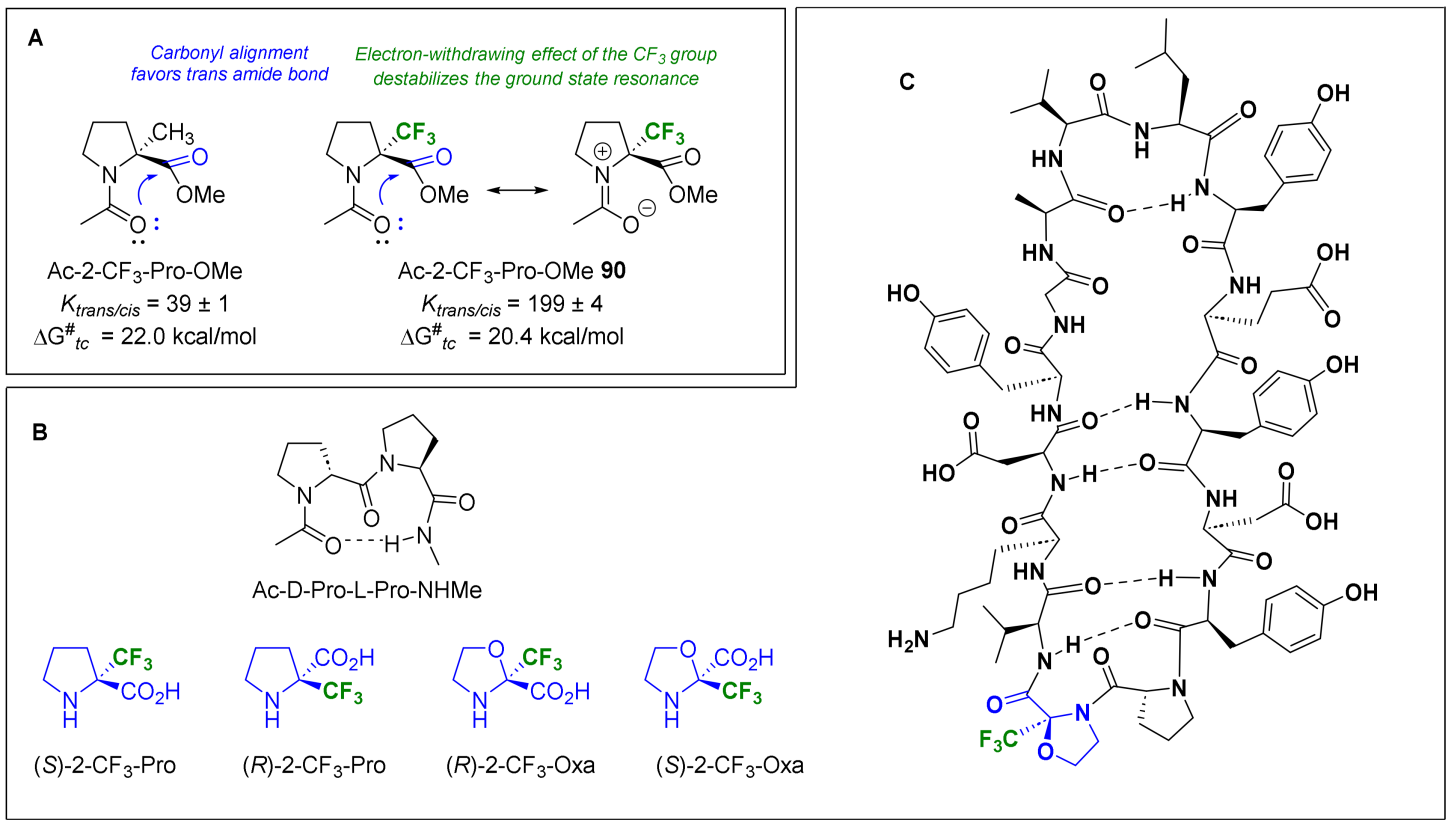
(**A**) *Cis-trans* ratio and rotational energy barrier of Ac-CH_3_-Pro-OMe and Ac-CF_3_-Pro-OMe peptide models [107]. (**B**) Chemical structures of 2-CF_3_-Pro and 2-CF_3_-Oxa residues to improve the conformational stability of the d-Pro-l-Pro template [125]. (**C**) Chemical structure of the fluorinated CDR3 Nb80 cyclic mimetic [125].

Although several synthetic routes have been reported for the preparation of the α-trifluoromethylproline (2-CF_3_-Pro), its incorporation into peptides is hampered by the low nucleophilicity of the amino group and the steric hindrance of the neighboring CF_3_ group. Therefore, little is known about the conformational consequences of the incorporation of the α-CF_3_-Pro residue. Comparative effects of CF_3_ and CH_3_ group substitutions on model Ac-R-Pro-OMe peptides (with R = CF_3_ or CH_3_) revealed that the presence of a bulky group at the position 2 strongly impacted the *trans*/*cis* amide rotameric preference (Figure 13A) [107]. The larger size of the CF_3_ group compared to the CH_3_ group, together with additional contribution of the inter-carbonyl alignment, led to additional stabilization of the *trans* amide rotamer for Ac-2-CF_3_-Pro-OMe **90**. Moreover, the presence of the electron-withdrawing CF_3_ group in **89** decreased the rotational energy barrier by destabilizing the ground state resonance.

#### 3.3.2. α-Fluoroalkyl Amino Acids as Tools for the Control of Peptide Conformations

The 2-CF_3_-Pro compound and its oxazolidine analogue (2-CF_3_-Oxa) have been investigated for their abilities to improve the conformational stability of the d-Pro-l-Pro template, a sequence frequently used in the design of β-hairpin peptidomimetics (Figure 13B) [125]. An in silico conformational study allowed the dipeptide d-Pro–(*R*)-2-CF_3_-Oxa to be identified as the most promising surrogate with an increased *trans*–*cis* rotational energy barrier. By grafting the d-Pro-l-Pro sequence to the CDR3 loop of the nanobody Nb80, the corresponding cyclic peptide presented a large conformational heterogeneity, while the incorporation of the d-Pro–(*R*)-2-CF_3_-Oxa showed a dominant conformation (Figure 13C).

C^α^-tetrasubstituted α-amino acids, such as α-aminoisobutyric acid (Aib), are non-proteinogenic α-amino acids, in which the α-hydrogen atom is replaced with an alkyl substituent. Due to their conformational restriction, these residues are reported to be strong inducers of helical conformations, such as α-helix, 3_10_-helix, or planar 2.05-helix, when incorporated into peptides.

**Figure 14:**
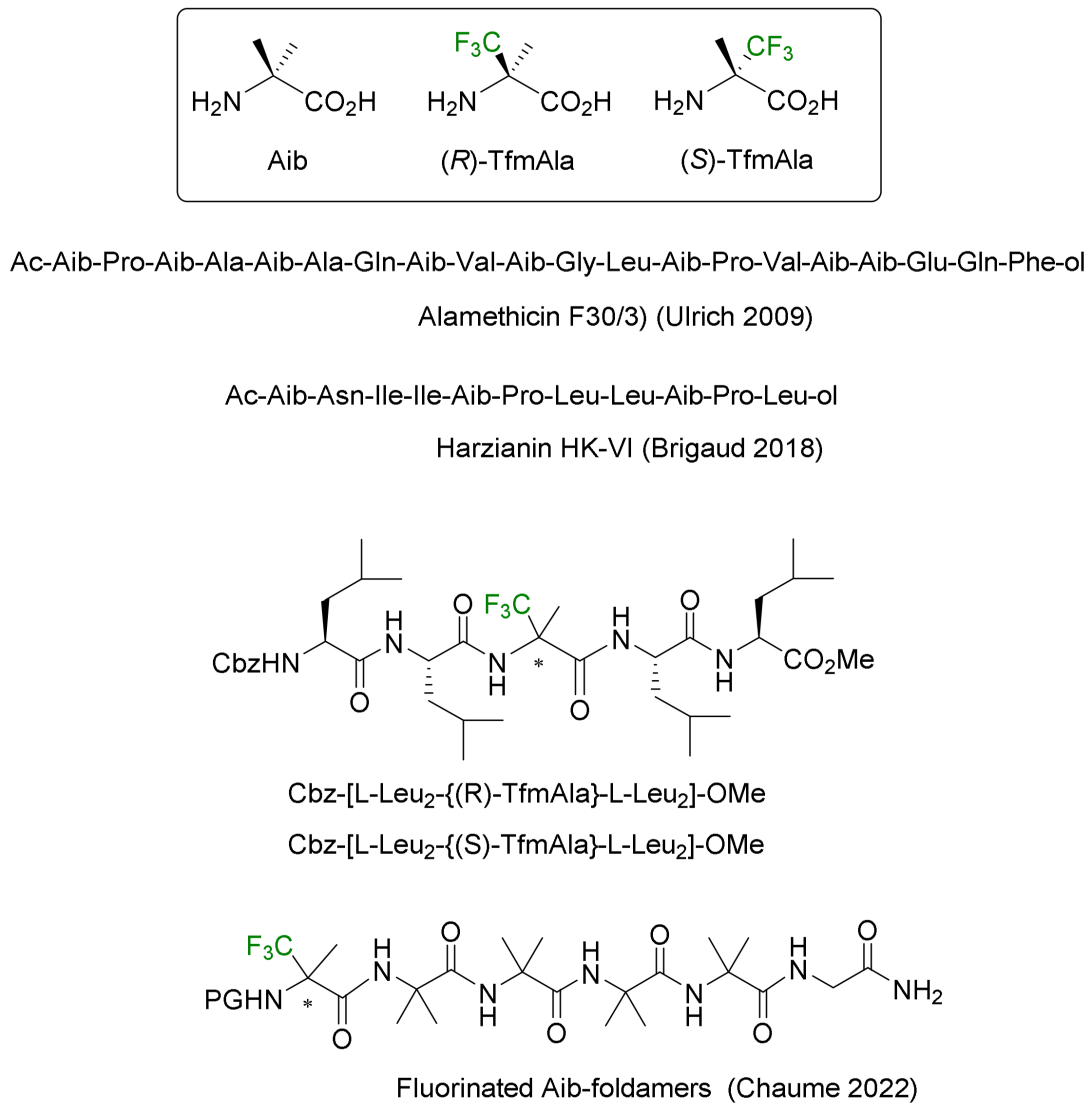
Chemical structures of Aib and TfmAla residues and their relevant applications in peptide chemistry [103,104,127].

The use of α-trifluoromethylalanine (TfmAla) as chiral fluorinated analogue of Aib has been reported in several examples. Ulrich et al. reported the used of (*R*)-TfmAla and (*S*)-TfmAla as orthogonal ^19^F-labels to elucidate the backbone conformation and the helix alignment in lipid bilayers of two peptaibols, namely, alamethicin F30/3 [104] and harzianin HK-VI [103], by solid-state NMR spectroscopy. A set of fluorinated analogues of the two peptaibols was synthetized by substituting each Aib residue one-by-one with (*R*)-TfmAla and (*S*)-TfmAla, respectively. All the fluorinated analogues gave the same circular dichroism (CD) line shape as that of the wild type in various membrane mimics, confirming that the secondary structure remained unperturbed. In contrast, Tanaka et al. reported that l–Leu-based pentapeptides with (*R*)-TfmAla and (*S*)-TfmAla formed similar (*P*) right-handed 3_10_-helical structures in the solid state, but with different backbone conformations for the *C*-terminal residues [126]. The authors suggested that both the hydrophobicity and electronegativity of the CF_3_ group in (*R*)-TfmAla and (*S*)-TfmAla differently affected the *C*-terminal residues (Figure 14).

Chiral C^α^-tetrasubstituted α-amino acids also proved to be useful tools to control the handedness of helical structures, in particular when incorporating into achiral Aib oligomers. (*R*)-TfmAla and the (*S*)-TfmAla have been employed as chiral controllers to induce the screw-sense preference of short Aib-based oligomers [127]. NMR studies and X-ray crystallography confirmed the stabilization of the 3_10_ helical structure. Interestingly, the selectivity of the screw-sense was found to be reversed compared to that induced by the non-fluorinated l-α-MeVal chiral inducer due to the electronic properties of the CF_3_ group that favors a dipole alignment (Figure 14).

### 3.4. α-Fluoroalkyl-α-Amino Acids as [^18^F]-PET Tracers

**Figure 15:**
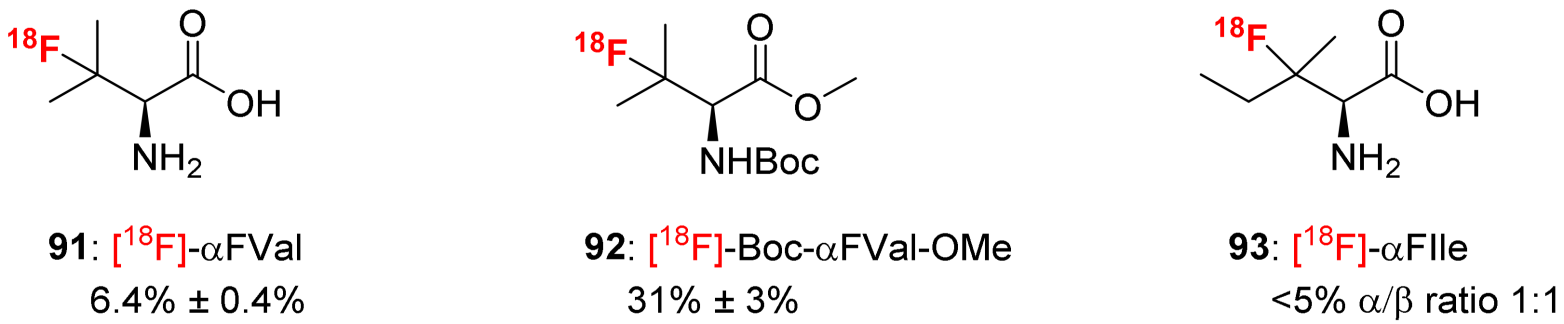
[^18^F]-α-fluoroalkyl-α-amino acids with radiochemical yields [135,136].

Positron emission tomography (PET) is a non-invasive molecular in vivo imaging technique that uses radiotracers to visualize and measure changes in metabolic processes and other physiological activities, including blood flow, regional chemical composition, and absorption [128]. PET, alone or coupled with other techniques, such as MRI and CT, find applications in different areas of medicine, such as cardiology, neurosciences, drug development, and, notably, oncology [129]. Among all the radionuclides employed for PET, [^18^F] is the most popular due to its suitable half-life (109.7 min), its high positron decay ratio (97%), and its low positron energy (maximum 0.635 MeV) [130]. [^18^F]-FDG ([^18^F]-2-deoxy-2-fluoro-d-glucose) is one of the most widely used fluorinated PET radiotracer, since, as an analogue of d-glucose, it is an excellent biomarker of metabolism [131]. However, [^18^F]-FDG has selectivity issues, since it cannot differentiate between increased metabolic rates due to pathological conditions versus infection, inflammation, and tissues with normally increased physiological uptake [132]. For this reason, the scientific community is focused on developing more selective [^18^F]-FDG radiotracers targeting specific biomarkers [133]. In this context the development of [^18^F]-labeled AAs found its place, especially within the brain oncology field. [^18^F]-FDG uptake in the brain is high even under physiological conditions; therefore, it is challenging to obtain images of brain cancers with an acceptable definition and resolution. AAs show low basal uptake in tissues with high glucose uptake, such as the brain and inflamed or infected tissues. Moreover, many brain tumors, in addition to increased glucose consumption, show an important uptake of AAs as complementary source of nutrients [134]. AAs are transported into the brain via l-type amino acid transporters (LATs), which are also overexpressed in different cancers. Thus, LATs represent a promising target, and [^18^F]-AAs are potential radioprobes for PET cancer imaging. Most known [^18^F]-fluorinated AAs are aromatic, such as tyrosine and phenylalanine, since the fluorination on the ring is easily performed via nucleophilic aromatic substitution (SNAr). Nevertheless, two examples of [^18^F]-α-fluoroalkyl-α-amino acids as potential PET tracers have also been reported: unprotected/protected [^18^F]-α-fluorovaline (**91** and **92**) and [^18^F]-α-fluoroisoleucine (**93**) (Figure 15). In 2017, the Groves group developed a manganese-catalyzed [^18^F] fluorination method for aliphatic compounds based on direct C-H activation [135]. Using a manganese (III) pentafluorophenyl porphyrin coordinated with a tosyl ligand as the catalyst and iodosylbenzene as the terminal oxidant, Groves obtained the *N* (boc)- and *O* (methyl ester)-protected [18F]-α-fluorovaline (**93**) with a radiochemical conversion of 31 ± 3%. In the same period, Britton and coworkers published the [^18^F]-fluorination of unprotected branched aliphatic AAs, including the synthesis of [^18^F]-α-fluorovaline (**91**) and the [^18^F]-α-fluoroisoleucine (**93**) [136]. The photocatalyzed radio fluorination of Britton involves activated (360 nm) NaDT (sodium decatungstate) as a hydrogen atom-abstracting agent in combination with the fluorine atom donor [^18^F]NFSI (*N*-fluorobenzenesulfonimide). According to this method, **91** was obtained in 6.4 ± 3% radiochemical yield. Britton also attempted the synthesis of **93**. However, an inseparable mixture of α- and β-fluorinated isoleucine was obtained in a low yield (<5%). None of the synthesized [^18^F]-α-fluoroalkyl-α-amino acids, alone or inserted in a peptide scaffold, were tested in vivo. In conclusion, [^18^F] labeling of AAs is still challenging, and novel radio fluorination methods with a short synthesis time and safe agents are urgently needed.

### 3.5. α-Fluoroalkyl-α-Amino Acids as ^19^F NMR Probes

#### 3.5.1. NMR Sensitivity of ^19^F and Its Opportunities

The fluorine atom represents an excellent NMR probe for several reasons [137]. First of all, fluorine is nearly totally absent in proteins and biomolecules; thus, there are no background signals to take in consideration. Moreover, ^19^F has a 100% isotopic abundance and high NMR sensitivity (83% the sensitivity of ^1^H). Furthermore, the ^19^F chemical shift is very sensitive to the environment, and the chemical shift variations can be detectable even with subtle differences in the molecule’s structures. Finally, the ^19^F chemical shift range is extremely wide, 100-fold larger than that of ^1^H (>500 ppm). These two last features result in a low probability of signal overlap even in the case where multiple copies of the same fluorinated amino acid are present. Finally, in most cases, substituting a proton with a fluorine in an amino acid’s side chain does not dramatically alter the molecule’s properties. Consequently, ^19^F NMR can be used as an invisible spy to interrogate biomacromolecules’ properties and functions. In this context, fluorinated AAs have an essential role in interrogating the properties and functions of a biomolecule through ^19^F NMR spectrometry. Incorporating a fluorinated AA into a biomolecule, such as a protein, permits the investigation of different phenomena, such as protein–protein/protein–ligand and protein–lipid interactions, protein unfolding/folding, and protein aggregation. On the other side, incorporating a fluorinated AA into a single peptide, such as a substrate or a ligand, allows ^19^F NMR-based competition assays, biochemical screening, and magnetization transfer experiments to be performed [138]. Fluorinated AAs are also an important tool to study, by solid-state ^19^F NMR, membrane-bound peptides, investigating their mechanism of actions, interactions, and conformation [139]. The most common non-canonical AAs employed as ^19^F NMR probes present an aromatic ring, such as tryptophan, tyrosine, or phenylalanine. Regarding the aliphatic AAs, leucine and serine analogue are the most popular [140]. Nevertheless, some examples of α-fluoroalkyl-α-amino acids employed as ^19^F NMR spies are reported and detailed below.

#### 3.5.2. α-Trifluoromethylalanine (α-TfmAla)

The first example of α-trifluoromethylalanine used as an ^19^F NMR probe was reported in 2009 by Ulrich, Koksch et al. [104]. (*R*)- and (*S*)-α-TfmAla were incorporated in the scaffold of fungal peptaibol, peptides with a high content of unconventional AAs, and alamethicin in the place of Aib (α-aminoisobutyric acid) groups. Six different fluorinated analogues of alamethicin were synthesized. Circular dichroism spectra and antimicrobial and hemolytic assays confirmed that all the substitutions were essentially non-perturbing in terms of activity and structuration. A solid-state ^19^F NMR analysis was then used to study the alignment and conformation of alamethicin into a liquid-crystalline lipid bilayer membrane.

In 2018, Ulrich, Brigaud et al. took advantage of (*R*)-α-TfmAla and (*S*)-α-TfmAla to develop a general solid-state NMR approach to study the interaction, orientation, and structure of membrane-bound short peptides [103]. Peptaibols often exhibit cytotoxic activity, presumably due to their capability to permeate the cell membrane. To fully understand the mechanism of actions of these biomolecules, it is critical to investigate how they bind to the lipid membrane and which conformation and orientation they adopt. To elucidate the unknown structure of the short peptaibol HZ in its functionally relevant membrane-bound state, Ulrich, Brigaud and coworkers synthesized different analogues of HZ using four orthogonal ^19^F and ^15^N NMR probes, including the two enantiomers of α-TfmAla. Using solid-state NMR spectroscopy, the backbone structure and alignment states of HZ were determined in different types of membranes.

**Figure 16:**
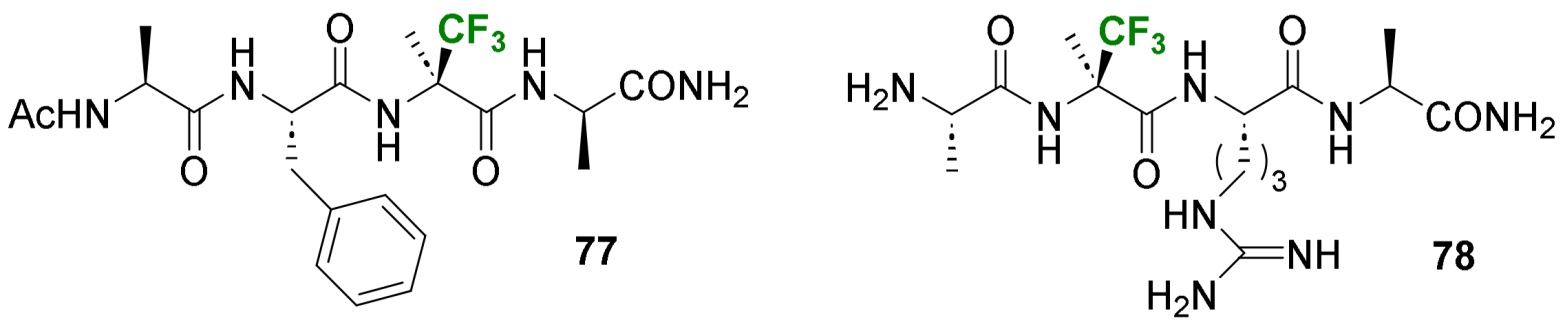
Structures of fluorinated peptides **77** and **78 [102]**.

In 2001, α-TfmAla was also employed as fluorinated probe by Pytkowicz, Brigaud et al. to develop a ^19^F NMR enzymatic assay [102]. Pytkowicz and coworkers synthesized two peptides incorporating the non-natural α-quaternarized (*R*)-α-TfmAla (**76** and **77**, Figure 16). The fluorinated peptides **76** and **77** were used as substrates to monitor the proteolytic activity of two different enzymes, pepsin and trypsin, respectively, by means of ^19^F NMR 3-FABS experiments (n Fluorine Atoms for Biochemical Screening). The peptides were incubated with the enzymes directly in the NMR tube in the absence or in the presence of an inhibitor. The digestion of the substrates was monitored by ^19^F NMR; the signal of the peptides decreased in intensity, and a new signal generated from enzymatic cleavage appeared and increased in intensity. A 2D ^19^F DOSY experiment was also employed to characterize the products resulting from the enzymatic digestion of the fluorinated peptides. Using (*R*)-α-TfmAla as an NMR probe allowed the accurate and rapid measurement of the IC_50_ of a classical trypsin inhibitor and tests of the metabolic resistance of the substrates towards the pepsin. The presence of (*R*)-α-TfmAla in the peptide **78** scaffold allowed its use as a ^19^F NMR probe, permitting the accurate and rapid measurement of the IC_50_ of a well-known trypsin inhibitor. (*R*)-α-TfmAla was also fundamental to evaluate, always via ^19^F NMR, the metabolic resistance of peptide **77** towards pepsin.

**Figure 17:**
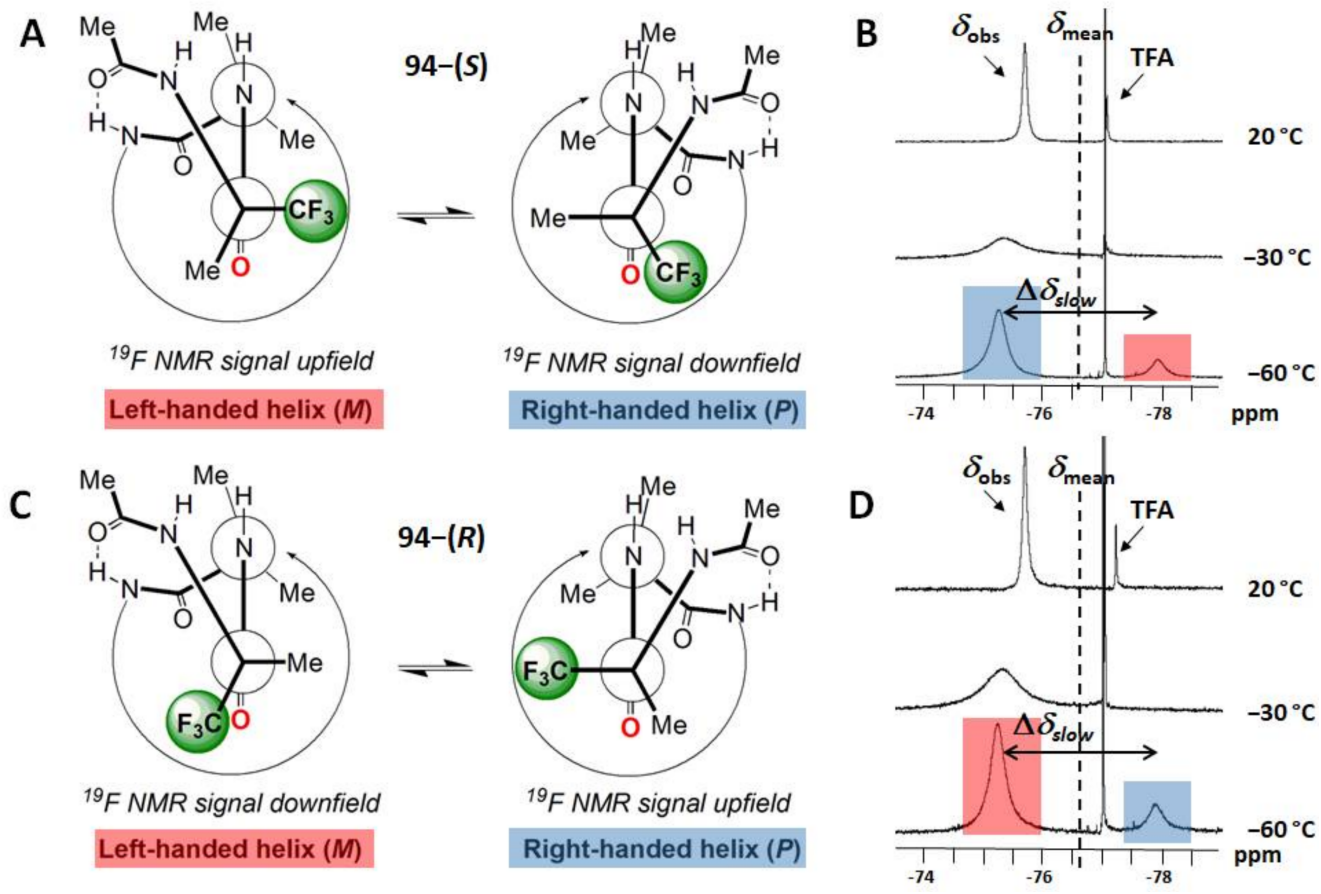
Newman projections of the *N*-terminal turns of **95**-(*S*) (**A**) and **95**-(*R*) (**C**) in left-handed and right-handed helices with the ^19^F NMR spectra in CD_3_OD (**B**,**D**) at three different temperatures [127].

The two enantiomers of the α-TfmAla were also recently employed as ^19^F NMR reporters to quantify and assign the helical screw-sense in a series of Aib foldamers [127]. For the quantification of the screw-sense preference, the Cbz-(*S*)-α-TfmAla-Aib_4_-Gly-NH_2_ **94**-(*S*) and the Ac-(*S*)-α-TfmAla-Aib_4_-Gly-NH_2_ **95**-(*S*), as well as their (*R*) analogues **94**-(*R*) and **95**-(*R*), were analyzed at low temperature by ^19^F NMR. At −60 °C, thanks to the slow regime exchange, two distinct peaks corresponding to the CF_3_ resonances of the (*M*)- and (*P*)-helices were identified. The helical excesses were calculated by integrating the signals. The assignment of the sign of the helix was based on the diverse chemical shifts of the CF_3_ group of the chiral α-TfmAla residue. This is due to its different environments when it is incorporated in the right- or left-handed helix. Circular dichroism spectra were also recorded to permit the ^19^F NMR assignment of the right-handed (*P*)-helix (downfield signal) and the left-handed (*M*)-helix (downfield signal) for all four fluorinated foldamers (Figure 17). Moreover, the energy barrier of the helix interconversion was also determined using ^19^F NMR.

#### 3.5.3. 3-Fluorovaline, 3-Fluoroalanine, and (*S*) 3-Trifluoroalanine

**Figure 18:**
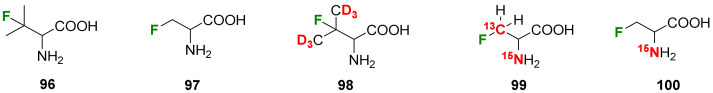
Fluorinated aliphatic AAs as a sensitive ^19^F, ^13^C, ^15^N, and ^2^H NMR reporters to study the structures and interactions of proteins [141].

In 2022, Otting and coworkers developed a series of fluorinated aliphatic AAs as sensitive NMR reporters for studies of biomolecules (**96**–**100**) in a racemic mixture (Figure 18) [141]. Once incorporated into a protein, these unnatural AAs could be used to investigate its structure, dynamics, and its interaction with other proteins and ligands. Therefore, they represent valuable tools in structural biology, medicinal chemistry, and drug discovery. In more detail, among other fluorinated AAs, the Otting group synthesized 3-fluorovaline **96** and 3-fluoroalanine **97**, as well as their ^13^C, ^15^N, and ^2^H isotopologs (**98**, **99**, and **100**). The fluorinated, labeled analogs could allow 2D heteronuclear NMR experiments to be performed and the consequent acquisition of more information on the studied target. To assess the potential of fluorinated aliphatic AAs as ^19^F NMR probes, the syntheses of the GB1 protein incorporating **96** and **97** were achieved. The expression of the protein was performed cell-free to allow the incorporation into GB1 of 3-fluoroalanine **97**, which is highly toxic to bacteria in vivo. The recorded mass spectra of the fluorinated proteins showed an incomplete substitution of the canonical amino acid with **96**, while **95** was highly incorporated. The 1D ^19^F NMR spectra showed the structural conservation of the modified proteins. The wide range of different chemical shifts observed in the ^19^F NMR spectra of the proteins demonstrated once again the vast spectral resolution available in ^19^F NMR spectra, corroborating the use of aliphatic fluorinated AAs **96** and **97** as ^19^F NMR probes.

The Ulrich group deeply characterized a series of fluorinated aliphatic AAs using solid-state NMR, comparing them with a set of fluorinated aromatic and canonical AAs [142]. This study adds an essential piece of knowledge to the properties of fluorinated noncanonical AAs in view of their use as NMR probes.

#### 3.5.4. 3-Fluoroprolines, 3,4-Difluoroprolines, 2-Trifluoromethyl Prolines

**Figure 19:**
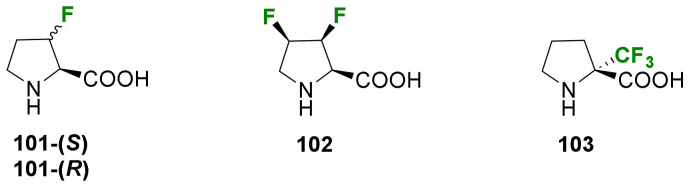
Fluorinated proline as a potential non-invasive ^19^F NMR probe.

Fluorinated prolines could also represent a sensitive reporter for ^19^F NMR studies of protein conformations, dynamics, and interactions. The well-known 4-fluoroprolines, once incorporated into biomolecules, are widely used to modify their conformations. In 4-fluoroprolines, the fluorine is no longer a spectator but induces conformational modifications to the molecule structure (different puckers of the five-membered ring and *cis*/*trans* ratios). This interesting conformational aspect, exploited for a wide range of applications, is a hindrance when the natural proline conformation is required and limits the possibilities of using 4-fluoroprolines as unbiased NMR reporters. Nevertheless, fluorination in different positions of the proline scaffold could minimize or avoid this effect, representing an exciting strategy for developing a non-invasive proline-based ^19^F NMR probe. In this context, α-fluoroalkyl-α-prolines, such as **101**, **102**, and **103** (Figure 19), could represent an exciting option [123]. 3-Fluoroproline **101** shows a relatively small impact on the *K_trans/cis_* equilibrium constant compared with 4-fluoroprolines, making them potential NMR reporters. In 3-4-difluoroproline **102**, the introduction of a second fluorine atom in position 3 help to maintain the “natural” proline ring pucker and amide conformations [26]. In addition, the presence of two fluorine atoms could represent an opportunity to double-label proteins and peptides with different characteristic ^19^F reporter signals. Finally, α-trifluoromethylproline **103**, which is significantly more “native proline-like” in terms of backbone conformational propensities, the *K_trans/cis_* equilibrium constant, and modification of structural conformations of peptides, is also an attractive option. Moreover, in the case of **102**, three symmetrical fluorine nuclei could provide a higher signal-to-noise ratio, offering an enriched sensitivity in ^19^F NMR.

## 4. Conclusions

The synthesis of α-fluoroalkyl-α-amino acids in enantiopure form is still highly challenging and there is a great need for new, robust stereoselective methods for their synthesis. As future perspectives and developments, enantioselective catalytic methods for fluorination and fluoroakylation of prochiral precursors deserve to be studied. For example, the development of direct di- and trifluoromethylation of amino acid precursors would constitute a significant advance, as well as the selective late-stage fluorination and fluoroalkylation of peptides. From a synthetic point of view, the incorporation of α-fluoroalkyl-α-amino acids in peptides by SPPS in good yields is a limit and needs to be developed, especially for trifluoromethyl compounds. The site-specific bio-incorporation of fluorinated amino acids in peptides and proteins would also constitute a major breakthrough. However, deactivation of the amino group combined with steric hindrance may make this task very difficult. To our knowledge, all the preliminary attempts to incorporate these α-fluoroalkyl-α-amino acids in peptides by biological methods have failed so far. This challenge is hampering the development of the use such fluorinated amino acids in proteins. Nevertheless, it has already been demonstrated and reported in this review that α-fluoroalkyl-α-amino acids and their peptides display highly promising opportunities for medicinal chemistry. Indeed, compared to their analogues bearing fluorine further on the aliphatic chain, α-fluoroalkyl amino acids are highly resistant to proteolysis. It is anticipated that the development of new, efficient synthetic methods and the rise of chemical biology will stimulate new applications such as the use of fluorinated amino acids as ^19^F NMR probes for biological process investigations

## Data Availability

No new data were created. Data sharing is not applicable to this article.
